# The efficacy of virtual reality for upper limb rehabilitation in stroke patients: a systematic review and meta-analysis

**DOI:** 10.1186/s12911-024-02534-y

**Published:** 2024-05-24

**Authors:** Mohsen Soleimani, Marjan Ghazisaeedi, Soroush Heydari

**Affiliations:** Department of Health Information Management and Medical Informatics, School of Allied Medical Sciences, Tehran University of Medical Sciences, Tehran, Iran

**Keywords:** Stroke, Upper limb, Virtual reality, Rehabilitation, Immersion, Systematic review, Meta-analysis

## Abstract

**Background:**

Stroke frequently gives rise to incapacitating motor impairments in the upper limb. Virtual reality (VR) rehabilitation has exhibited potential for augmenting upper extremity recovery; nonetheless, the optimal techniques for such interventions remain a topic of uncertainty. The present systematic review and meta-analysis were undertaken to comprehensively compare VR-based rehabilitation with conventional occupational therapy across a spectrum of immersion levels and outcome domains.

**Methods:**

A systematic search was conducted in PubMed, IEEE, Scopus, Web of Science, and PsycNET databases to identify randomized controlled trials about upper limb rehabilitation in stroke patients utilizing VR interventions. The search encompassed studies published in the English language up to March 2023. The identified studies were stratified into different categories based on the degree of immersion employed: non-immersive, semi-immersive, and fully-immersive settings. Subsequent meta-analyses were executed to assess the impact of VR interventions on various outcome measures.

**Results:**

Of the 11,834 studies screened, 55 studies with 2142 patients met the predefined inclusion criteria. VR conferred benefits over conventional therapy for upper limb motor function, functional independence, Quality of life, Spasticity, and dexterity. Fully immersive VR showed the greatest gains in gross motor function, while non-immersive approaches enhanced fine dexterity. Interventions exceeding six weeks elicited superior results, and initiating VR within six months post-stroke optimized outcomes.

**Conclusions:**

This systematic review and meta-analysis demonstrates that adjunctive VR-based rehabilitation enhances upper limb motor recovery across multiple functional domains compared to conventional occupational therapy alone after stroke. Optimal paradigms likely integrate VR’s immersive capacity with conventional techniques.

**Trial registration:**

This systematic review and meta-analysis retrospectively registered in the OSF registry under the identifier [10.17605/OSF.IO/YK2RJ].

**Supplementary Information:**

The online version contains supplementary material available at 10.1186/s12911-024-02534-y.

## Introduction

Stroke remains a major global health concern as the second leading cause of mortality and disability worldwide [1]. In 2019, stroke accounted for approximately 11% of all deaths and was the second leading cause of combined death and disability [2]. The risk of stroke rises markedly with age, with 67% of strokes occurring in individuals over 70 years old [3]. Men have a higher incidence compared to women across most age groups [4]. Stroke frequently engenders serious long-term impairment, including hemiplegia, aphasia, vision deficits, and cognitive dysfunction [5]. In particular, upper limb motor dysfunction represents one of the most common and debilitating consequences of stroke, affecting around 80% of survivors [6]. Upper limb motor dysfunction, manifesting as impaired arm and hand function, frequently disrupts the performance of activities of daily living, occupational tasks, and overall quality of life for stroke survivors. Only 5–20% of patients fully recover upper limb function, with nearly half developing chronic spasticity [7]. Effective rehabilitation is therefore paramount for optimizing the recovery of upper limb motor control and performance of functional tasks [8].

Rehabilitation plays a pivotal role in harnessing neuroplasticity and facilitating cortical reorganization, which are essential mechanisms aiding patients in regaining lost skills and fostering the development of compensatory techniques [9]. The significance of early intervention in the rehabilitation process has consistently demonstrated a strong correlation with improved functional outcomes in stroke patients [8]. Within the realm of upper limb rehabilitation, multiple facets warrant meticulous assessment to tailor therapeutic strategies effectively. Several assessment tools have been devised to evaluate distinct dimensions of upper limb recovery. These encompass assessments of motor function, exemplified by the Fugl-Meyer assessment scale (FMA), the Action Research Arm Test (ARAT), and the Wolf Motor Function Test (WMFT). Furthermore, evaluations of independence are essential, with assessments like the Barthel Index (BI) and the Functional Independence Measure serving as valuable instruments. The Box and Block Test (BBT) is a valuable metric for dexterity. Spasticity, a common post-stroke concern, can be assessed using the Modified Ashworth Scale (MAS). Finally, to comprehensively gauge the overall impact of a stroke, the Stroke Impact Scale (SIS) provides a holistic perspective. Incorporating these diverse assessment tools into the rehabilitation process empowers healthcare professionals to tailor interventions precisely, optimizing the recovery trajectory and enhancing the quality of life for stroke survivors.

Upper limb rehabilitation encompasses diverse approaches tailored to patients’ needs after stroke and injury [10]. Physical and occupational therapies provide structured exercises to restore motor skills, range of motion, and activities of daily living [8]. Constraint-induced movement therapy promotes neuroplasticity by restraining the unaffected limb to force the use of the affected limb [11]. Technology-assisted interventions have gained prominence, including virtual reality (VR) with immersive simulated environments for engaging therapy activities, interactive video games that increase motivation, telerehabilitation allowing remote participation for improved access, and robotic devices offering customizable assistance for progressive exercise [6, 12].

VR involves interactive simulation using computer hardware and software to generate immersive 3D environments for rehabilitation activities [12]. VR systems are categorized into three levels of immersion including fully immersive (completely blocking real-world perception), non-immersive (allowing concurrent real and virtual environments), and semi-immersive (using screens or headsets for partial immersion) [12, 13]. Potential advantages of VR for rehabilitation include enhanced user engagement, increased repetitions, and promoted independence [6]; however, challenges remain regarding requisite technology skills, costs, and cyber-sickness [5, 14]. The extant corpus of scholarly research offers preliminary indications regarding the potential efficacy of VR interventions in augmenting upper limb functionality and real-world task performance among individuals afflicted with stroke [15]. Nevertheless, a compelling imperative exists for more exhaustive investigations in this domain. These investigations ought to encompass a diverse array of VR systems and a multitude of upper limb outcome assessment instruments, each designed to discern distinct facets of the post-stroke recovery process.

Despite numerous systematic reviews examining the efficacy of VR systems for upper limb rehabilitation post-stroke [16], salient gaps in the literature remain unaddressed. First, there is a scarcity of meta-analytic studies directly comparing the efficacy of various VR paradigms utilizing different levels of immersion with conventional therapies in improving motor deficits across the entire upper limb post-stroke [17–30]. This obscures conclusions regarding the comparative efficacy of varied levels of VR immersion. Second, extant research has predominantly focused on upper limb activity outcomes, with limited emphasis on VR’s potential to improve performance in all functional autonomy in real-world settings [28]. This restricts the understanding of VR’s broader rehabilitative utility. Finally, a comprehensive appraisal of methodological rigor and risk of bias is lacking in the burgeoning corpus of literature evaluating VR in post-stroke upper limb rehabilitation. While VR shows promise in post-stroke rehabilitation, the rapid pace of technological advances demands rigorous assessment of new VR methods to determine optimal protocols for upper limb recovery. Previous studies have relied heavily on older VR systems and techniques, which may not harness the full potential of modern advancements in the field. The current systematic review and meta-analysis aim to address these limitations by synthesizing studies contrasting different VR systems categorized by immersion level against conventional interventions across various upper limb functional domains. By delineating the effectiveness of diverse VR paradigms compared to traditional approaches across the spectrum of upper limb disability post-stroke, this review seeks to elucidate the optimal utilization of VR to maximize upper extremity recoveries for stroke survivors. The findings shall inform the development of targeted rehabilitation protocols harnessing VR technologies for ameliorating upper limb impairments after stroke.

## Method

**Fig. 1:**
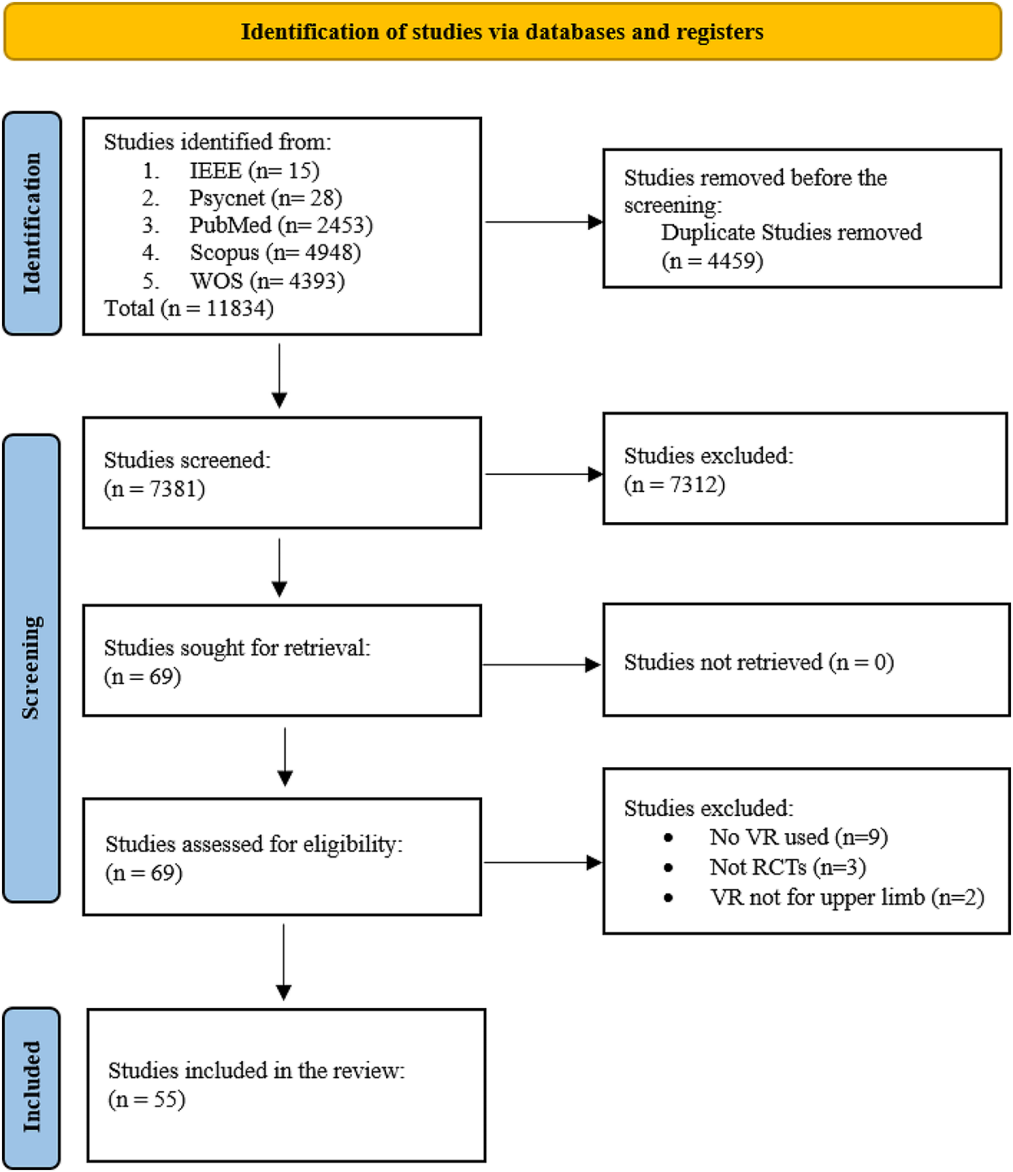
PRISMA flow diagram showing the selection of studies included in this systematic review

This systematic review and meta-analysis followed the PRISMA guidelines [31] (Fig. 1). The primary objective of this study, registered in the OSF registry under the identifier [10.17605/OSF.IO/YK2RJ], was to investigate the potential of VR simulations to improve real-world upper extremity outcomes in stroke patients. The PICO framework (Population, Intervention, Comparison, and Outcome) [32] guided the development of the study question and selection of studies:

Study questions:

1. Should full immersive virtual reality systems vs. conventional therapy be used for upper limb rehabilitation after stroke?
2. Should semi-immersive virtual reality vs. conventional therapy be used for upper limb rehabilitation after stroke?
3. Should non-immersive virtual reality vs. conventional therapy be used for upper limb rehabilitation after stroke?

PICO Framework:

- Population: Adults aged 18 years or older with a diagnosis of stroke and resultant upper limb dysfunction.
- Intervention: VR-based therapy.
- Comparison: Conventional rehabilitation approaches.
- Outcome: Upper limb motor function, functional independence, quality of life, spasticity, and dexterity.

### Literature search and selection

#### Search methods

We searched PubMed, Scopus, IEEE, Web of Science, and Psycnet for randomized controlled trials (RCTs) published in English from inception to March 2023. The author M.S. developed search strategies for each database that combined terms for stroke, upper extremity impairments, VR, and study designs (full strategies in Additional File 1).

#### Study selection

The author (S.H.) executed the searches. Two authors (SH and MS) independently screened titles, abstracts, and full texts using the predefined inclusion criteria. Any conflicts or uncertainties regarding study inclusion or exclusion were resolved through discussion and consensus with the third author (M.GS.). To determine eligibility and eliminate duplicate entries, we employed EndNote and Rayyan [33] software applications.

#### Data extraction

A standardized, pre-designed data extraction form was utilized by two independent reviewers (SH and MS) to collect relevant data from each included study. The following categories of information were extracted: study identification details (authors, year, citation); country; VR type; intervention duration and frequency; outcome measures used; sample demographics including number of males, number of females, mean age overall and by study group; health status at admission; time since stroke onset; quantitative results for each study arm including means, standard deviations, event counts, p-values, effect sizes, and/or other statistical outputs as reported; advantages and disadvantages of VR identified; challenges and side effects documented; and any other results deemed relevant. Whenever accessible, outcome data from follow-up assessments were extracted, and the number of participants evaluated at this particular time point was reported. A comprehensive dataset, with detailed information, is available in Additional File 2.

### Study characteristics

#### Study design

We included RCTs with individual randomization. Eligible studies compared VR to conventional therapy or a control condition. Single-session interventions were excluded. We did not restrict studies based on VR intensity or duration provided it was more than one session.

#### Participants

Participants were adults aged 18 years or older with a clinical diagnosis of stroke based on neuroimaging or examination. Upper limb impairments stemming from the stroke were required for inclusion.

#### Interventions

The VR interventions met the definition of “an advanced human-computer interface that allows realistic user interaction and immersion within a computer-generated environment.” [16]. Eligible VR interventions encompassed various modalities, including non-immersive, semi-immersive, and fully immersive VR, which utilized either commercial gaming consoles or specialized programming to create interactive rehabilitation environments. Control conditions were structured to encompass conventional rehabilitation approaches or alternative interventions. The inclusion criteria for control groups were defined to encompass all interventions that were not classified as VR, whether they were full, semi, or non-immersive types. This approach aimed to ensure clarity and transparency in the delineation of control groups. Conventional rehabilitation approaches, which constituted the majority of control interventions, typically consisted of traditional physical therapy sessions. These sessions were tailored to focus on a spectrum of exercises and activities aimed at enhancing various aspects of motor function and daily living skills. Specifically, they encompassed range of motion exercises, strengthening exercises, functional training, and occupational therapy sessions targeting activities of daily living and fine motor skills. Furthermore, alternative interventions within the control groups comprised innovative approaches such as robotic-assisted therapy, virtual reality-based interventions, gaming rehabilitation platforms, and robotics utilizing screen displays. Robotic-assisted therapy employed devices such as robotic exoskeletons, robotic arms, or robotic gloves that utilized monitor screen displays to facilitate movement and deliver repetitive task-oriented training.

#### Outcomes

With the focus on upper limb rehabilitation, the primary outcomes were:

1. Motor function assessed by FMA, ARAT, WMFT, JTHFT, Grip strength, Manual Muscle Testing (MMT), and Passive Range of Motion (ROM).
2. Functional independence is measured by BI, Functional Independence Measure (FIM), or comparable tools.
3. Quality of life and impact using SIS.
4. Spasticity rated with MAS.
5. Functional use and dexterity determined by the Motor Activity Log (MAL), BBT, or analogous instruments.

#### Exclusion criteria

Studies were excluded from the review based on the following pre-specified criteria: interventions that did not target the upper limbs; utilization of nonrandomized, protocol or observational study designs; lack of open access to full-text publication; inclusion of neurological conditions such as Parkinson’s disease that mimic stroke; and absence of any form of VR within the therapeutic approach. Furthermore, studies solely employing robotic interventions without screen display were also excluded.

### Data analysis

#### Measures and analysis

The author (MS) systematically categorized outcome measures into five domains: motor function, functional independence assessment, quality of life and impact assessment, muscle spasticity assessment, and functional use and dexterity. Within each domain, the most frequently utilized measure underwent meta-analysis, with mean differences (MD) or standardized mean differences (SMD) calculated as appropriate. The selection between MD and SMD was contingent upon the variability and scale of the outcome measures within each domain. Specifically, SMD was employed in the meta-analysis for outcome measures that exhibited variability in their scale or measurement units, thereby allowing for the standardization of effect sizes across different instruments and assessment tools. Conversely, MD was utilized in the summary tables to present the absolute differences between intervention and control groups, thus offering a clear and interpretable measure of treatment effect magnitude. By incorporating both MD and SMD in our analysis and summary tables, our aim is to provide a comprehensive and informative synthesis of the evidence, ensuring clarity and interpretability. Additionally, less frequently used measures were summarized in the results without undergoing meta-analysis. The meta-analyses and creation of forest plots, summary of findings tables, and other graphical outputs were conducted using Review Manager 5 (RevMan 5) and GRADEpro software [34, 35].

#### Missing data

Some studies only reported pre-post intervention means without change scores or standard deviations. To enable meta-analysis, the following formulas were applied to estimate missing standard deviations for the change in means:

$$\eqalign{{\rm{Standard}}\,{\rm{Deviation}}\,\left( {\mathop {\rm{Y}}\limits^ - - \mathop {\rm{X}}\limits^ - } \right)\, = & \cr & \sqrt {{{{\rm{((}}{{\rm{n}}_1} - {\rm{1)*}}{{\mathop {\rm{X}}\limits^ - }^2}{\rm{)}} + \,{\rm{((}}{{\rm{n}}_2} - {\rm{1)*}}{{\mathop {\rm{Y}}\limits^ - }^2}{\rm{)}}} \over {{{\rm{n}}_1} + {{\rm{n}}_2} - 2}}} \cr}$$

Where $\stackrel{-}{\text{X}}$is the pre-intervention mean, $\stackrel{-}{\text{Y}}$is the post-intervention mean, ${\text{n}}_{1}$ is the pre-intervention sample, and ${\text{n}}_{2}$ is the post-intervention sample.

#### Risk of bias assessment

The Cochrane Risk of Bias 2 tool [36] was applied independently by two authors (SH and MS) to appraise sources of bias in randomization, deviations from intended interventions, missing data, outcome measurement, and selection of the reported result. Items were judged as low, high, or some concern for risk of bias. The GRADE approach informed the interpretation of findings [37]. The GradePro GDT generated Summary of Findings tables summarizing judgment of the overall quality of evidence for each outcome [34].

#### Heterogeneity and reporting bias assessment

A random effects model synthesized the results. Heterogeneity was visually inspected and quantified using the I^2^ statistic, with lower than 25% representing mild heterogeneity, 25–50% moderate, 50–75% substantial, and over 75% considerable heterogeneity. We evaluated outcome reporting bias by comparing methods and results. Funnel plots were examined. Comprehensive funnel plots for both random effects and fixed effect models are available in Additional File 3.

#### Data synthesis

Random effects meta-analysis was conducted in RevMan 5 with 95% confidence intervals. SMD pooled outcomes across different instruments.

#### Subgroup analysis

Predefined subgroup analyses were conducted based on key parameters, including patients’ mean age, stroke severity, time elapsed since the stroke event, intervention dosage, and the type of VR utilized. Insufficient data prevented all proposed analyses. Subgroup comparisons were performed for intervention duration, time since stroke onset, customized versus commercial VR, and upper limb impairment severity when feasible.

#### Sensitivity analysis

Sensitivity analyses were conducted to evaluate the influence of study quality and the choice between fixed and random effects models on the overall outcomes. Given the diverse range of interventions, outcome measures, and participant characteristics across the included studies, these analyses were integral to assessing the robustness of our findings and ensuring the reliability of our conclusion.

## Results

### Characteristics of studies

Of the 11,834 studies screened, 55 RCTs met the predefined inclusion criteria and were incorporated into this systematic review. The included trials had sample sizes ranging from eight to 139 participants per study arm, totaling 2,142 participants overall. Participants were adults aged 18 years and older with confirmed diagnoses of ischemic or hemorrhagic stroke resulting in upper limb motor deficits. Most studies had predominantly male samples, with few examining sex-based differences. Participants spanned the continuum of stroke recovery time points, with 24 trials examining subacute stroke populations within six months of onset, 28 trials focusing on chronic stroke cohorts over six months post-cerebrovascular accident, and four trials not reporting time since onset. Intervention periods ranged from two days to six months. The mean age of participants across 52 studies was 59.5 years, while three studies did not report mean age. The trials originated from 15 countries, primarily China, South Korea, and the United States.

**Fig. 2:**
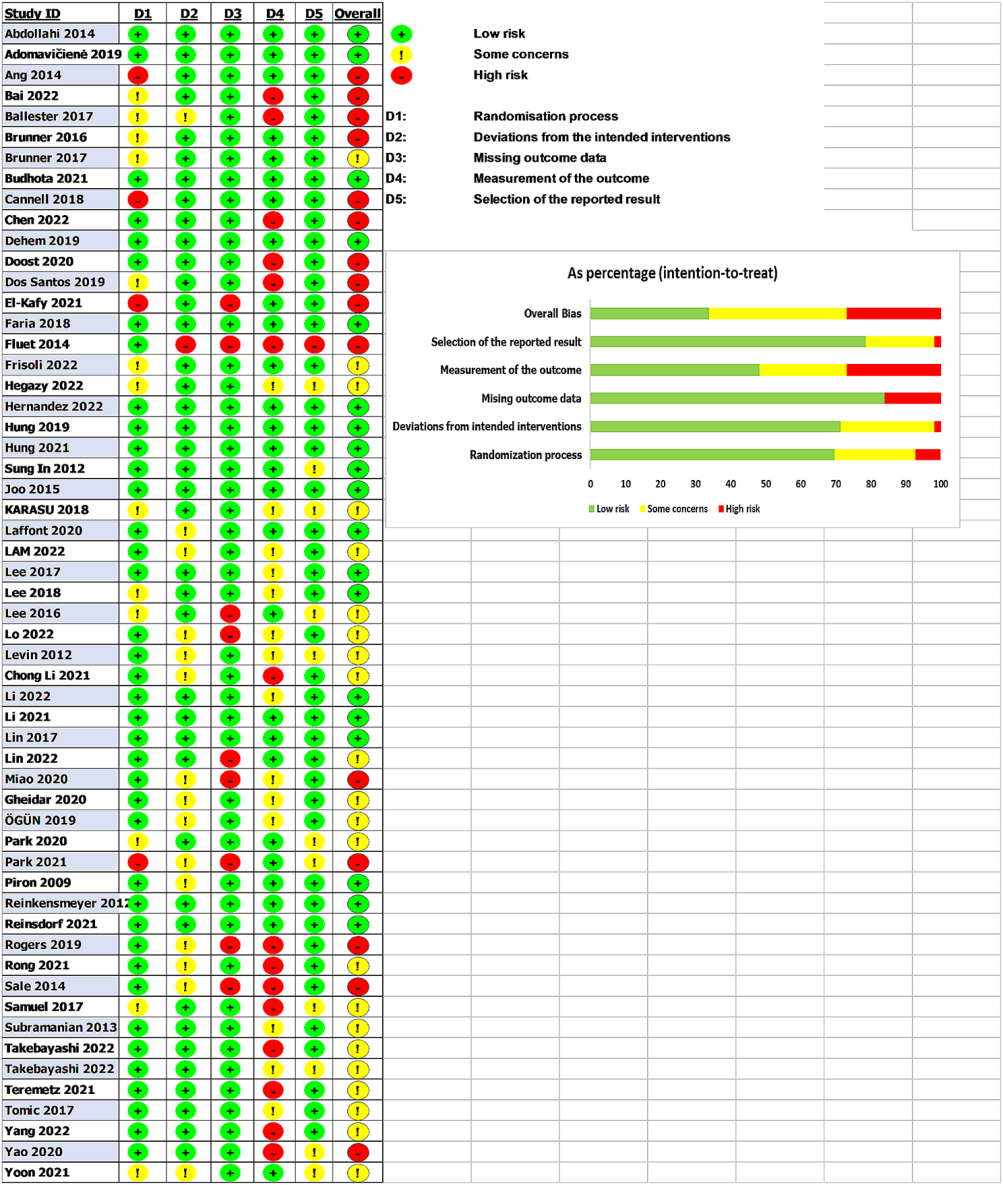
Assessment of study quality: Cochrane’s risk of bias 2 analysis

The risk of bias appraisal using the Cochrane Risk of Bias 2 tool revealed a low risk of bias in the majority of studies for the randomization process (69.6%), deviations from intended interventions (71.4%), and missing outcome data (83.9%). However, nearly half of the studies demonstrated some concerns related to the measurement of the outcome (48.2%), primarily due to the lack of blinding of outcome assessors. The risk of bias due to measurement was low in 78.6% of studies. The domain with the greatest risk of bias was the selection of the reported result, with 26.8% of studies rated as high risk, 39.3% as some concerns, and only 33.9% as low risk. This indicates selective outcome reporting may be an issue in some included studies (Fig. 2).

Various virtual reality modalities were evaluated, categorized by immersion depth as fully immersive, semi-immersive, and non-immersive platforms using customized VR programming or commercial gaming devices. Control conditions consisted of conventional occupational or physical therapy matched in duration and intensity to the VR protocols. Outcomes included validated assessments of upper extremity motor function, activities of daily living, dexterity, spasticity, quality of life, and patient-reported functional use. The Fugl-Meyer Assessment was the most commonly reported measure, utilized in 34 trials, followed by the Barthel Index and Box and Blocks Test in 13 trials each. While the randomized controlled trial design demonstrates rigor, limitations existed including small sample sizes, lack of blinding, and short intervention durations, restricting the detection of potential VR benefits.

### Motor function

The capacity for volitional governance of muscular movement and coordination also referred to as motor function, is often impaired following a cerebrovascular accident (CVA or stroke). Remediation of motor deficits holds promise for ameliorating restrictions in patient ambulation and executing activities of daily living that often accompany hemiparesis or hemiplegia after CVA [10]. Several psychometric instruments have been designed and validated to quantify and characterize upper extremity motor function in stroke patients, including FMA, ARAT, WMFT, Jebsen-Taylor Hand Function Test (JTHFT), and Grip strength. These evaluation tools provide clinicians and researchers with robust means to assess motor recovery and response to therapeutic interventions.

#### Fugl-Meyer assessment

**Fig. 3:**
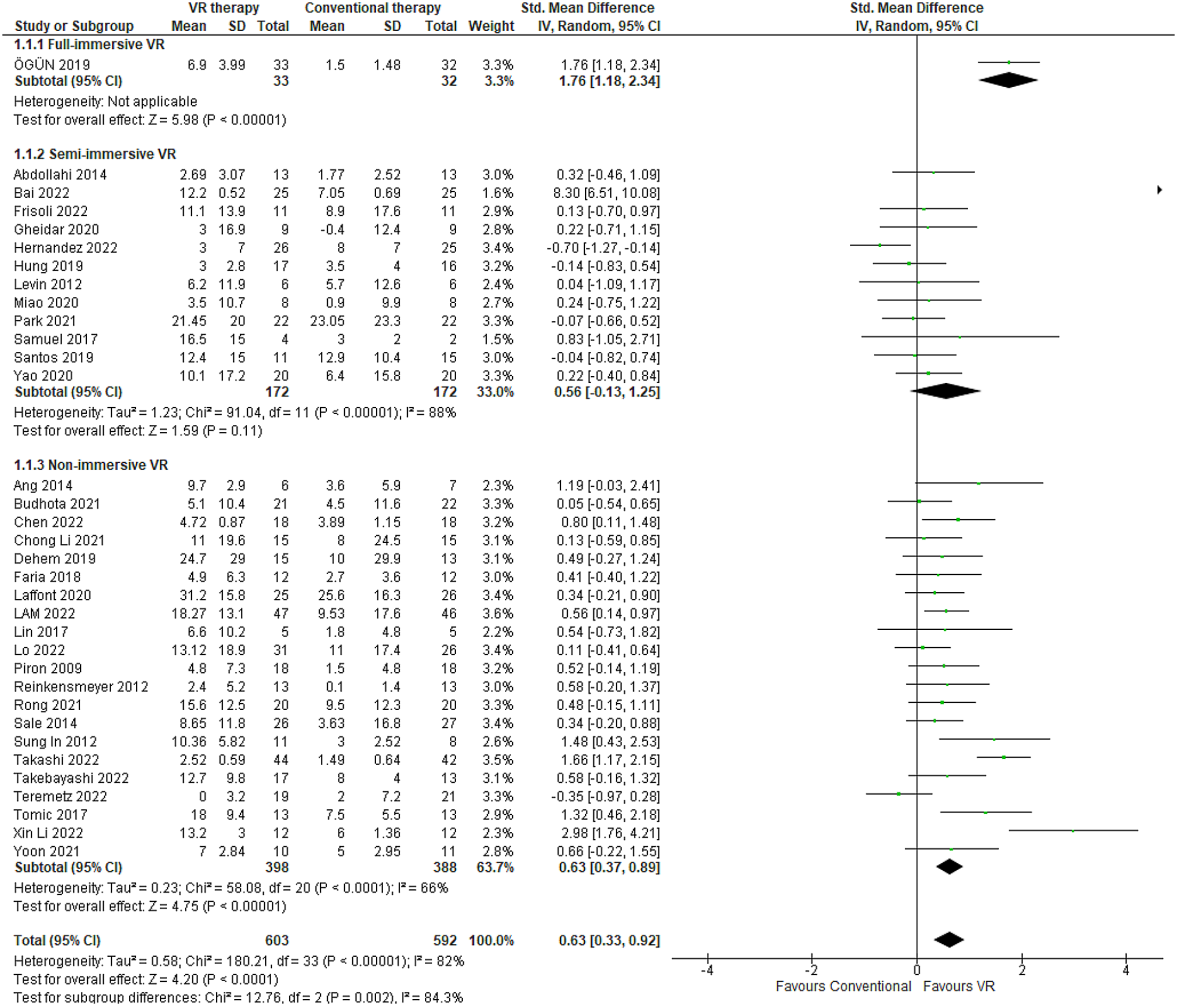
Meta-analysis forest plots: Comparing FMA improvement in VR-based and conventional therapy

FMA is a validated, psychometrically robust instrument for gauging upper extremity motor impairment in post-stroke patients [6]. FMA scores have demonstrated responsivity to changes in neuromuscular function. The analysis of 34 RCTs encompassing 1196 cases revealed that VR-based therapies improved upper limb motor function after stroke to a significantly greater degree than conventional occupational therapy regimens, based on FMA score differences (Table 1). The pooled effect size across studies was moderately large (SMD 0.63, 95% CI 0.33–0.92) with low between-study heterogeneity (I^2^ = 82%). Post-intervention FMA assessment time-points ranged from two weeks [38] to six months [39–41]. The minimum and maximum mean FMA score changes reported were − 0.4 [42] and 31.2 [41] points, respectively (Fig. 3).

Further subgroup analysis by VR immersion level showed differential efficacy, with fully immersive VR conferring the greatest motor gains. Full-immersive VR yielded a mean FMA improvement of 5.4 points (95% CI 5.02–5.77) over conventional therapy [38] (Table 1). Semi-immersive VR showed a smaller mean FMA change of 2.03 points (95% CI 1.8–2.25; 12 studies) [42–53] (Table 2). Non-immersive VR exhibited an intermediate benefit, with a mean FMA increase of 4.58 points (95% CI 4.48–4.67; 21 studies) [39–41, 54–71] (Table 3). The sensitivity analyses indicated minimal discrepancies in FMA scores when comparing random-effects and fixed-effect models (0.63 [95% CI 0.33–0.92] in random-effects compared to 0.50 [95% CI 0.38–0.62] in fixed-effects).

**Table 1:** Summary of findings for full-immersive virtual reality

Summary of findings:					
Full-immersive virtual reality compared to conventional therapy for upper limb rehabilitation after stroke					
**Setting**: The full immersive virtual reality interventions were delivered in rehabilitation hospitals and research centers using specialized VR headsets and equipment. Patients interact with the virtual environment through movements of the affected upper limb.					
**Outcomes**		**Anticipated absolute effects** ^*^ **(95% CI)**		**№ of participants** **(studies)**	**Certainty of the evidence** **(GRADE)**
		**Improvement with conventional therapy**	**Improvement with fully immersive virtual reality systems**		
**Motor Function**	**FMA** Scale from: 1.5 to 6.9 follow-up: six weeks	The mean FMA improvement was **1.5** points	MD **5.4 points higher** (5.02 higher to 5.77 higher)	65 (1 RCT)	⨁⨁⨁◯ Moderate ^a^
	**ARAT** Scale from: 1.25 to 8.33 follow-up: range 1 week to six weeks	The mean ARAT improvement was **1.25** points	MD **7.08 points higher** (6.67 higher to 7.49 higher)	65 (1 RCT)	⨁⨁⨁◯ Moderate ^a^
	**Grip strength** Scale from: 3 to 11.1 follow-up: six weeks	The mean grip strength improvement was **3** points	MD **8.1 points higher** (5.76 higher to 10.43 higher)	20 (1 RCT)	⨁⨁⨁◯ Moderate ^a^
**Functional Independence Assessment**	**FIM** Scale from: 0.71 to 4.78 follow-up: range 1 week to six weeks	The mean FIM improvement was **0.71** points	MD **4.07 points higher** (3.54 higher to 4.59 higher)	65 (1 RCT)	⨁⨁⨁◯ Moderate ^a^
	**PASS-BADL** Scale from: 0.03 to 0.38 follow-up: range 1 week to six weeks	The mean PASS-BADL improvement was **0.03** points	MD **0.35 points higher** (0.3 higher to 0.39 higher)	65 (1 RCT)	⨁⨁⨁◯ Moderate ^a^
	**PASS-IADL** Scale from: 0.03 to 0.39 follow-up: range 1 week to six weeks	The mean PASS-IADL improvement was **0.03** points	MD **0.36 points higher** (0.32 higher to 0.39 higher)	65 (1 RCT)	⨁⨁⨁◯ Moderate ^a^
**Functional Use and dexterity**	**UEFI** Scale from: 9.3 to 12.6 follow-up: range 1 week to six weeks	The mean UEFI improvement was **9.3** points	MD **3.3 points higher** (2 lower to 8.7 higher)	20 (1 RCT)	⨁⨁⨁◯ Moderate ^a^

a. The study had unclear or high risk of bias in certain domains

**Table 2:** Summary of findings for Semi-immersive virtual reality

Summary of findings:					
Semi-immersive virtual reality compared to conventional therapy for upper limb rehabilitation after stroke					
**Setting**: The semi-immersive virtual reality interventions were delivered in rehabilitation hospitals, clinics, and some home settings using video capture or robotics to track patient movements and interact with games. The systems provided visual feedback on a display.					
**Outcomes**		**Anticipated absolute effects** ^*^ **(95% CI)**		**№ of participants** **(studies)**	**Certainty of the evidence** **(GRADE)**
		**Improvement with conventional therapy**	**Improvement with semi-immersive VR**		
**Motor Function**	**FMA** Scale from: -0.4 to 23.05 follow-up: range two weeks to 12 weeks	The mean FMA Improvement was **6.73** points	MD **2.03 points higher** (1.8 higher to 2.25 higher)	344 (12 RCTs)	⨁◯◯◯ Very Low ^a, b^
	**ARAT** Scale from: -5.23 to 17.3 follow-up: range two weeks to 3 months	The mean ARAT Improvement was **6.52** points	MD **4.83 points higher** (4.53 higher to 5.13 higher)	256 (5 RCTs)	⨁◯◯◯ Very low ^a, b^
	**WMFT** Scale from: -6.64 to 8.7 follow-up: range from two weeks to 3 months	The mean WMFT Improvement was **0.47** points	MD **3.48 points higher** (3.16 higher to 3.79 higher)	98 (4 RCTs)	⨁◯◯◯ Very low ^a, b^
	**JTHFT** Scale from: 12.36 to 38.4 follow-up: range two weeks to six weeks	The mean JTHFT Improvement was **16.27** seconds	MD **11.94 s lower** (10.55 lower to 13.32 lower)	102 (3 RCTs)	⨁◯◯◯ Very low ^a, c^
	**Grip Strength** Scale from: 0.21 to 12.82 follow-up: range four weeks to 3 months	The mean grip Strength Improvement was **2.65** Kg	MD **1.7 Kg higher** (1.3 higher to 2.06 higher)	137 (4 RCTs)	⨁⨁◯◯ Low ^a, d^
**Functional Independence Assessment**	**FIM** Scale from: 13.4 to 17.7 follow-up: 3 month	The mean FIM improvement was **16.4** points	MD **0.5 points higher** (0.8 lower to 1.9 higher)	102 (1 RCT)	⨁⨁⨁◯ Moderate ^a^
	**BI** Scale from: -3.3 to 31.68 follow-up: range two weeks to 12 weeks	The mean BI Improvement was **9.14** points	MD **4.6 points higher** (4.09 higher to 5.09 higher)	169 (4 RCTs)	⨁◯◯◯ Very low ^a, b^
**Muscle Spasticity Assessment**	**MAS** Scale from: 0 to 1.5. follow-up: range six weeks to 12 weeks	The mean MAS Improvement was **0.7** points	MD **0.08 points higher** (0.8 lower to 0.98 higher)	57 (2 RCTs)	⨁⨁⨁◯ Moderate ^a^
**Functional Use and dexterity**	**MAL-AOU** Scale from: -0.1 to 0.39 follow-up: range 3 weeks to 12 weeks	The mean MAL-AOU Improvement was **1.02** points	MD **0.18 points higher** (0.04 higher to 0.31 higher)	63 (3 RCTs)	⨁⨁⨁◯ Moderate ^a^
	**BBT** Scale from: 0 to 34.5 follow-up: range 3 weeks to 10 weeks	The mean BBT Improvement was **10.2** blocks	MD **4.3 blocks higher** (3.98 higher to 4.57 higher)	318 (7 RCTs)	⨁◯◯◯ Very low ^a, b^

a. Some studies had unclear or high risk of bias in certain domains

b. I^2^ statistic of > 75% reflects an ‘’extremely serious’ level of heterogeneity

c. I^2^ statistic of 50–75% reflects a ‘very serious’’ level of heterogeneity

d. I^2^ statistic of 25–50% reflects a serious’’ level of heterogeneity

**Table 3:** Summary of findings for non-immersive virtual reality

Summary of findings:					
Non-immersive virtual reality compared to conventional therapy for upper limb rehabilitation after stroke					
**Setting**: Non-immersive VR uses conventional computers and monitors without specialized equipment to display simulated environments for motor and cognitive therapy. Patients interact using a mouse or touchscreen and perform repetitive upper extremity tasks with visual/auditory feedback.					
Outcomes		**Anticipated absolute effects** ^*^ **(95% CI)**		№ of participants (studies)	Certainty of the evidence (GRADE)
		**Improvement with conventional therapy**	**Improvement with non-immersive VR**		
**Motor Function**	**FMA** Scale from: 0 to 31.2 follow-up: range two weeks to six months	The mean FMA improvement was **6.11** points	MD **4.58 points higher** (4.48 higher to 4.67 higher)	786 (21 RCTs)	⨁⨁◯◯ Low ^a, b^
	**ARAT** Scale from: 1 to 17.23 follow-up: range two weeks to 24 weeks	The mean ARAT improvement was **4.99** points	MD **2.52 points higher** (1.83 higher to 2.66 higher)	242 (5 RCTs)	⨁⨁⨁◯ Moderate ^a^
	**WMFT** Scale from: 0.29 to 22.6 follow-up: range two weeks to six months	The mean WMFT improvement was **4.62** points	MD **5.07 points higher** (4.83 higher to 5.3 higher)	249 (8 RCTs)	⨁⨁◯◯ Low ^a, c^
	**Grip strength** Scale from: 0.51 to 5.64 follow-up: range four weeks to 24 weeks	The mean grip strength improvement was **1.71** Kg	MD **0.89 Kg higher** (0.74 higher to 1.03 higher)	202 (4 RCTs)	⨁⨁⨁◯ Moderate ^a^
**Functional Independence Assessment**	**BI** Scale from: 0 to 42.7 follow-up: range two weeks to six months	The mean BI improvement was **13.06** points	MD **2.88 points higher** (2.43 higher to 3.33 higher)	323 (9 RCTs)	⨁⨁⨁◯ Moderate ^a^
	**FIM** Scale from: 12.6 to 15.39 follow-up: four weeks	The mean FIM improvement was **12.6** points	MD **2.79 points higher** (2.3 higher to 3.2 higher)	40 (1 RCTs)	⨁⨁⨁◯ Moderate ^a^
**Quality of Life and Impact Assessment**	**SIS** Scale from: -0.7 to 23.1 follow-up: range four weeks to six months	The mean SIS improvement was **2** points	MD **9.2 points higher** (7.51 higher to 10.89 higher)	68 (2 RCTs)	⨁◯◯◯ Very low ^a, d^
**Muscle Spasticity Assessment**	**MAS** Scale from: -0.5 to 0.18 follow-up: range four weeks to 1 month	The mean MAS improvement was **− 0.27** points	MD **0.27 points higher** (0.21 higher to 0.33 higher)	132 (4 RCTs)	⨁⨁⨁◯ Moderate ^a^
**Functional Use and dexterity**	**MAL-AOU** Scale from: -0.05 to 2.2 follow-up: range four weeks to six months	The mean MAL-AOU improvement was **0.41** points	MD **0.51 points higher** (0.48 higher to 0.54 higher)	203 (4 RCTs)	⨁◯◯◯ Very low ^a, d^
	**BBT** Scale from: 0.2 to 23.8 follow-up: range 2 days to six months	The mean BBT improvement was **3.92** blocks	MD **3.05 blocks higher** (2.74 higher to 3.38 higher)	213 (6 RCTs)	⨁⨁◯◯ Low ^a, b^

a. Some studies had unclear or high risk of bias in certain domains

b. I^2^ statistic of 50–75% reflects a ‘very serious’’ level of heterogeneity

c. I^2^ statistic of 25–50% reflects a serious’’ level of heterogeneity

d. I^2^ statistic of > 75% reflects an ‘’extremely serious’ level of heterogeneity

#### Action research arm test

**Fig. 4:**
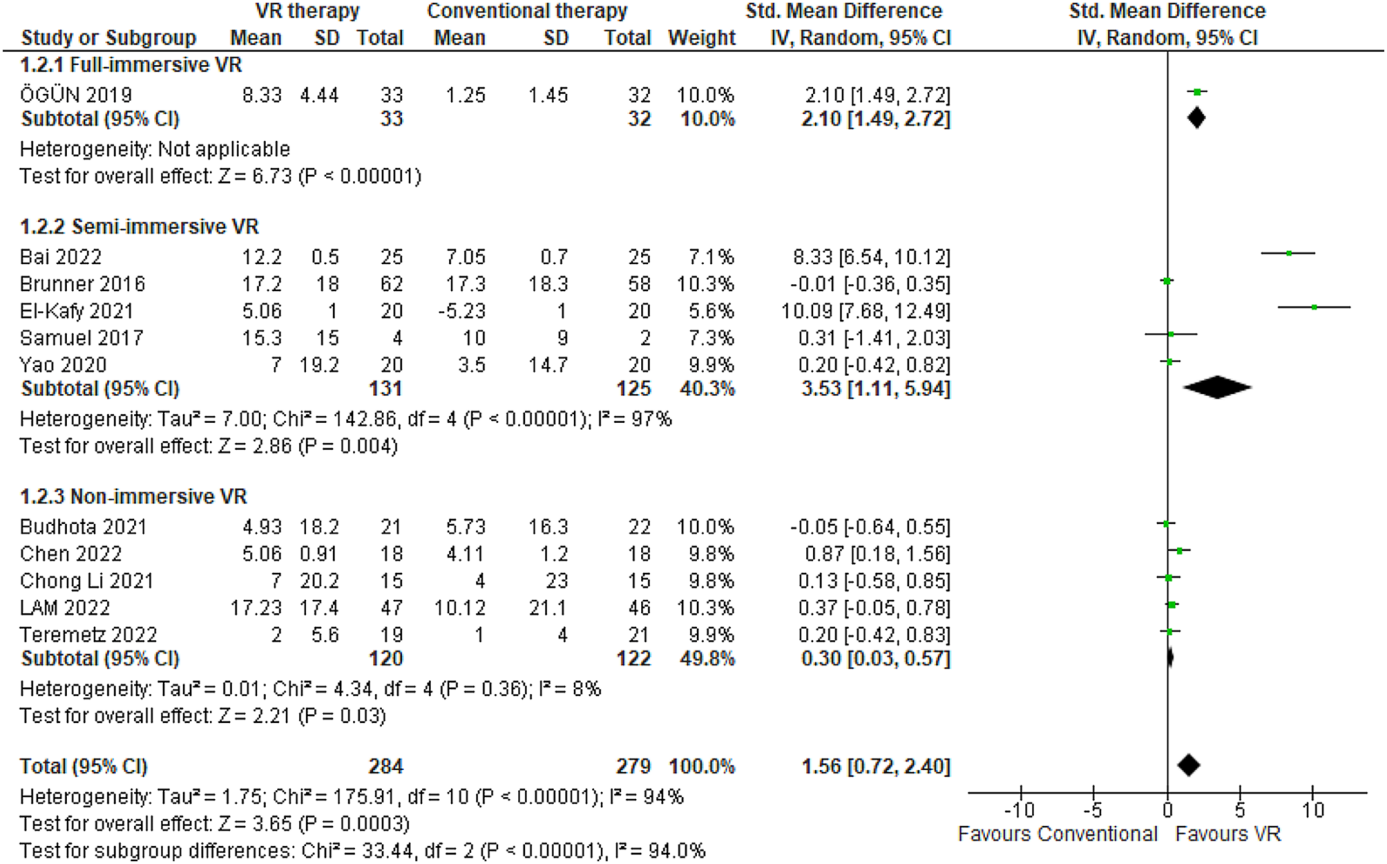
Meta-analysis forest plots: Comparing ARAT improvement in VR-based and conventional therapy

ARAT is an assessment tool that examines upper limb motor function related to manipulative dexterity and application of grip force, which are requisite for executing activities of daily living [72]. It quantifies proximal arm gross motor function and manual object manipulation competencies crucial for functional independence. This meta-analysis synthesized data from 11 RCTs encompassing 563 stroke patients to evaluate the efficacy of VR-based therapies versus conventional occupational therapy for improving post-stroke upper extremity motor function as measured by the ARAT [38, 44, 51, 52, 55, 56, 59, 60, 69, 73, 74]. Considerable between-study heterogeneity was reported (I^2^ = 94%), yet the pooled effect size significantly favored VR intervention over conventional therapy (SMD 1.56, 95% CI 0.72–2.4) (Fig. 4). Follow-up ARAT assessments were conducted from two [38, 51, 52, 56, 60] to 24 weeks [55] post-intervention across studies. The range of mean ARAT score differences between VR and conventional therapy groups was − 5.23 [74] to 17.23 [59] points. Analysis by immersion level showed VR approaches had varying efficacy. Full-immersive VR exhibited the greatest gains with a mean ARAT improvement of 7.08 points (95% CI 6.67–7.49) over conventional therapy [38] (Table 1). Semi-immersive VR showed a lower but still positive effect, with a mean ARAT increase of 4.83 points (95% CI 4.53 to 5.13) compared to full-immersion [44, 51, 52, 73, 74] (Table 2). Non-immersive VR had the smallest impact on ARAT scores, conferring only a 2.52-point mean advantage (95% CI 1.83 to 2.66) [55, 56, 59, 60, 69] (Table 3). The sensitivity analyses revealed significant disparities in ARAT scores when contrasting random-effects and fixed-effect models (1.56 [95% CI 0.72–2.40] in random-effects versus 0.52 [95% CI 0.33–0.70] in fixed-effects).

Subgroup analyses revealed certain factors impacting VR’s efficacy on ARAT outcomes. Shorter VR interventions of 2–4 weeks (*n* = 7 trials) [38, 51, 52, 56, 60, 69, 75] resulted in lower ARAT gains (MD 3 points) compared to longer interventions of 6–12 weeks (*n* = 4 trials), which improved ARAT scores by 5.4 points on average [44, 55, 59, 74]. Younger participants under age 60 (*n* = 5 trials) had lower ARAT enhancements (3.3 points) [44, 55, 56, 69, 74] than older participants over age 60 (*n* = 6 trials) who improved by 4.3 points [38, 51, 52, 59, 60, 75]. Patients in subacute phases within six months of stroke onset (*n* = 6 trials) exhibited lower ARAT gains (3.6 points) [44, 51, 56, 59, 60, 75] compared to chronic stroke patients (*n* = 5 trials) who improved by 4.2 points [38, 52, 55, 69, 74]. Finally, trials using robotic devices (*n* = 3) showed larger ARAT enhancements (4.7 points) than trials using video games [59, 60, 69] or VR [38, 44, 51, 52, 56, 75] approaches, which increased ARAT by 3.7 and 3.6 points respectively.

#### Wolf motor function test

**Fig. 5:**
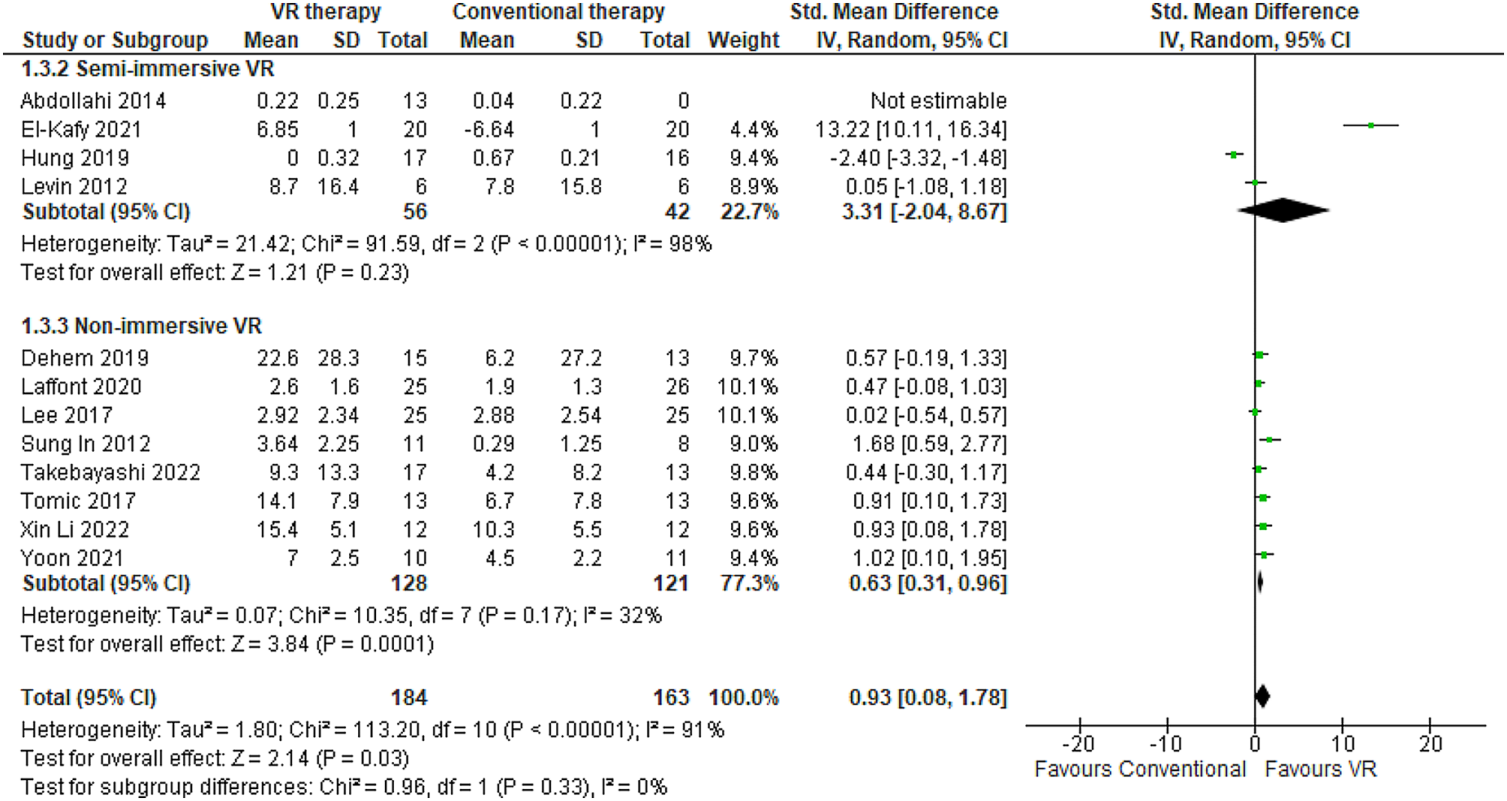
Meta-analysis forest plots: Comparing WMFT improvement in VR-based and conventional therapy

WMFT comprises standardized tasks evaluating critical manipulation skills, manual dexterity, and fine motor coordination such as lifting and transporting small items, stacking checkers, and controlled pouring of liquid [76]. It provides a timed assessment of proximal and distal arm function involved in executing activities of daily living. This meta-analysis synthesized data from 12 RCTs totaling 347 patients to compare the efficacy of VR therapy versus conventional occupational therapy on post-stroke motor function using the WMFT [40, 41, 43, 47, 48, 58, 61, 67, 70, 71, 74, 77]. Substantial heterogeneity was observed across studies (I^2^ = 91%). Post-intervention WMFT assessments occurred two weeks [43, 77] to six months [40, 41] after completion of therapy. The pooled effect size significantly favored VR intervention (SMD 0.93, 95% CI: 0.08–1.78) (Fig. 5). Differences in mean WMFT scores between VR and conventional therapy groups ranged from − 6.64 [74] to 22.6 points [40]. Stratification by immersion technique revealed no studies utilizing fully immersive VR assessed the WMFT. Non-immersive VR demonstrated the greatest gains with a mean WMFT score increase of 5.07 points over conventional therapy [40, 41, 58, 61, 68, 70, 71, 77] (Table 3). Semi-immersive VR also showed benefits but to a lesser degree (MD: 3.48 points) [43, 47, 48, 74] (Table 2). The sensitivity analyses disclosed noteworthy variations in WMFT scores when comparing random-effects and fixed-effect models (0.93 [95% CI 0.08–1.78] in random-effects versus 0.42 [95% CI 0.18–0.67] in fixed-effects).

Shorter VR regimens of 2–4 weeks (*n* = 7 trials) conferred inferior WMFT improvements (MD 2.9 points) [43, 48, 58, 61, 70, 71, 77] compared to longer protocols of 6–12 weeks (*n* = 5 trials), which reduced WMFT time by seven points [40, 41, 47, 68, 74]. Investigations in subacute stroke populations under six months (*n* = 8 trials) demonstrated greater declines in WMFT (5.07 points) [40, 41, 58, 61, 68, 70, 71, 77] than evaluations in chronic cohorts over six months (*n* = 4) where WMFT increased by just 3.5 points [43, 47, 48, 74]. Older participants above age 60 (*n* = 4 trials) exhibited superior WMFT reductions (6.8 points) [40, 58, 68, 71] than younger subjects above age 60 (*n* = 8), whose WMFT declined by 3.4 points [41, 43, 45, 47, 48, 61, 70, 74, 77]. Finally, trials employing robotic devices (*n* = 8) demonstrated greater WMFT improvements (6.3 points) [40, 43, 61, 68, 70, 71, 74, 77, 78] than those utilizing video games or computerized modalities (*n* = 4), which decreased WMFT by 0.3 and 3.4 points respectively [41, 47, 58, 75]. Reported adverse events like fatigue or pain were minimal [41, 47].

#### Grip strength

**Fig. 6:**
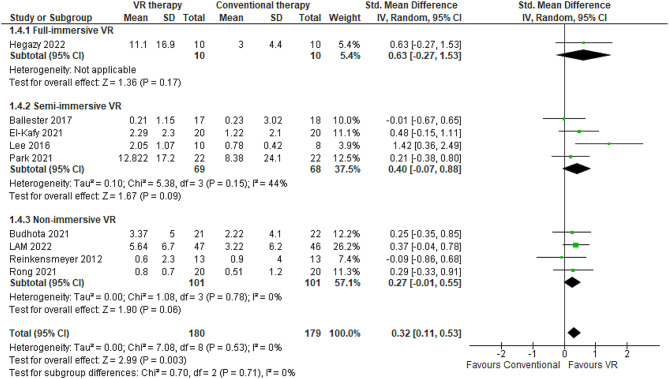
Meta-analysis forest plots: Comparing grip strength improvement in VR-based and conventional therapy

Grip strength, measured via dynamometry, provides a quantification of the maximum volitional isometric force generated by the forearm flexors and extensors and serves as an index of global arm strength. This meta-analysis synthesized data from nine RCTs involving 359 stroke patients that utilized grip dynamometry to compare the efficacy of VR-based rehabilitation compared to conventional occupational therapy on improving post-stroke upper limb strength [50, 55, 59, 64, 65, 74, 79–81]. There was no statistical heterogeneity across studies (I^2^ = 0%). The pooled effect size significantly favored VR, albeit small in magnitude (SMD 0.32, 95% CI 0.11–0.53) (Fig. 6). Follow-up assessments were conducted from one [79] to 24 weeks [55] after completing the interventions. The maximum difference in mean grip strength between VR and conventional therapy groups was 12.82 kg [50], while the minimum difference was 0.21 kg [80]. Analysis by immersion level revealed VR modalities conferred varying degrees of benefit. Fully immersive VR appeared more advantageous, correlating with a mean grip strength increase of 8.1 kg (95% CI 5.76–10.43) than conventional therapy [79] (Table 1). Semi-immersive VR showed more moderate gains of 1.7 kg (95% CI 1.3–2.06) compared to full immersion [50, 74, 80, 81] (Table 2). Non-immersive VR had the smallest effect on grip strength, conferring only a 0.89 kg advantage on average (95% CI 0.74–1.03) [55, 59, 65, 82] (Table 3). The sensitivity analyses revealed no differences in Grip strength scores when comparing random-effects and fixed-effect models.

Analyses revealed interventions using the VR system used a game called “Super Punch” along with VR goggles and controllers elicited the largest gains improvement in EG compared to CG in grip strength, averaging 8.1 kg [79]. This significantly exceeded more modest gains of 0.68 kg with robotic devices and 0.6 kg with alternate controllers [55, 64, 65, 74]. Younger cohorts with a mean age of 55 years exhibited greater enhancements, improving by 1.8 kg [55, 74, 79] more than older subgroups averaging 64 years [50, 59, 64, 80, 81]. Rehabilitation regimens lower than four weeks were associated with substantially higher effects, boosting grip strength by 3.4 kg [50, 65, 79] than just 0.9 kg for longer 6–24-week protocols [55, 59, 74, 80, 81, 83].

#### Jebsen Taylor hand function test

**Fig. 7:**
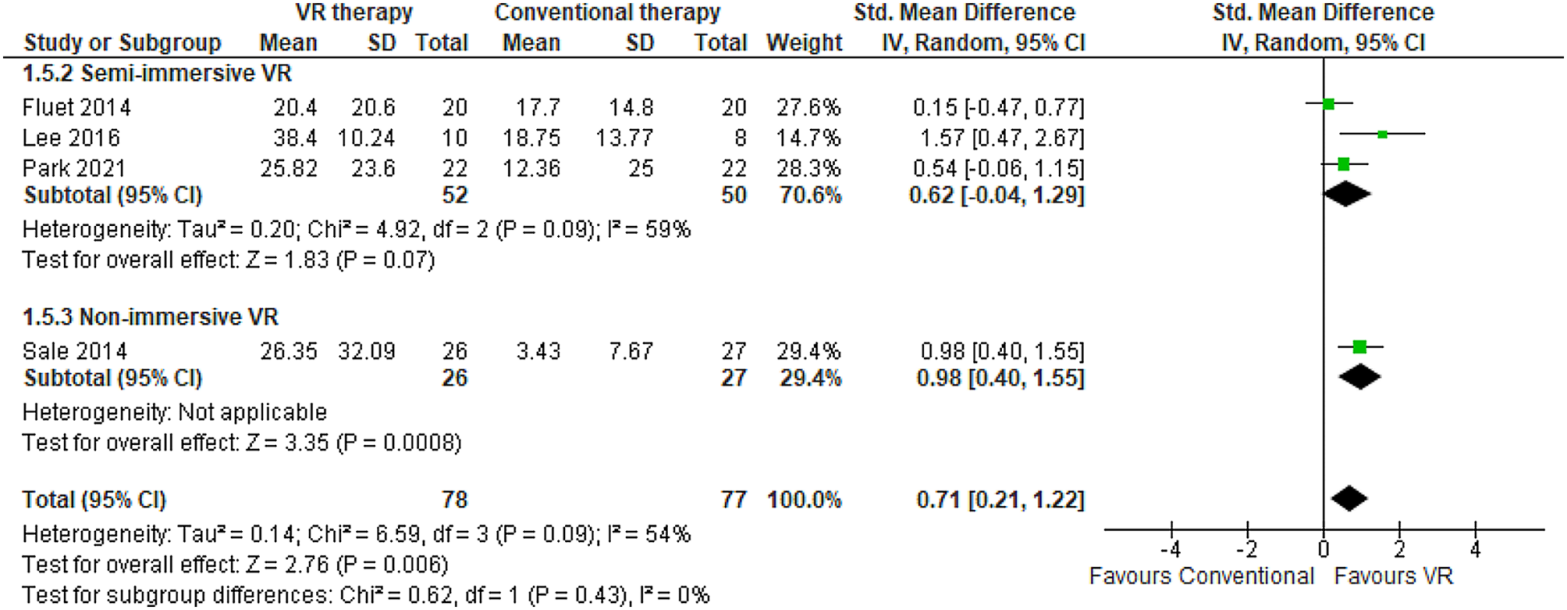
Meta-analysis forest plots: Comparing JTHFT improvement in VR-based and conventional therapy

JTHFT provides a timed assessment of multiple facets of manual dexterity through seven subtests involving simulated activities of daily living including written expression, page-turning, small object manipulation, simulated feeding, stacking, and lifting tasks of graded weights and sizes. It serves as a psychometrically robust tool for quantifying various components of fine motor control. Three RCTs comprising 155 stroke patients compared semi-immersive VR rehabilitation to conventional occupational therapy for improving post-stroke upper extremity function using the JTHFT [50, 81, 84]. Considerable between-study heterogeneity was present (I^2^ = 54%), however, the pooled effect size significantly favored VR (SMD 0.71, 95% CI 0.21–1.22) (Fig. 7). Follow-up evaluations occurred two [77, 84] to six weeks [66] after treatment completion. The range of JTHFT mean time score differences reported between VR and conventional therapy groups was 12.36 [50] to 38.4 s [81], with faster times indicating better performance. This meta-analysis revealed VR rehabilitation elicited significantly quicker JTHFT completion times versus conventional therapy (16.27 s faster on average), with a mean time reduction of 11.94 s (95% CI 10.55 to 13.32 s) (Table 2). No studies employing fully immersive VR utilized the JTHFT, and only one study used the JTHFT in the context of non-immersive VR [66] (Table 3). The sensitivity analyses suggested limited differences in JTHFT scores when comparing random-effects and fixed-effect models (0.71 [95% CI 0.21–1.22] in random-effects versus 0.67 [95% CI 0.34-1.00] in fixed-effects).

Subgroup analyses revealed the MIT-MANUS-InMotion2 robotic device improved JTHF by 22.9 points in the shoulder and elbow in EG compared to CG (26.3 vs. 3.43 points, respectively) [66]. adjustable sliding rail handles permitting limited 0–45-degree movements conferred more modest benefits that resulted in larger JTHF enhancements of 38.4 points compared to 18.7 points with conventional occupational therapy alone [81]. Rehabilitation protocols exceeding four weeks in duration did carry markedly greater effect sizes [66, 81] compared to briefer interventions (21.3 vs. 8.08 points, respectively) [50, 84].

### Functional independence assessment

Functional independence assessments (FIAs) appraise a patient’s capacity to autonomously execute activities of instrumental daily living (IADLs) such as bathing, dressing, grooming, toileting, transferring, and ambulation. Clinicians frequently employ FIAs to determine the level of assistance required for stroke survivors to maintain independence and to formulate customized rehabilitation regimens aimed at enhancing functional competencies [85, 86]. This meta-analysis identified several standardized FIA instruments, including BI, the Functional Independence Measure (FIM), and Postural Assessment Scale for Stroke evaluating basic ADLs (PASS-BADL), and the Postural Assessment Scale for Stroke assessing IADLs (PASS-IADL).

#### Bartal index

**Fig. 8:**
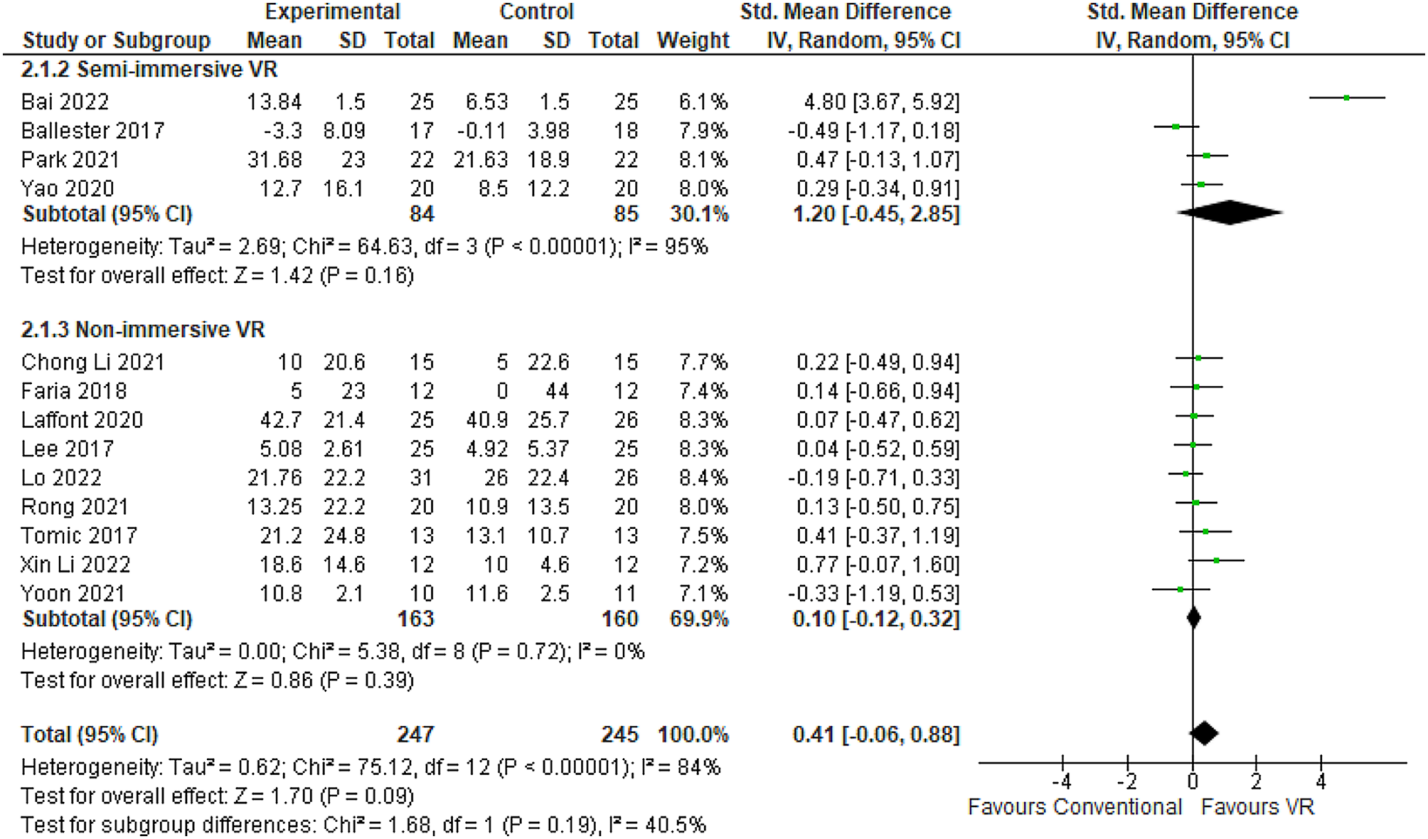
Meta-analysis forest plots: Comparing BI improvement in VR-based and conventional therapy

BI is a validated tool for appraising independence across core ADLs including feeding, bathing, grooming, dressing, toileting, transferring, ambulation, and bowel and bladder control. It provides an ordinal rating of the amount of assistance, in terms of physical support and time, required to execute various mobility and self-care tasks. Thirteen RCTs comprising 492 patients compared VR rehabilitation to conventional occupational therapy for enhancing post-stroke upper limb function using the BI as an evaluative outcome [39, 41, 44, 52, 57, 61, 65, 70, 71, 74, 77, 80, 87]. Considerable between-study heterogeneity was present (I^2^ = 84%). Nevertheless, the meta-analysis revealed a pooled effect size favoring VR intervention (SMD 0.41, 95% CI: -0.06-0.88) (Fig. 8). Follow-up BI assessments were conducted two weeks [52, 60, 77] to six months [39, 41] after stroke. Reported mean BI score differences varied widely from − 3.3 [80] to 42.7 points [41] across studies. Analysis by immersion technique showed rehabilitation efficacy increased with greater immersion levels. Specifically, semi-immersive VR correlated with a 4.6-point higher BI score on average compared to conventional therapy (95% CI: 4.09–5.09 points higher) [44, 50, 52, 80] (Table 2). Non-immersive VR conferred a more modest 2.88-point BI advantage [39, 41, 57, 60, 61, 65, 70, 71, 77] (Table 3). The sensitivity analyses revealed significant differences in BI scores when contrasting random-effects and fixed-effect models (0.41 [95% CI -0.06-0.88] in random-effects versus 0.23 [95% CI 0.05–0.42] in fixed-effects).

In the realm of BI subgroup analysis, it becomes apparent that the duration of VR regimens significantly influences the outcomes of interest. Shorter VR regimens, spanning a duration of two to four weeks, as evidenced by eight independent trials (*n* = 8) [50, 52, 61, 65, 70, 71, 77, 88], yield inferior improvements in the BI, with an MD of 4.7 points. In stark contrast, the longer protocols, ranging from six to 12 weeks, as observed across five distinct trials (*n* = 5), manifest a more modest reduction in BI scores, amounting to a decrease of merely 1.3 points [39, 41, 44, 57, 80]. Studies employing VR devices as their intervention tool report the most substantial improvements, registering a remarkable increase of 7.3 points [44]. Conversely, interventions relying on robot-based technologies yield comparatively moderate advancements, resulting in an average improvement of 3.7 points, as evidenced by trials [61, 65, 70, 71, 77]. Meanwhile, programs solely grounded in game-based approaches exhibit the least pronounced improvements, with an average reduction of BI scores by 2.6 points, as supported by trials [39, 41, 50, 52, 57, 80, 88].

Subgroup analyses revealed that certain factors impacted the efficacy of VR on FMA outcomes. Shorter VR interventions of 2–4 weeks (*n* = 20 trials) resulted in lower FMA gains (MD 3.4 points) [38, 42, 43, 46, 48–52, 56–58, 60–63, 65, 69–71] compared to longer interventions of six weeks to three months (*n* = 14 trials) which improved FMA scores by 4.09 points on average [39–41, 44, 45, 47, 53–55, 59, 64, 66–68]. Subacute stroke patients within six months of onset (*n* = 18 trials) exhibited better FMA gains with VR (5.7 points) [39–41, 44, 50, 51, 56, 58–61, 64–66, 68, 70, 71] versus chronic stroke patients (*n* = 16 trials) whose improvement was 1.7 points [38, 42, 43, 45–49, 52–55, 57, 62, 63, 67, 69]. Interventions featuring advanced technologies like VR systems [38, 42, 44, 50, 51, 56–58, 62, 63] and robotic exoskeletons [40, 43, 45, 49, 54, 55, 61, 64–68, 70, 71] tend to yield better results in the experimental groups (EG) with an average improvement of approximately 4.4 and 4.7 points compare to control Group (CG) respectively, while simpler tools like commercial video games show more modest gains at around 1.7 points [39, 41, 46–48, 52, 53, 59, 60, 69]. Older participants over age 60 (*n* = 17 trials) acquired more FMA points improvement in EG compared to CG (5.1 points) [38, 40, 45, 50–52, 57–60, 62–66, 68, 71] than younger participants under age 60 (*n* = 17 trials) whose gain was 2.3 improvement points [39, 41–44, 46–49, 53–56, 61, 67, 69, 70].

#### Functional independence measure

**Fig. 9:**
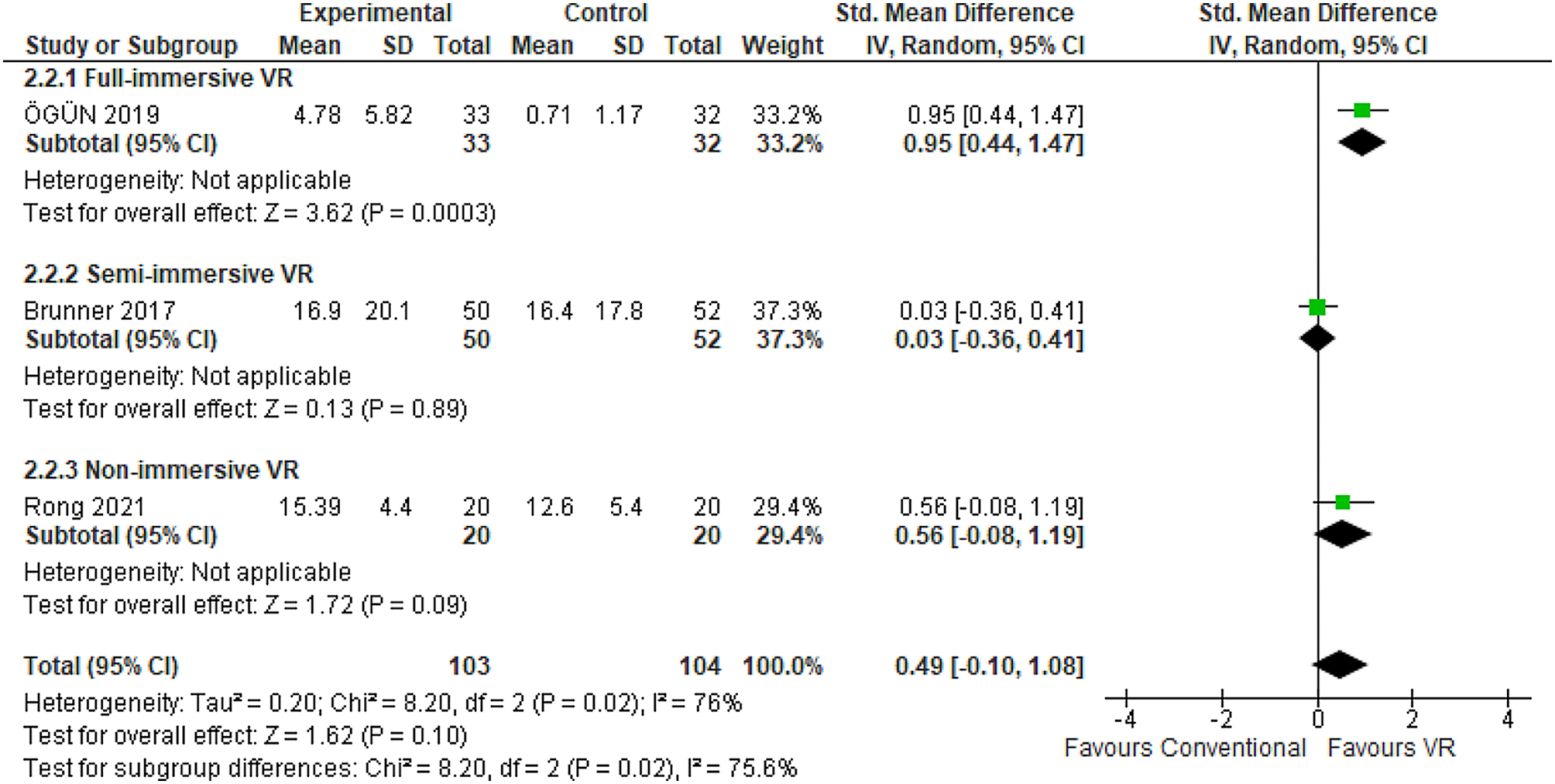
Meta-analysis forest plots: Comparing FIM Improvement in VR-based and conventional therapy

FIM enables clinicians to evaluate and stratify patients’ functional status based on their requisite degree of assistance. The FIM comprises motor and cognitive subscales. The motor subset appraises core mobility competencies including feeding, grooming, bathing, upper and lower body dressing, and toileting. The cognitive dimension evaluates communication expression, comprehension, social interaction, problem-solving, and memory. Three randomized controlled trials (*n* = 207 patients) compared VR rehabilitation against conventional occupational therapy for improving post-stroke upper extremity function using the FIM [38, 65, 73]. Considerable between-study heterogeneity was present (I^2^ = 76%). Nevertheless, this meta-analysis revealed a pooled effect size favoring VR intervention (SMD 0.49, 95% CI: -0.10-1.08) (Fig. 9). Follow-up FIM evaluations occurred two [38] to four [65, 73] weeks post-treatment. Reported mean FIM score differences varied from 0.71 [38] to 16.9 points [73] across studies. Analysis by immersion technique found fully immersive VR conferred the greatest therapeutic advantage over conventional therapy, with a mean FIM score difference of 4.07 points higher (95% CI: 3.54–4.59 points higher) [38] (Table 1). Non-immersive VR showed the next highest treatment effect versus conventional therapy, with a mean FIM increase of 2.79 points (95% CI: 2.3–3.2 points higher) [65] (Table 3). In contrast, semi-immersive VR was less effective, correlating to only a 0.5-point FIM enhancement over traditional approaches [73] (Table 2). The sensitivity analyses suggested marginal differences in FIM scores when contrasting random-effects and fixed-effect models (0.49 [95% CI -0.10-1.08] in random-effects versus 0.40 [95% CI 0.12–0.68] in fixed-effects). Analyses by intervention tools showed robotic-assisted therapies conferred higher FIM effects, with gains of 11 points [65] versus − 2.1 points for VR-based interventions [38, 73]. Mirror visual feedback coupled with robotics yielded significantly higher scores, 15.39 points, compared to standalone robotics at 4.4 points [65].

#### Postural assessment scale for stroke

PASS represents an adapted version of the FMA postural stability subscale. It comprises items of graded complexity intended to evaluate aspects of postural control including maintaining or purposefully modifying position during lying, sitting, and standing [89]. Only a single RCT (*n* = 65 patients) employing fully immersive VR utilized the PASS to assess the impact of VR versus conventional therapy [38]. This study reported mean PASS score differences ranging from 0.03 to 0.39 points and follow-up six weeks. Specifically, the VR group exhibited a mean PASS score 0.36 points higher than the conventional therapy group (95% CI 0.3 to 0.39 points higher).

### Quality of life and impact assessment

Assessment of health-related quality of life constitutes an appraisal of one’s overall well-being and satisfaction with existence. In the context of cerebrovascular accidents, it represents a salient outcome measure that can be influenced by myriad clinical and sociodemographic factors. Strokes are capable of impairing various dimensions constituting assessment of health-related quality of life, compromising functionality, and diminishing recreational and social participation for many stroke survivors after community reintegration [90, 91].

#### Stroke impact scale

**Fig. 10:**
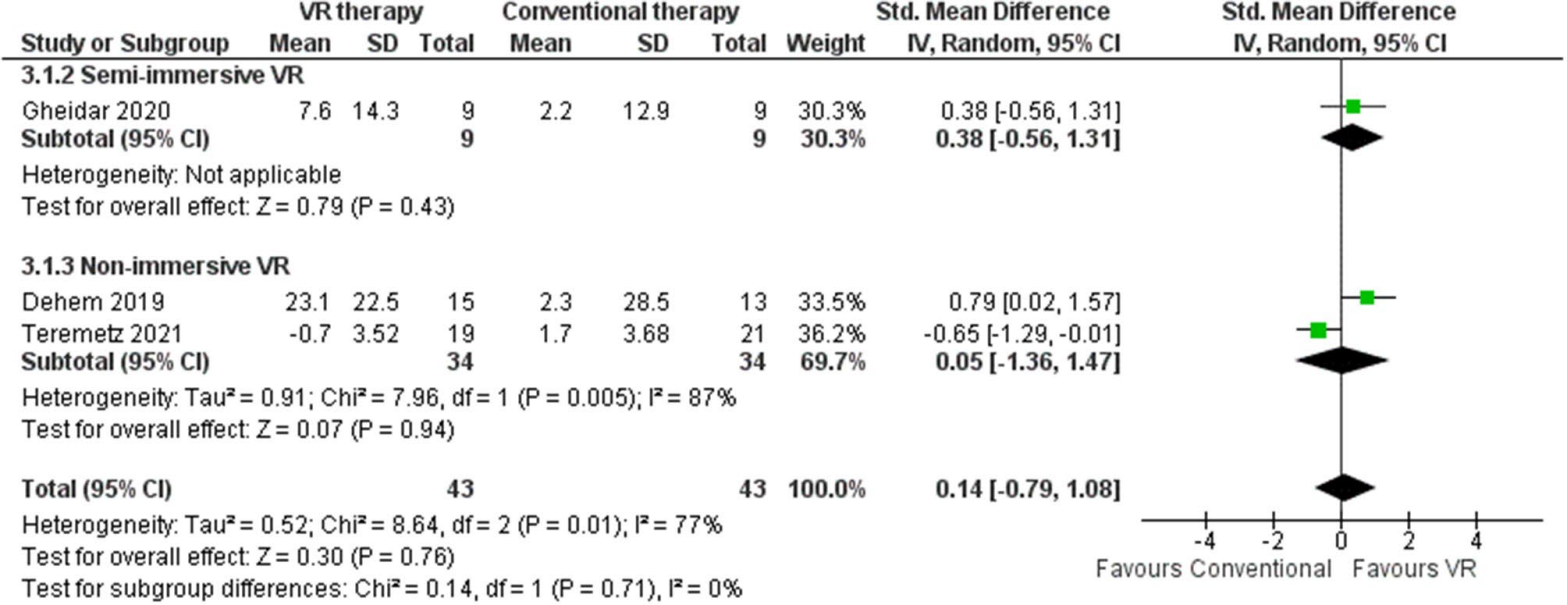
Meta-analysis forest plots: Comparing SIS improvement in VR-based and conventional therapy

SIS demonstrated utility for eliciting patient self-appraisals regarding diverse domains potentially affected by cerebrovascular accidents, including memory, cognition, manual dexterity, depressive symptoms, fatigue, and perceived severity of residual stroke-related deficits [92]. This meta-analysis identified two RCTs (*n* = 68 patients) employing the SIS to evaluate the impact of VR versus conventional interventions, with 77% heterogeneity and follow-up ranging from four to 24 weeks [40, 69]. Both studies utilized non-immersive VR approaches. The pooled effect size slightly favored VR but was modest in magnitude (SMD 0.14, 95% CI: -0.79-1.08). The maximum SIS score difference was 23.1 points [40] and the minimum was − 0.7 points [69] (Fig. 10). Non-immersive VR elicited a mean SIS score increase of 9.2 points higher than conventional therapy exhibiting a two-point mean difference (Table 3). The sensitivity analyses indicated variations in SIS scores when comparing random-effects and fixed-effect models (0.14 [95% CI -0.79, 1.08] in random-effects versus 0.03 [95% CI -0.41, 0.46] in fixed-effects). Comparisons by intervention tool showed using the game-based VR system conferred lower effects of -5.5 points than CG [42] compared to robotic devices at 0.6 points [40]. Younger age cohorts (< 60 years) with low disease onset (four weeks) achieved lower points [42, 69] than older patients who had longer disease onset (six months) [40].

### Muscle spasticity assessment

Involuntary muscular contractions during movement, termed spasticity, frequently occur in the elbow, wrist, and ankle following cerebrovascular incidents resulting in impaired neuromuscular control. When a muscle’s range of motion becomes restricted in its capacity for complete extension or flexion, the associated tendons and peri-muscular tissues can develop heightened tone as well, compounding challenges of muscle lengthening through stretching maneuvers [93]. Therefore, determining the severity of musculotendinous hypertonia in post-stroke patients is indispensable for clinical appraisal and rehabilitative planning. Several assessment tools and ordinal rating scales are utilized to quantify spasticity, including the modified Ashworth scale [94].

#### Modified ashworth scale

**Fig. 11:**
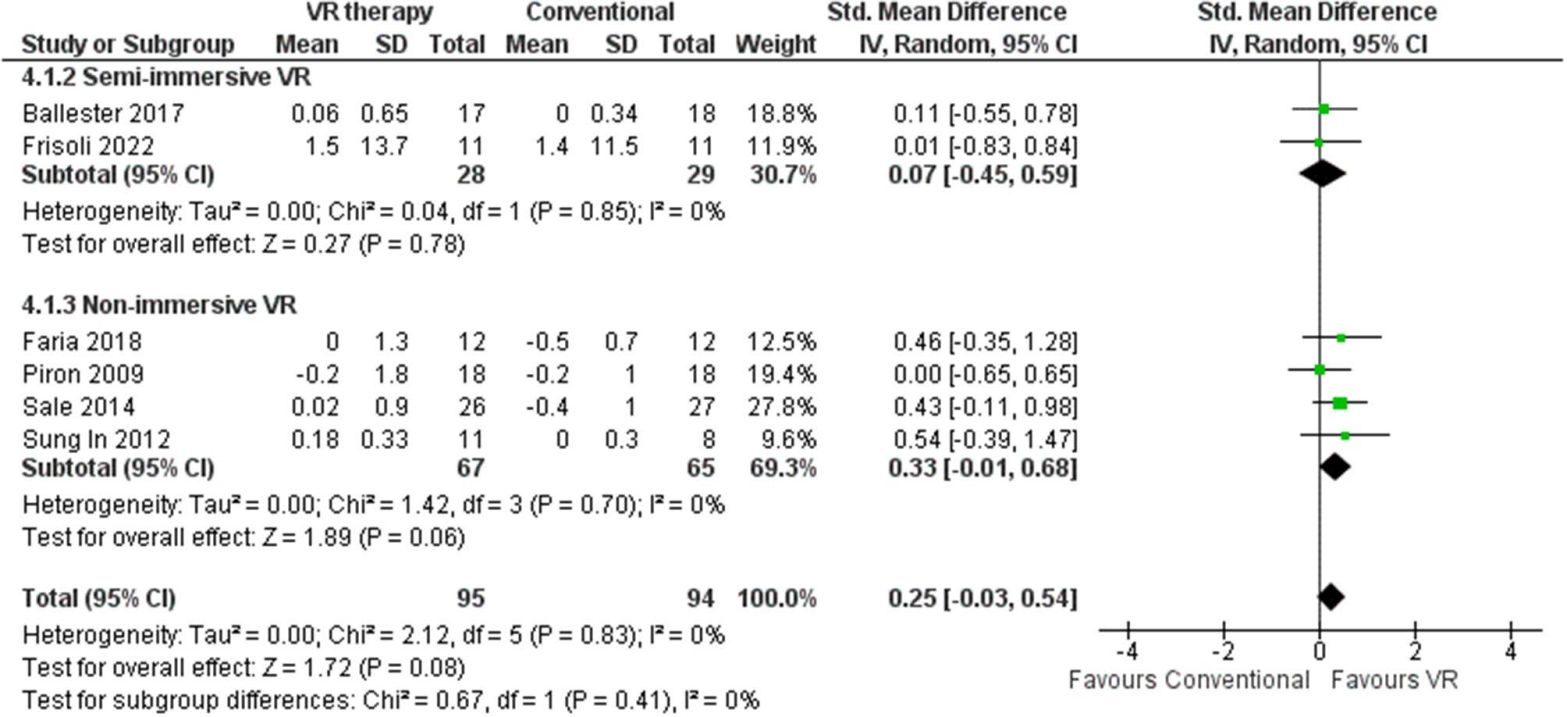
Meta-analysis forest plots: Comparing MAS improvement in VR-based and conventional therapy

MAS provides an objective quantification of musculotendinous resistance by measuring the angular joint position at which clinical examiners first detect impedance to passive mobilization caused by an involuntary “catch” of hypertonic musculature [95]. Specifically, this assessment tool evaluates the degree of flexion or extension range of motion where the practitioner encounters heightened resistance from spastic muscles during gentle manipulation of the affected limb. The meta-analysis of six RCTs comprising 189 participants revealed no statistical heterogeneity across studies (I^2^ = 0%), indicating consistent intervention effects [45, 57, 58, 63, 66, 80]. VR conferred a small benefit over traditional rehabilitation with a pooled effect size of 0.25, (95% CI: -0.03-0.54) and did not definitively exclude the possibility of no effect (Fig. 11). Follow-up occurred four [58] to 12 weeks [80] post-stroke. Comparison of mean MAS score differences showed a range from − 0.5 [57] to 1.5 points [45] favoring VR over conventional care. Non-immersive VR elicited greater gains than conventional therapy (MD 0.27 points) [57, 58, 63, 66] (Table 3) compared to semi-immersive modalities (MD 0.08 points) [45, 80] (Table 2). The sensitivity analyses disclosed no variations in MAS scores when comparing random-effect and fixed-effect models.

Notably, no studies utilizing fully immersive VR incorporated the MAS, precluding conclusions about this technique’s relative efficacy. Analyses comparing intervention tools found that studies using gaming rehabilitation devices conferred significantly greater benefits, lowering average MAS scores by 0.28 points in EG compared to CG [57, 80] relative to non-game methods such as robotic [45, 66] and VR interventions [58, 63] which reduced scores by 0.26 and 0.09 points, respectively. Adverse effects reported were uncommon and mild, such as rare transient fatigue. Patients who had lower stroke onset had higher improvement in mean MAS in EG at 0.42 point [66] compared to cases that had greater than one month of disease onset [45, 57, 58, 63, 80].

### Functional use and dexterity

Functional limb use refers to the ability of an affected extremity to execute meaningful activities of daily living. This encompasses one’s capacity to independently dress, self-groom, and perform household chores. Manual dexterity denotes the fine motor skills required to carry out delicate movements such as buttoning clothes or writing with a pen [96].

#### Motor activity log – assessment of use

**Fig. 12:**
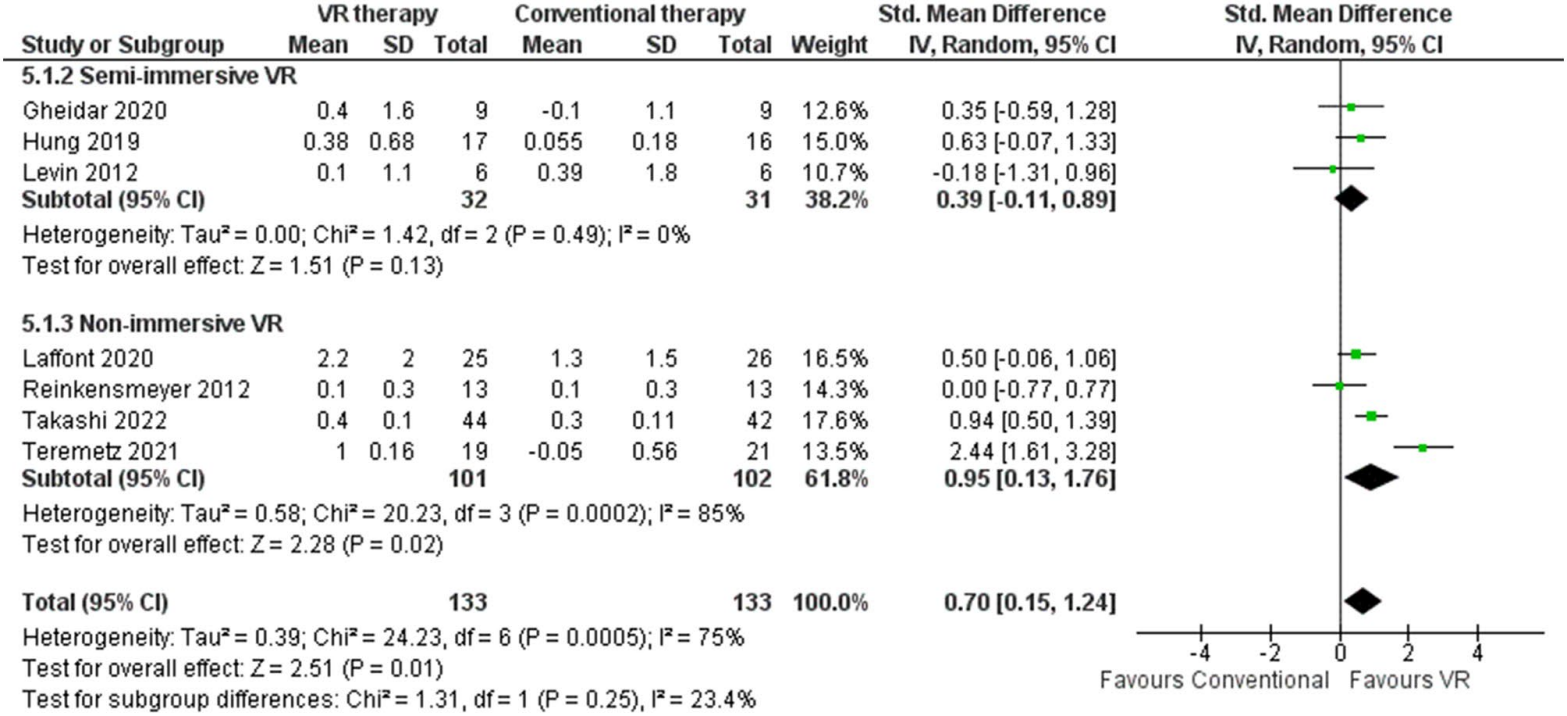
Meta-analysis forest plots: Comparing MAL-AOU improvement in VR-based and conventional therapy

MAL-AOU subscale quantifies patients’ self-reported utilization of the paretic upper limb. It is employed to evaluate the perceived engagement and capacity of the impaired arm during activities of daily living. Specifically, the MAL-AOU measures subjective arm usage and quality of movement while executing common daily tasks [56]. Seven RCTs (*n* = 266 patients) were identified that compared VR-based and conventional rehabilitation for improving post-stroke paretic arm function using the MAL-AOU [41, 42, 47, 48, 64, 67, 69]. Considerable between-study heterogeneity was present (I^2^ = 75%), with follow-up ranging from three weeks [48] to six months [41]. VR demonstrated superior outcomes to conventional therapy per a large pooled effect size (0.70, 95% CI: 0.15–1.24) (Fig. 12). The maximum and minimum MAL-AOU score differences were 2.2 [41] and − 0.1 [42] points, respectively. Analysis by immersion technique showed non-immersive VR elicited a 0.51 point (95% CI: 0.48–0.54) greater improvement than conventional care [41, 67, 69, 82] (Table 3), outperforming semi-immersive VR which produced a 0.18 point (95% CI: 0.04–0.31) larger effect [42, 47, 48] (Table 2). No studies were identified employing fully immersive VR and the MAL-AOU to evaluate post-stroke arm function relative to conventional therapy. The sensitivity analyses indicated disparities in the MAL-AOU scores when comparing random-effects and fixed-effect models (0.70 [95% CI 0.15–1.24] in random-effects versus 0.74 [95% CI 0.49-1.00] in fixed-effects). Intervention tools using Wii Sports games of tennis, golf, and boxing yielded higher effects, elevating MAL-AS by an average higher improvement of 1.05 points in EG [69] relative to other interventions [41, 42, 47, 48, 64, 67]. Younger patients (< 50 years) achieved on average higher improvement of 0.15 more points [42] than older cohorts [41, 47, 48, 64, 67, 69].

#### Box and block test

**Fig. 13:**
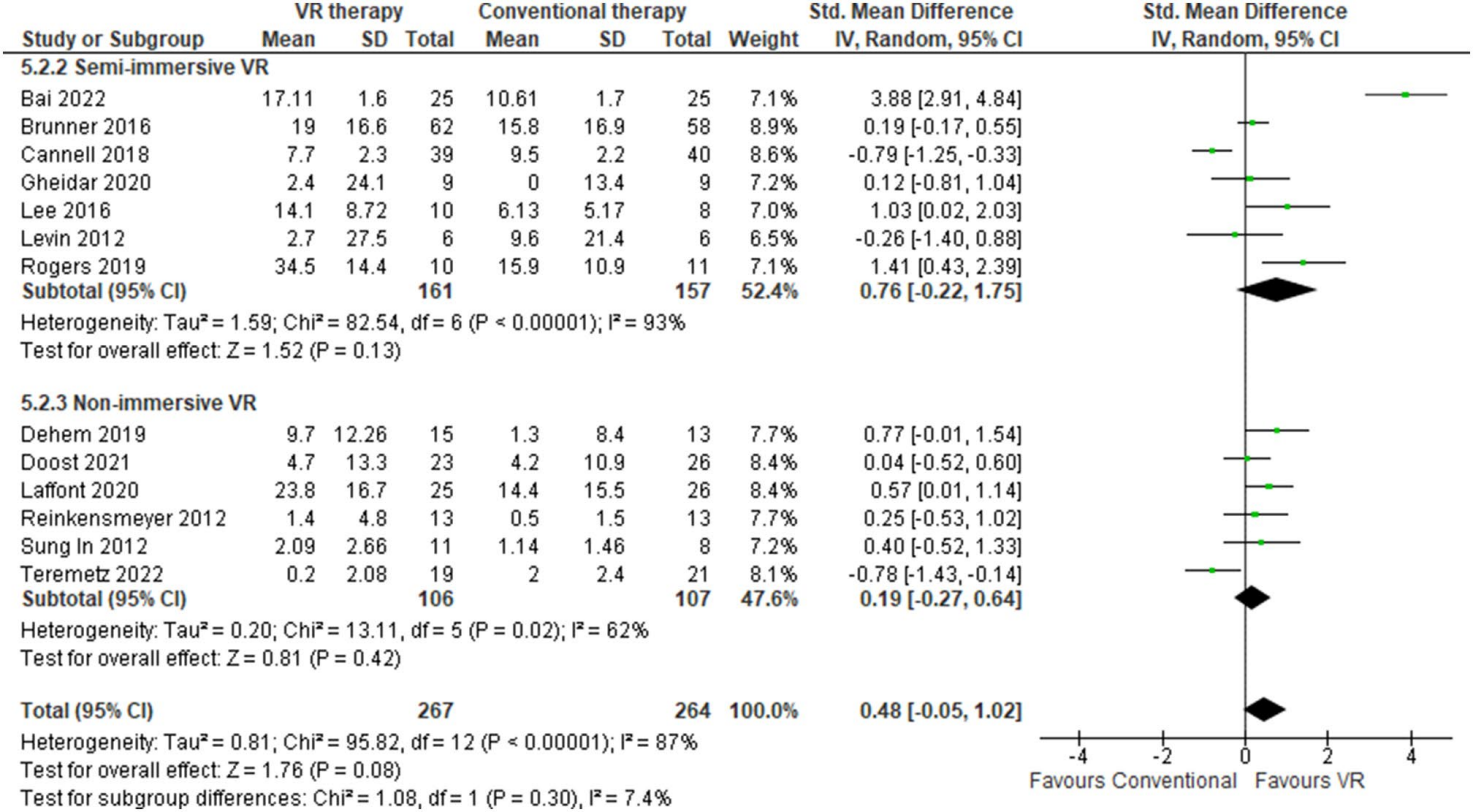
Meta-analysis forest plots: Comparing BBT improvement in VR-based and conventional therapy

BBT assesses gross manual dexterity by having subjects transfer blocks between compartments within a designated timeframe. Specifically, the BBT quantifies unilateral fine motor skills by recording the number of one-inch cubes a patient can grasp individually and transport from one section of a box to another across a divider using only their hand of interest for 60 s [97]. This meta-analysis incorporated 13 studies with a total of 531 patients [7, 40–42, 44, 48, 58, 64, 69, 73, 78, 81, 98]. Considerable heterogeneity (I^2^ = 87%) was observed in follow-up duration, ranging from 2 days to 24 weeks. VR-based approaches demonstrated moderately superior effects (pooled SMD 0.48, 95% CI: -0.05-1.2) compared to traditional interventions for upper limb motor recovery after stroke according to BBT assessments (Fig. 13). The maximum MD in BBT performance was 34.5 blocks [98], while the minimum MD was zero blocks favoring VR [42]. Furthermore, a comparison of VR systems with varying levels of immersion revealed semi-immersive VR modalities resulted in an MD of 4.3 blocks (95% CI: 3.98–4.57) greater than conventional therapy [42, 44, 48, 73, 78, 81, 98] (Table 2), outperforming non-immersive VR approaches which had an MD of 3.05 blocks (95% CI: 2.74–3.38) over controls [7, 40, 41, 58, 69, 82] (Table 3). The sensitivity analyses revealed differences in the BBT scores when comparing random-effects and fixed-effect models (0.48 [95% CI -0.05-1.02] in random-effects versus 0.21 [95% CI 0.03–0.39] in fixed-effects).

Subgroup analyses showed a significant differential treatment effect was elucidated between older (≥ 60 years) and younger (< 60 years) subgroups which were larger in older patients (MD 5.8 blocks) [7, 40, 58, 78, 81, 98] compared to their younger counterparts (MD 1.9 blocks) [41, 42, 44, 48, 64, 69, 73]. Although the differential treatment effect tended to be greater in studies with more chronic stroke survivors (MD 5.8 blocks in < six months) [40, 41, 44, 58, 64, 73, 78, 98] compared to acute patients (MD 0.4 blocks in ≥ six months) [7, 42, 48, 57, 69, 81], this discrepancy failed to attain statistical significance, potentially owing to insufficient power from the modest sample size. The experimental-control group difference in BBT change was meaningfully larger in trials employing interventions longer than six weeks (MD 4.7 blocks) [40, 41, 44, 64, 78] compared to briefer protocols (MD 3.1 blocks) [7, 42, 48, 58, 69, 73, 81, 98]. The discrepancy in BBT change scores between experimental and comparator arms was greatest amongst trials utilizing more immersive VR systems (MD 7.4 blocks) [44, 58, 73, 81, 98], followed by video gaming (MD 3.2 blocks) [41, 42, 48, 56, 69, 78], and lastly robot-assisted methods (MD 0.3 blocks) [7, 40, 64].

#### Upper extremity functional index (UEFI)

UEFI is a patient-reported outcome measure that evaluates an individual’s capacity to perform various upper limb activities through a self-administered questionnaire. Specifically, the UEFI assesses tasks requiring the use of the upper extremities such as lifting, reaching, carrying, and manipulating objects. Respondents indicate their perceived difficulty in performing each of the 20 movement-based activities enumerated in the UEFI on a Likert scale [99]. These meta-analytical findings demonstrated that among the included RCTs, only one study utilizing a sample of 20 participants deployed a fully immersive VR system to assess efficacy compared to traditional techniques as measured by the UEFI with follow-up durations ranging from one to six weeks [79]. This solitary RCT documented MD ranging from 9.3 to 12.6 points on the UEFI. Specifically, the MD in UEFI score for the VR group was 3.3 points higher (95% CI: 2 lower to 8.7 higher) than the MD of 9.3 points for the conventional therapy group (Table 1).

### Breathing improvement

One RCT study examined the effects of a five-week game-based breathing exercise (GBE) program on pulmonary function in 38 stroke patients [100]. Participants were randomized to either the GBE group (*n* = 19) or a control group (*n* = 19). Both groups received conventional stroke rehabilitation, but only the GBE group did 25 min of game-based breathing exercises three days per week. Pulmonary function tests of forced vital capacity (FVC), forced expiratory volume in one second (FEV1), FEV1/FVC ratio, and maximum voluntary ventilation (MVV) were measured before and after the five-week intervention. Compared to controls, the GBE group showed significantly greater improvements in FVC, FEV1, and MVV after training (*p* < 0.05). No between-group differences were found for FEV1/FVC. These results provide preliminary evidence that incorporating game-based breathing exercises into rehabilitation may benefit pulmonary function in stroke patients. The interactive gaming format could enhance motivation and respiratory muscle training versus conventional breathing exercises.

## Discussion

The current systematic review and meta-analysis comprehensively examined data derived from 55 RCTs, encompassing a cohort of 2,142 stroke patients. The primary objective was to assess the effectiveness of VR interventions tailored for entire upper limb rehabilitation compared to conventional therapeutic modalities. These interventions comprised a range of VR modalities, spanning non-immersive, semi-immersive, and fully immersive configurations aimed at creating immersive rehabilitation environments. Our findings underscore the adjunctive role of VR-based rehabilitation in enhancing upper limb motor recovery across diverse functional domains, including motor function, functional independence, quality of life, spasticity, and dexterity, relative to conventional occupational therapy post-stroke. This analysis advocates for integrating VR as a supplementary component rather than a complete substitute for conventional therapeutic approaches.

In terms of motor function, our meta-analysis revealed a significant advantage of VR interventions over conventional therapy, as indicated by a SMD of 0.63, as assessed by FMA. This finding aligns with previous systematic reviews and meta-analyses, which also highlighted improvements in upper limb motor function through VR-based rehabilitation. Notably, studies by Domınguez-Tellez et al. [101], Okamura et al. [102], Subramanian et al. [103], and Hao et al. [104] reported higher SMD values of 1.53, 0.81, 0.75, and 0.68, respectively, in favor of VR interventions for upper limb function. Conversely, Al-Whaibi et al. [105], Gao et al. [106], Maier et al. [27], Jin et al. [107], Wang et al. [108], Aminov et al. [109], Peng et al. [110], Chen et al. [21], and Meng et al. [111] reported slightly lower SMD values ranging from 0.15 to 0.5 in favor of VR interventions. Additionally, Leong et al. [112] demonstrated a MD of 3.91, and Kiper et al. [113] reported a MD of 6.33, both indicating benefits in favor of VR-based rehabilitation.

Subtotal analysis unveiled nuanced outcomes based on the immersive nature of VR systems for motor function. Fully immersive setups were associated with significant gains assessed by FMA, boasting a SMD of 1.76, whereas semi-immersive modalities exhibited greater enhancements in fine dexterity assessed by ARAT, yielding a SMD of 3.53. This observation resonates with the conclusions drawn from the systematic review by Hao et al. [104], which suggested that immersive VR environments might be particularly advantageous for improving gross motor function (SMD = 1.44), while non-immersive setups, including non-immersive VR (SMD = 0.86), gaming consoles using Microsoft Kinect (SMD = 0.50), and Nintendo Wii (SMD = 0.01), could be more conducive to refining fine motor skills.

These findings suggest a consistent and reproducible impact of VR interventions on upper limb motor function, underscoring the reliability and applicability of the observed benefits in stroke patients. Plausible rationales for this consistency stem from the immersive and captivating nature of VR environments, fostering heightened patient engagement and motivation during rehabilitation sessions. Additionally, the adaptable nature of VR interventions allows for customized rehabilitation protocols, addressing specific motor deficits and adapting to the unique needs of individual patients. The real-time feedback provided by VR systems empowers patients to monitor their progress and refine their movements accordingly, augmenting motor learning and retention. The potential superiority of immersive VR in enhancing gross motor function may be attributed to its ability to provide a highly immersive and realistic simulated environment, promoting enhanced motor learning mechanisms and facilitating neuroplasticity. Tailoring VR interventions to the distinct rehabilitation objectives and requirements of stroke patients is crucial, with fully immersive setups potentially eliciting greater patient motivation and engagement, while non-immersive approaches might provide more targeted and precise training opportunities, particularly beneficial for honing fine motor skills and coordination. Hence, clinicians and therapists should meticulously consider the individual characteristics and needs of each patient when selecting the most appropriate VR rehabilitation modality.

Moreover, our meta-analysis demonstrated significant improvements in functional independence, as measured by BI, with the SMD of 0.41. This finding resonates with previous systematic reviews, which also observed enhanced ADL performance following VR-based rehabilitation post-stroke, with SMD values ranging from 0.24 to 2.37 [17, 27, 101–103, 106, 108, 111, 113]. Our results suggest that VR interventions can result in tangible enhancements in real-world functional abilities, potentially by optimizing motor learning and facilitating skill transfer through immersive, task-specific training paradigms.

The present meta-analysis disclosed noteworthy improvements in quality of life, as assessed by SIS (SMD = 0.14), and a reduction in spasticity, measured by MAS (SMD = 0.25), following VR interventions. The observed enhancements in quality of life may stem from the multifaceted benefits of VR interventions, including improvements in motor function, increased independence in ADL, and mitigation of spasticity. The observed slight increase in spasticity, suggesting that VR-based interventions may facilitate sensory input, motor learning, and cortical reorganization.

Furthermore, our meta-analysis demonstrated significant enhancements in dexterity (SMD = 0.70) following VR interventions, consistent with findings reported by Aminov et al. (SMD = 0.38) [109] and Jin et al. (SMD = 0.09) [107], indicating improved fine motor skills and dexterity post-stroke. Notably, higher levels of immersion were associated with greater functional improvements, as reported by Gao et al. [106] (SMD = 0.62) and Kiper et al. (SMD = 0.58) [113], suggesting that the enriched sensory feedback provided by immersive VR environments may facilitate the execution of complex daily tasks.

Subgroup analyses revealed that interventions exceeding six weeks in duration yielded superior results across various outcome measures, suggesting the potential necessity for longer treatment durations to maximize the benefits of VR rehabilitation. For instance, interventions longer than six weeks demonstrated greater improvements in motor function compared to shorter interventions (MD = 0.7). This finding aligns with previous studies indicating that VR interventions lasting longer than four weeks or conducted more frequently may yield more significant benefits [103, 106], although some studies did not report significant differences between trial length and FMA improvement [105, 107, 110]. Additionally, initiating VR interventions within six months post-stroke appeared to optimize outcomes, with greater motor function improvements observed when VR was initiated within this timeframe (six-point improvement in MD) compared to later time points. This underscores the importance of early rehabilitation for leveraging neuroplasticity and facilitating recovery. Capitalizing on this critical window for heightened neural reorganization and recovery is advised. Interestingly, younger cohorts exhibited lower gains on motor function tests compared to older adults, contrasting assumptions that age impairs rehabilitation potential. This highlights the need for further investigation into the influence of age and other patient characteristics on VR intervention outcomes.

While our findings demonstrate the overall efficacy of VR interventions for upper limb rehabilitation after stroke, substantial heterogeneity across studies was observed, particularly concerning VR systems, intervention protocols, and outcome measures. This variability likely contributed to the differing effect sizes observed across different outcome domains and subgroups. For instance, SMDs for motor function improvements ranged from 0.31 for fully immersive VR systems to 0.58 for non-immersive VR approaches and exhibited variations in previous studies, ranging from 0.15 [105] to 1.53 points [101]. Similar discrepancies were reported in previous meta-analyses, underscoring the need for standardization in VR intervention protocols and outcome assessments to enable direct comparisons and identify optimal approaches. Further investigation into factors contributing to heterogeneity, such as specific VR system characteristics and task complexity, would also be valuable in optimizing VR interventions for stroke rehabilitation.

Furthermore, it is essential to address the potential limitations and challenges inherent in VR interventions. While our review focused on efficacy, feasibility, and safety aspects merit consideration. Previous studies have raised concerns about cybersickness, balance issues, and the need for specialized training and supervision. Additionally, the cost and accessibility of VR systems, especially fully immersive setups, may hinder widespread implementation in clinical settings [114, 115]. These variations in VR system complexity, cost, and accessibility requirements could contribute to the observed heterogeneity across studies. Moving forward, research efforts should continue to address these practical considerations to facilitate the effective and safe integration of VR technologies into rehabilitation practices. This entails developing more affordable and user-friendly VR solutions, optimizing training protocols for safe and efficient use, and establishing guidelines for managing potential adverse effects like cybersickness.

### Strengths and limitations

A key strength of this systematic review and meta-analysis is its comprehensive search strategy and adherence to PRISMA guidelines, ensuring a rigorous methodology. The inclusion of a large number of randomized controlled trials and participants across various outcome domains enhances the robustness and generalizability of the findings. Additionally, the stratification of VR interventions based on immersion levels and subgroup analyses provides valuable insights into the potential differential effects of different VR modalities. However, this review is not without limitations. Despite efforts to identify all relevant studies, the possibility of publication bias cannot be entirely excluded. Furthermore, substantial heterogeneity among the included studies in terms of VR systems, intervention protocols, and outcome measures may have influenced the effect size estimates. While subgroup analyses were conducted based on available data, inconsistent reporting of certain variables, such as stroke severity and upper limb impairment levels, limited the ability to perform more detailed subgroup comparisons.

One prominent limitation across the synthesized studies pertains to small sample sizes, with many trials incorporating fewer than 30 participants per study arm. Such modest sample sizes restrict the generalizability of results and introduce imprecision in the effect size estimates. Additionally, the predominance of short intervention durations under six weeks across studies raises uncertainty about the long-term sustainability of gains from VR interventions. Considerable heterogeneity in intervention protocols, including the VR delivery platforms utilized, introduces variability in the findings. Moreover, few trials employed comprehensive multidimensional assessments, limiting the ability to elucidate the broad impacts of VR across various domains of functioning. High dropout rates in some investigations raise concerns about the feasibility and adherence to VR interventions in clinical practice. The lack of participant blinding was ubiquitous across studies, potentially incurring performance bias in the outcomes. Collectively, these limitations underscore the preliminary nature of the current evidence base and emphasize the need for more rigorous methodology and standardized protocols in subsequent research elucidating the utility of VR in post-stroke rehabilitation. Addressing these shortcomings through larger, well-designed trials with standardized protocols, comprehensive outcome measures, and robust feasibility evaluations represents imperative next steps for advancing the field and establishing the clinical potential of VR for stroke rehabilitation.

## Conclusions

This systematic appraisal delineates salient best practice considerations for thoughtfully incorporating VR technologies into post-stroke upper extremity rehabilitation regimens. The meta-analytic findings indicate employing fully immersive VR modalities serves to optimize recovery of gross motor skills of the paretic limb, while less immersive platforms may confer greater benefits for remediating fine motor dexterity deficits. Additionally, initiating VR-based interventions during the critical period of heightened neuroplasticity within the first six months following a cerebrovascular accident and continuing therapy for an adequate duration exceeding six weeks appears vital for eliciting maximal therapeutic gains. Moreover, personalized customization of VR activities tailored to each patient’s discrete motor and functional capabilities is advisable. Further research into VR’s impacts on the multidimensional sequelae of stroke is warranted. However, the current systematic review provides substantive evidence that supplemental integration of VR-based techniques, when thoughtfully implemented, confers additional benefits for improving upper extremity motor function and performance of activities of daily living compared to conventional occupational therapy alone. This supports VR as a valuable rehabilitation modality for enhancing outcomes in stroke survivors. Ongoing optimization of VR protocols may further potentiate its utility.

### Electronic supplementary material

Below is the link to the electronic supplementary material.

Supplementary Material 1

Supplementary Material 2

Supplementary Material 3

Supplementary Material 4

## Data Availability

The datasets supporting the conclusions of this article are included within the article and its additional files.

## Abbreviations

FMA: Fugl-Meyer Assessment
ARAT: Action Research Arm Test
WMFT: Wolf Motor Function Test
BI: Barthel Index
BBT: Box and Block Test
MAS: Modified Ashworth Scale
SIS: Stroke Impact Scale
VR: Virtual Reality
PICO: Population, Intervention, Comparison, and Outcome
RCT: Randomized Controlled Trials
MMT: Manual Muscle Testing
ROM: Range of Motion
FIM: Functional Independence Measure
MAL-AOU: Motor Activity Log–Amount of Use
MD: Mean Differences
SMD: Standardized Mean Differences
JTHFT: Jebsen-Taylor Hand Function Test
EG: Experimental Groups
CG: Control Group
FIAS: Functional Independence Assessments
IADL: Activities of Instrumental Daily Living
PASS-BADL: Postural Assessment Scale for Stroke Evaluating Basic ADLS
PASS-IADL: Postural Assessment Scale for Stroke Assessing IADLS
UEFI: Upper Extremity Functional Index
GBE: Game-based Breathing Exercise
FVC: Forced Vital Capacity
FEV1: Forced Expiratory Volume in One Second
