# Supplementary material for: The efficacy of virtual reality for upper limb rehabilitation in stroke patients: a systematic review and meta-analysis
Source: BMC Med Inform Decis Mak. 2024 May 24;24:135. doi: 10.1186/s12911-024-02534-y (PMC11127427; doi:10.1186/s12911-024-02534-y)
Supplement: Supplementary file 1 — Supplementary Material 1 [file 12911_2024_2534_MOESM1_ESM.docx]

**Additional File 1**

**1.1. Pubmed search strategy:**

(((Stroke*[tiab] OR cva[tiab] OR Cerebrovasc*[tiab] OR Poststroke*[tiab] OR post-stroke*[tiab] OR Hemipleg*[tiab] OR paretic[tiab] OR apoplexy[tiab] OR paresis[tiab] OR Hemiparesis[tiab] OR Neuroplasticity[tiab]) OR ((cerebral*[tiab] OR Brain*[tiab] OR cerebellar*[tiab]) AND (infarct*[tiab] OR isch!emi[tiab] OR thrombo*[tiab] OR emboli*[tiab] OR apoplex*[tiab] OR attack*[tiab] OR vascular*[tiab] OR injur*[tiab])) OR ((cerebral*[tiab] OR Brain*[tiab] OR cerebella*[tiab]) AND (haemorrhage*[tiab] OR hemorrhage*[tiab] OR haematoma*[tiab] OR hematoma*[tiab] OR bleed*[tiab])) OR "Transient ischemic attack"[tiab] OR TIA[tiab] OR "Subarachnoid hemorrhage"[tiab] OR "Gait Disord*"[tiab]) AND ((Comput*[tiab] OR assist*[tiab] OR simulat*[tiab] OR genera*[tiab] OR cyber*[tiab] OR virtual*[tiab] OR visual*[tiab] OR immers*[tiab] OR "semi*immers*"[tiab] OR metaverse[tiab] OR 3D[tiab] OR augment*[tiab] OR Digit*[tiab] OR "virtual* real*"[tiab] OR "virtual-real*"[tiab] OR VR[tiab] OR "augment* real*" [tiab] OR "augment*-real*"[tiab] OR AR[tiab] OR "Mix* real*" [tiab] OR "Mix*-real*" [tiab] OR "video* game*" [tiab] OR "interact* game*" [tiab] OR "Serious game*" [tiab] OR Gamificat*[tiab])) AND (Rehab*[tiab] OR recovery[tiab] OR restore*[tiab] OR Habilitat*[tiab] OR therapy[tiab] OR treat*[tiab] OR care[tiab] OR plan*[tiab] OR intervent*[tiab] OR approach*[tiab] OR interact*[tiab] OR Counsel*[tiab] or Heal*[tiab] OR aid*[tiab] OR help*[tiab] OR relief*[tiab] OR well*[tiab] OR support[tiab] OR program[tiab] OR software[tiab] OR system[tiab] OR device[tiab] OR technique*[tiab]) AND ("free full text" [sb] AND humans[mh] AND Eng [la] AND ("Clinical Trial" [pt] OR "Randomized Controlled Trial" [pt] OR RCT[pt])))

**1.2. Scopus search strategy:**

(((TITLE-ABS-KEY(Stroke*) OR TITLE-ABS-KEY(CVA) OR TITLE-ABS-KEY(Cerebrovas*) OR TITLE-ABS-KEY(Poststroke*) OR TITLE-ABS-KEY(post-stroke*) OR TITLE-ABS-KEY(Hemipleg*) OR TITLE-ABS-KEY(paretic) OR TITLE-ABS-KEY(apoplexy) OR TITLE-ABS-KEY(paresis) OR TITLE-ABS-KEY(Hemipare*) OR TITLE-ABS-KEY(Neuroplast*)) OR ((TITLE-ABS-KEY(cerebral*) OR TITLE-ABS-KEY(Brain*) OR TITLE-ABS-KEY(cerebellar)) AND (TITLE-ABS-KEY(infarct*) OR TITLE-ABS-KEY(ischemi*) OR TITLE-ABS-KEY(thrombo*) OR TITLE-ABS-KEY(emboli*) OR TITLE-ABS-KEY(apoplex*) OR TITLE-ABS-KEY(attack*) OR TITLE-ABS-KEY(vascular*) OR TITLE-ABS-KEY(injur*))) OR ((TITLE-ABS-KEY(cerebral) OR TITLE-ABS-KEY(Brain) OR TITLE-ABS-KEY(cerebella)) AND (TITLE-ABS-KEY(haemorrhage) OR TITLE-ABS-KEY(hemorrhage) OR TITLE-ABS-KEY(haematoma) OR TITLE-ABS-KEY(hematoma) OR TITLE-ABS-KEY(bleed*))) OR TITLE-ABS-KEY(“Transient ischemic attack”) OR TITLE-ABS-KEY(TIA) OR TITLE-ABS-KEY(“Subarachnoid hemorrhage”) OR TITLE-ABS-KEY(“Gait Disorders”)) AND (TITLE-ABS-KEY(Comput*) OR TITLE-ABS-KEY(asisst*) OR TITLE-ABS-KEY(Comput* W/2 assist*) OR TITLE-ABS-KEY(simulat*) OR TITLE-ABS-KEY(comput* W/2 base*) OR TITLE-ABS-KEY(genera*) OR TITLE-ABS-KEY(cyber*) OR TITLE-ABS-KEY(virtual*) OR TITLE-ABS-KEY(immers*) OR TITLE-ABS-KEY("semi* immers*") OR TITLE-ABS-KEY(metaverse*) OR TITLE-ABS-KEY(3D*) OR TITLE-ABS-KEY(augment*) OR TITLE-ABS-KEY(digit*) OR TITLE-ABS-KEY(visual*) OR TITLE-ABS-KEY(“virtual reality”) OR TITLE-ABS-KEY(virtual W/2 reality) OR TITLE-ABS-KEY(VR) OR TITLE-ABS-KEY(“augmented reality”) OR TITLE-ABS-KEY(augmented W/2 reality) OR TITLE-ABS-KEY(AR) OR TITLE-ABS-KEY(“Mixed reality”) OR TITLE-ABS-KEY(Mixed W/2 reality) OR TITLE-ABS-KEY(game*) OR TITLE-ABS-KEY(“Video* game*”) OR TITLE-ABS-KEY(Video W/2 game) OR TITLE-ABS-KEY(interact* W/2 game*) OR TITLE-ABS-KEY(Serious* W/2 game*) OR TITLE-ABS-KEY(Gamificat*)) AND (TITLE-ABS-KEY(Rehab*) OR TITLE-ABS-KEY(recovery) OR TITLE-ABS-KEY(restore*) OR TITLE-ABS-KEY(Habilitat*) OR TITLE-ABS-KEY(therapy) OR TITLE-ABS-KEY(treat*) OR TITLE-ABS-KEY(care) OR TITLE-ABS-KEY(plan*) OR TITLE-ABS-KEY(intervent*) OR TITLE-ABS-KEY(approach*) OR TITLE-ABS-KEY(interact*) OR TITLE-ABS-KEY(Counsel*) or TITLE-ABS-KEY(Heal*) OR TITLE-ABS-KEY(aid*) OR TITLE-ABS-KEY(help*) OR TITLE-ABS-KEY(relief*) OR TITLE-ABS-KEY(well*) OR TITLE-ABS-KEY(support) OR TITLE-ABS-KEY(program) OR TITLE-ABS-KEY(software) OR TITLE-ABS-KEY(system) OR TITLE-ABS-KEY(device) OR TITLE-ABS-KEY(technique*)) AND (TITLE-ABS(“Clinical Trial*”) OR TITLE-ABS(“Randomized Control* Trial*”) OR TITLE-ABS(“RCT”)) AND (OA(ALL) AND LANGUAGE(English) AND SRCTYPE(j) AND DOCTYPE(ar)))

**1.3. IEEE search strategy:**

((("Document Title":Stroke OR CVA OR Cerebrovascular OR Poststroke OR "post stroke" OR Hemiplegia OR paretic OR apoplexy OR paresis OR hemiparetic OR hemiparesis OR Neuroplasticity) OR (("Document Title":cerebral OR Brain OR cerebellar) AND ("Document Title":infarction OR ischemic OR thrombo* OR embolic OR attack OR vascular OR injury or injuries)) OR (("Document Title":cerebral OR Brain OR cerebella) AND ("Document Title":haemorrhage OR hemorrhage OR haematoma OR hematoma OR bleeding)) OR ("Document Title":"Transient ischemic attack" OR TIA OR "Subarachnoid hemorrhage" OR "Gait disorder")) AND (("Document Title":Computer OR assistant OR “Computer assisted” OR simulator OR simulation OR simulate OR “computer based” OR generate OR generation OR cyber OR virtual OR immersive OR immersion OR "semi immersive" OR metaverse OR 3D OR augmented OR digital OR visual OR ”virtual reality” OR VR OR ”augmented reality” OR AR OR ”Mixed reality” OR game OR games OR ”Video game” OR “interactive game" OR “interactive games" OR “Serious game" OR “Serious games" OR Gamification)) AND (("Document Title":Rehabilitation OR Rehab OR recovery OR restore OR restoration OR Habilitate OR Habilitation OR therapy OR treat OR treatment OR care OR plan OR intervention OR approach OR interaction OR Counseling or Heal OR Healing OR aid OR help OR relief OR wellness OR support OR program OR software OR system OR device OR technique)) AND ("Document Title":"Clinical Trial" OR "Clinical Trials" OR "Randomized Controlled Trial" OR "Randomized Controlled Trials" OR RCT)) OR ((("Abstract":Stroke OR CVA OR Cerebrovascular OR Poststroke OR "post stroke" OR Hemiplegia OR paretic OR apoplexy OR paresis OR hemiparetic OR hemiparesis OR Neuroplasticity) OR (("Abstract":cerebral OR Brain OR cerebellar) AND ("Abstract":infarction OR ischemic OR thrombo* OR embolic OR attack OR vascular OR injury or injuries)) OR (("Abstract":cerebral OR Brain OR cerebella) AND ("Abstract":haemorrhage OR hemorrhage OR haematoma OR hematoma OR bleeding)) OR ("Abstract":"Transient ischemic attack" OR TIA OR "Subarachnoid hemorrhage" OR "Gait disorder")) AND (("Abstract":Computer OR assistant OR “Computer assisted” OR simulator OR simulation OR simulate OR “computer based” OR generate OR generation OR cyber OR virtual OR immersive OR immersion OR "semi immersive" OR metaverse OR 3D OR augmented OR digital OR visual OR ”virtual reality” OR VR OR ”augmented reality” OR AR OR ”Mixed reality” OR game OR games OR ”Video game” OR “interactive game" OR “interactive games" OR “Serious game" OR “Serious games" OR Gamification)) AND (("Abstract":Rehabilitation OR Rehab OR recovery OR restore OR restoration OR Habilitate OR Habilitation OR therapy OR treat OR treatment OR care OR plan OR intervention OR approach OR interaction OR Counseling or Heal OR Healing OR aid OR help OR relief OR support OR wellness OR program OR software OR system OR device OR technique)) AND ("Abstract":"Clinical Trial" OR "Clinical Trials" OR "Randomized Controlled Trial" OR "Randomized Controlled Trials" OR RCT)) OR ((("Keywords":Stroke OR CVA OR Cerebrovascular OR Poststroke OR "post stroke" OR Hemiplegia OR paretic OR apoplexy OR paresis OR hemiparetic OR hemiparesis OR Neuroplasticity) OR (("Keywords":cerebral OR Brain OR cerebellar) AND ("Keywords":infarction OR ischemic OR thrombo* OR embolic OR attack OR vascular OR injury or injuries)) OR (("Keywords":cerebral OR Brain OR cerebella) AND ("Keywords":haemorrhage OR hemorrhage OR haematoma OR hematoma OR bleeding)) OR ("Keywords":"Transient ischemic attack" OR TIA OR "Subarachnoid hemorrhage" OR "Gait disorder")) AND (("Keywords":Computer OR assistant OR “Computer assisted” OR simulator OR simulation OR simulate OR “computer based” OR generate OR generation OR cyber OR virtual OR immersive OR immersion OR "semi immersive" OR metaverse OR 3D OR augmented OR digital OR visual OR ”virtual reality” OR VR OR ”augmented reality” OR AR OR ”Mixed reality” OR game OR games OR ”Video game” OR “interactive game" OR “interactive games" OR “Serious game" OR “Serious games" OR Gamification)) AND (("Keywords":Rehabilitation OR Rehab OR recovery OR restore OR restoration OR Habilitate OR Habilitation OR therapy OR treat OR treatment OR care OR plan OR intervention OR approach OR interaction OR Counseling or Heal OR Healing OR aid OR help OR relief OR support OR wellness OR program OR software OR system OR device OR technique)) AND ("Keywords":"Clinical Trial" OR "Clinical Trials" OR "Randomized Controlled Trial" OR "Randomized Controlled Trials" OR RCT))

**1.4. Web of Science search strategy**

TS=((((Stroke*) OR (CVA) OR (Cerebrovas*) OR (Poststroke*) OR (post-stroke*) OR (Hemipleg*) OR (paretic) OR (apoplexy) OR (paresis) OR (Hemipare*) OR (Neuroplast*)) OR (((cerebral*) OR (Brain*) OR (cerebellar)) AND ((infarct*) OR (isch?mi*) OR (thrombo*) OR (emboli*) OR (apoplex*) OR (attack*) OR (vascular*) OR (injur*))) OR (((cerebral) OR (Brain) OR (cerebella)) AND ((haemorrhage) OR (hemorrhage) OR (haematoma) OR (hematoma) OR (bleed*))) OR (“Transient ischemic attack”) OR (TIA) OR (“Subarachnoid hemorrhage”) OR (“Gait Disorders”)) AND (((Comput*) OR (asisst*) OR (Comput* assist*) OR (simulat*) OR (comput* base*) OR (genera*) OR (cyber*) OR (“virtual*”) OR (immers*) OR ("semi* immers*") OR (metaverse*) OR (3D) OR (augment*) OR (digit*) OR (visual*) OR (“virtual reality”) OR (virtual reality) OR (“VR”) OR (“augmented reality”) OR (augmented reality) OR (“AR”) OR (“Mixed reality”) OR (Mixed reality) OR ("game*") OR (“Video* game*”) OR (Video game) OR (interact* game*") OR (Serious* game*") OR (Gamificat*))) AND ((Rehab*) OR (recovery) OR (restore*) OR (Habilitat*) OR (therapy) OR (treat*) OR (care) OR (plan*) OR (intervent*) OR (approach*) OR (interact*) OR (Counsel*) or (Heal*) OR (aid*) OR (help*) OR (relief*) OR (well*) OR (support) OR (program) OR (software) OR (system) OR (device) OR (technique*)) AND ((“Clinical Trial*”) OR (“Random* Control* Trial*”) OR (“RCT”)))

**1.5. Psycnet search strategy:**

(((Title: Stroke* OR Title: CVA OR Title: Cerebrovascular OR Title: Poststroke OR Title: post stroke OR Title: Hemiplegia OR Title: paretic OR Title: apoplexy OR Title: paresis OR Title: hemiparetic OR Title: hemiparesis OR Title: Neuroplasticity) OR ((Title: cerebral OR Title: Brain OR Title: cerebellar) AND (Title: infarct* OR Title: ischem* OR Title: thrombo* OR Title: embol* OR Title: attack OR Title: vascular OR Title: injur*)) OR ((Title: cerebral OR Title: Brain OR Title: cerebella) AND (Title: haemorrhage OR Title: hemorrhage OR Title: haematoma OR Title: hematoma OR Title: bleed*)) OR (Title: Transient ischemic attack OR Title: TIA OR Title: Subarachnoid hemorrhage OR Title: Gait disorder)) AND (Title: Computer OR Title: assistant OR Title: Computer assisted OR Title: simulat* OR Title: computer based OR Title: generat* OR Title: cyber* OR Title: virtual* OR Title: immers* OR Title: semi immersive OR Title: metaverse* OR Title: 3D OR Title: augmented OR Title: digital OR Title: visual* OR Title: virtual reality OR Title: VR OR Title: augmented reality OR Title: AR OR Title: Mixed reality OR Title: game* OR Title: Video game* OR Title: interactive game* OR Title: Serious game* OR Title: Gamification) AND (Title: Rehab* OR Title: recovery OR Title: restore* OR Title: Habilitat* OR Title: therapy OR Title: treat* OR Title: care OR Title: plan* OR Title: intervent* OR Title: approach OR Title: interact* OR Title: Counseling or Title: Heal* OR Title: aid* OR Title: help* OR Title: relief* OR Title: wellness OR Title: support OR Title: program OR Title: software OR Title: system OR Title: device OR Title: technique*) AND (Title: Clinical Trial* OR Title: Random* Control* Trial* OR Title: RCT)) OR (((abstract: Stroke* OR abstract: CVA OR abstract: Cerebrovascular OR abstract: Poststroke OR abstract: post stroke OR abstract: Hemiplegia OR abstract: paretic OR abstract: apoplexy OR abstract: paresis OR abstract: hemiparetic OR abstract: hemiparesis OR abstract: Neuroplasticity) OR ((abstract: cerebral OR abstract: Brain OR abstract: cerebellar) AND (abstract: infarct* OR abstract: ischem* OR abstract: thrombo* OR abstract: embol* OR abstract: attack OR abstract: vascular OR abstract: injur*)) OR ((abstract: cerebral OR abstract: Brain OR abstract: cerebella) AND (abstract: haemorrhage OR abstract: hemorrhage OR abstract: haematoma OR abstract: hematoma OR abstract: bleed*)) OR (abstract: Transient ischemic attack OR abstract: TIA OR abstract: Subarachnoid hemorrhage OR abstract: Gait disorder)) AND (abstract: Computer OR abstract: assistant OR abstract: Computer assisted OR abstract: simulat* OR abstract: computer based OR abstract: generat* OR abstract: cyber* OR abstract: virtual* OR abstract: immers* OR abstract: semi immersive OR abstract: metaverse* OR abstract: 3D OR abstract: augmented OR abstract: digital OR abstract: visual* OR abstract: virtual reality OR abstract: VR OR abstract: augmented reality OR abstract: AR OR abstract: Mixed reality OR abstract: game* OR abstract: Video game* OR abstract: interactive game* OR abstract: Serious game* OR abstract: Gamification) AND (abstract: Rehab* OR abstract: recovery OR abstract: restore* OR abstract: Habilitat* OR abstract: therapy OR abstract: treat* OR abstract: care OR abstract: plan* OR abstract: intervent* OR abstract: approach OR abstract: interact* OR abstract: Counseling or abstract: Heal* OR abstract: aid* OR abstract: help* OR abstract: relief* OR abstract: wellness OR abstract: support OR abstract: program OR abstract: software OR abstract: system OR abstract: device OR abstract: technique*) AND (abstract: Clinical Trial* OR abstract: Random* Control* Trial* OR abstract: RCT)) OR (((Keywords: Stroke* OR Keywords: CVA OR Keywords: Cerebrovascular OR Keywords: Poststroke OR Keywords: post stroke OR Keywords: Hemiplegia OR Keywords: paretic OR Keywords: apoplexy OR Keywords: paresis OR Keywords: hemiparetic OR Keywords: hemiparesis OR Keywords: Neuroplasticity) OR ((Keywords: cerebral OR Keywords: Brain OR Keywords: cerebellar) AND (Keywords: infarct* OR Keywords: ischem* OR Keywords: thrombo* OR Keywords: embol* OR Keywords: attack OR Keywords: vascular OR Keywords: injur*)) OR ((Keywords: cerebral OR Keywords: Brain OR Keywords: cerebella) AND (Keywords: haemorrhage OR Keywords: hemorrhage OR Keywords: haematoma OR Keywords: hematoma OR Keywords: bleed*)) OR (Keywords: Transient ischemic attack OR Keywords: TIA OR Keywords: Subarachnoid hemorrhage OR Keywords: Gait disorder)) AND (Keywords: Computer OR Keywords: assistant OR Keywords: Computer assisted OR Keywords: simulat* OR Keywords: computer based OR Keywords: generat* OR Keywords: cyber* OR Keywords: virtual* OR Keywords: immers* OR Keywords: semi immersive OR Keywords: metaverse* OR Keywords: 3D OR Keywords: augmented OR Keywords: digital OR Keywords: visual* OR Keywords: virtual reality OR Keywords: VR OR Keywords: augmented reality OR Keywords: AR OR Keywords: Mixed reality OR Keywords: game* OR Keywords: Video game* OR Keywords: interactive game* OR Keywords: Serious game* OR Keywords: Gamification) AND (Keywords: Rehab* OR Keywords: recovery OR Keywords: restore* OR Keywords: Habilitat* OR Keywords: therapy OR Keywords: treat* OR Keywords: care OR Keywords: plan* OR Keywords: intervent* OR Keywords: approach OR Keywords: interact* OR Keywords: Counseling or Keywords: Heal* OR Keywords: aid* OR Keywords: help* OR Keywords: relief* OR Keywords: wellness OR Keywords: support OR Keywords: program OR Keywords: software OR Keywords: system OR Keywords: device OR Keywords: technique*) AND (Keywords: Clinical Trial* OR Keywords: Random* Control* Trial* OR Keywords: RCT))
