# Supplementary material for: The efficacy of virtual reality for upper limb rehabilitation in stroke patients: a systematic review and meta-analysis
Source: BMC Med Inform Decis Mak. 2024 May 24;24:135. doi: 10.1186/s12911-024-02534-y (PMC11127427; doi:10.1186/s12911-024-02534-y)
Supplement: Supplementary file 3 — Supplementary Material 3 [file 12911_2024_2534_MOESM3_ESM.docx]

**Additional File 3**

**Characteristics of included studies** [ordered by study ID]

**Table 1-** Characteristics of Study Number 1

| Study Title | Error Augmentation Enhancing Arm Recovery in Individuals With Chronic Stroke: A Randomized Crossover Design |
| --- | --- |
| Study ID | Abdollahi 2014 |
| VR type | Semi-immersive |
| Participants | 26 participants with chronic hemiparesis due to single cortical stroke at least 6 months prior  Inclusion criteria: proximal arm strength, persisting deficits, no severe sensory deficits, ataxia, spasticity, etc.  Mean age: 57.92 years  12 men, 14 women  Time since stroke: 6-259 weeks |
| Interventions | EG: Error augmentation training - amplified errors visually and haptically during repetitive practice  CG: Equivalent repetitive practice without error augmentation  45 min/session, 3 sessions/week, 2 weeks |
| Comparison | Error augmentation training vs standard repetitive practice |
| Outcomes | Arm motor impairment (Fugl-Meyer)  Functional ability (WMFT)  Dexterity (Box and Blocks)  Reachable workspace (ROM)  Motivation (Intrinsic Motivation Inventory) |
| Efficacy | Error augmentation training showed small improvements in arm motor impairment and function compared to standard practice. However, the study was limited by small sample size and short intervention period. |
| Challenges | Potential issues with setup time, arm support, patient fatigue, and compensatory movements. Outcome measures may not have been sensitive enough to detect changes. Lack of participant engagement due to repetitive training. |

**Table 2-** Characteristics of Study Number 2

| Study Title | Influence of New Technologies on Post-Stroke Rehabilitation: A Comparison of Armeo Spring to the Kinect System |
| --- | --- |
| Study ID | Adomavičienė 2019 |
| VR type | Semi-immersive |
| Participants | 42 patients with hemiparesis after ischemic or hemorrhagic stroke  Aged 60-74 years  5-12 weeks post-stroke  Mean age: 64.6 years  28 males, 14 females |
| Interventions | Experimental group (n=17): Conventional stroke rehabilitation plus 45 min/day of Armeo Spring robot-assisted upper limb training over 10 sessions  Control group (n=25): Conventional stroke rehabilitation plus 45 min/day of Kinect-based virtual reality upper limb training over 10 sessions |
| Comparison | Robot-assisted training versus virtual reality training |
| Outcomes | Functional independence (FIM)  Upper limb function: range of motion, muscle tone (MAS), dexterity (Box and Block test), grip strength  Cognitive function (MMSE, ACE-R)  Anxiety and depression (HADS) |
| Efficacy | The robot-assisted training group showed greater improvements in upper limb function, including increased functional independence, range of motion, grip strength and dexterity. They also had greater improvements in cognitive function and decreased anxiety levels. |
| Challenges | The virtual reality training group had increased muscle tone at the elbow and wrist which could limit recovery.  60 minutes of training was too long and patients became fatigued after 30 minutes.  Unstable seating, fall risks and compensatory movements during VR training could affect recovery.  Small sample size and short training duration (10 sessions) limit generalizability. |

**Table 3-** Characteristics of Study Number 3

| Study Title | Brain-computer interface-based robotic end effector system for wrist and hand rehabilitation: Results of a three-armed randomized controlled trial for chronic stroke |
| --- | --- |
| Study ID | Ang 2014 |
| VR type | none-immersive |
| Participants | 21 participants with chronic stroke (>4 months post-stroke)  Moderate to severe upper extremity impairment (FMMA 10-50)  Mean age: 54.2 years  14 males, 7 females |
| Interventions | Experimental group (n=6): EEG-based brain-computer interface coupled with haptic knob robot for hand grasping and wrist rotation training  Control group 1 (n=8): Haptic knob robot only  Control group 2 (n=7): Standard arm therapy |
| Comparison | EEG-based BCI + robot vs. robot only vs. standard arm therapy |
| Outcomes | Fugl-Meyer Motor Assessment (FMMA) at baseline, post-intervention, 6 weeks, and 18 weeks  Assessed arm motor ability and function |
| Efficacy | BCI + robot group improved FMMA scores from 33 to 40.2 at post-intervention, maintained gains at follow-ups  Distal and proximal arm function improved  Robot only and standard therapy groups had smaller FMMA gains that were not maintained |
| Challenges | Small sample size  Difficulty with recruitment  Lack of standardization of standard therapy  Ceiling effect of FMMA, focused on proximal arm |

**Table 4-** Characteristics of Study Number 4

| Study Title | Artificial Intelligence Limb Rehabilitation System on Account of Virtual Reality Technology on Long-Term Health Management of Stroke Patients in the Context of the Internet |
| --- | --- |
| Study ID | Bai 2022 |
| VR type | Semi-immersive |
| Participants | 50 patients with hemiplegia affecting upper or lower limbs following stroke were recruited from a hospital in China.  25 patients were allocated to the experimental group (EG) and 25 to the control group.  Mean age was 54.2 ± 13.7 years in the EG and 56.9 ± 14.6 years in the CG.  33 participants were male and 17 were female.  Average time since stroke onset was 141.54 ± 38.28 days in the EG and 132.29 ± 41.62 days in the CG. |
| Interventions | The EG received AI-based VR limb rehabilitation in addition to traditional rehabilitation. The VR system tracked motions and provided feedback during exercises. VR training was 40-60 minutes per day, 5 days per week for 10 weeks.  The CG received only traditional rehabilitation like physical, occupational, and speech therapy. |
| Comparison | The study compared an AI-based VR intervention plus traditional rehabilitation to traditional rehabilitation alone. |
| Outcomes | Upper and lower limb motor function, balance, walking speed, and ability to perform activities of daily living were measured at baseline and after the 10-week intervention using standardized assessments. |
| Efficacy | The EG showed significantly greater improvements than the CG in upper and lower limb motor function, balance, walking speed, and daily activity performance, demonstrating the efficacy of the AI-powered VR intervention. The system's ability to track progress, adjust difficulty, provide feedback, and increase patient engagement likely contributed to its benefits. |
| Challenges | Potential challenges include the expense of VR equipment, limited software/module variety currently, device safety concerns, and technical complexity of implementing the system. Further research on optimal protocols and improved accessible designs could help address these barriers. |

**Table 5-** Characteristics of Study Number 5

| Study Title | Domiciliary VR-Based Therapy for Functional Recovery and Cortical Reorganization: Randomized Controlled Trial in Participants at the Chronic Stage Post Stroke |
| --- | --- |
| Study ID | Ballester 2017 |
| VR type | Semi-immersive |
| Participants | Recruited from 10 stroke centers in Spain  35 total participants: 17 intervention, 18 control  Inclusion criteria: mild-to-moderate upper limb hemiparesis from ischemic or hemorrhagic stroke >12 months prior; able to manipulate Wii remote; MMSE >22  Exclusion criteria: severe comorbidities impairing participation; shoulder subluxation; pacemaker  Mean age: EG 65.05 years, CG 61.75 years  21 males, 14 females (9 EG, 12 CG)  89% ischemic stroke  Mean time since stroke onset: EG 1073 days, CG 798 days |
| Interventions | EG: 3 weeks home-based VR training using custom RGS system for ~20 min/day. Focused on reaching, grasping, placing of upper limbs.  CG: 3 weeks home-based occupational therapy involving functional unilateral and bilateral upper limb tasks for 20 min/day. |
| Comparison | VR immersive intervention compared to conventional occupational therapy |
| Outcomes | Assessed at baseline, 3 weeks, 12 weeks  Upper limb motor function, ADLs, tone, strength, pain, mood |
| Efficacy | The VR group demonstrated greater improvements in distal upper limb function, range of movement, and reduction in shoulder pain compared to controls. However, differences did not reach clinical impact. Improvements were not maintained at 12 week follow-up. The study provides evidence that VR therapy enhances cortical reorganization and motor recovery even at chronic stroke stages. |
| Challenges | Lack of responsiveness of UE-FM in chronic stroke patients  Stringent inclusion criteria limits generalizability  Differences between groups did not reach clinical significance  No retention of improvements at follow-up  RGS system restricted compensatory trunk movements  Conventional therapy may have lacked sufficient intensity |

**Table 6-** Characteristics of Study Number 6

| Study Title | Is upper limb virtual reality training more intensive than conventional training for patients in the subacute phase after stroke? An analysis of treatment intensity and content |
| --- | --- |
| Study ID | Brunner 2016 |
| VR type | Semi-immersive |
| Participants | 50 patients with impaired upper limb function after stroke (28 male, 22 female)  Inclusion criteria: 3 months post-stroke, MMSE score >20, ARAT score <52, active shoulder extension/abduction ≥20 degrees  Mean age: 60.6 years  Mean days post-stroke: VR group 50.9 days, conventional group 48.6 days |
| Interventions | VR group (n=25): YouGrabber VR system with wearable gloves, 45-60 min sessions 4-5x/week for 4 weeks  Conventional group (n=25): Task-oriented training on gross movements, dexterity, strength, stretching, ADLs, 45-60 min sessions 4-5x/week for 4 weeks |
| Comparison | VR training vs conventional upper limb training |
| Outcomes | Activity rate, active training time, total training time, training content, repetitions (video analysis)  Assessed at baseline and after 4 weeks of intervention |
| Efficacy | The VR training resulted in superior treatment intensity compared to conventional training, demonstrated by a significantly higher activity rate (77.6% vs 67.3%), longer active training time for patients with severe paresis (35.7 min vs 24.1 min), and longer total training time for patients with severe paresis (45.6 min vs 35.1 min). The VR system provided more functional task practice (100% vs 65% functional tasks) and multimodal feedback compared to conventional training. |
| Challenges | Challenges It can be challenging for therapists to continually engage patients and maintain active training when fatigue sets in. The VR system helped address this by gamifying the exercises and providing instant feedback to re-engage patients. However, counting repetitions provided limited added value. More research is needed on tailoring VR parameters and feedback systems to individual patient capabilities and goals. |

**Table 7-** Characteristics of Study Number 7

| Study Title | Virtual Reality Training for Upper Extremity in Subacute Stroke (VIRTUES) |
| --- | --- |
| Study ID | Brunner 2017 |
| VR type | Semi-immersive |
| Participants | 120 participants with upper extremity motor impairment within 12 weeks after ischemic or hemorrhagic stroke  Mean age 23-89 years  77 male, 43 female  Inclusion criteria: first-ever stroke or previous stroke without lasting impairment, impaired arm function, able to actively flex and abduct shoulder  Exclusion criteria: severe comorbidity, shoulder subluxation, pacemaker |
| Interventions | Experimental group (n=62): 16 sessions over 4 weeks of VR training using YouGrabber system with games and exercises  Control group (n=58): 16 sessions over 4 weeks of time-matched conventional arm exercises |
| Comparison | VR training compared to conventional upper extremity exercises |
| Outcomes | Assessed at baseline, post-intervention, and 3-month follow-up  Action Research Arm Test, Box and Blocks Test, Functional Independence Measure, ABILHAND, Patient Global Impression of Change |
| Efficacy | Both groups showed significant within-group improvement on all outcomes from baseline to post-intervention and 3-month follow-up  No significant between-group differences in improvement were found  The addition of VR training was not superior to conventional training in the subacute stroke phase |
| Challenges | The semi-immersive VR system may not have provided a real enough experience to elicit greater gains  Spontaneous biological recovery in the subacute phase may have masked potential differences between groups  A third study arm with increased VR training time may have shown greater effects |

**Table 8-** Characteristics of Study Number 8

| Study Title | Robotic Assisted Upper Limb Training Post Stroke: A Randomized Control Trial Using Combinatory Approach Toward Reducing Workforce Demands |
| --- | --- |
| Study ID | Budhota 2021 |
| VR type | none-immersive |
| Participants | Patients with first-ever clinical stroke with predominant arm motor deficits (n=44)  Moderate to severe arm paresis, Fugl-Meyer Assessment (FMA) score ≤40  Time since stroke onset 3-24 months  Mean age 55.46 years  25 males, 19 females |
| Interventions | Experimental group (n=22): 60 min robotic therapy with H-Man device + 30 min conventional therapy, 3 times/week for 6 weeks  Control group (n=22): 90 min conventional therapy, 3 times/week for 6 weeks |
| Comparison | Robotic therapy + conventional therapy vs conventional therapy alone |
| Outcomes | Fugl-Meyer Assessment (FMA)  Action Research Arm Test (ARAT)  Grip strength (GS)  Kinematic measures from robot: smoothness, time to peak velocity, task error |
| Efficacy | Both groups showed significant improvements in FMA, ARAT, GS from baseline to end of training  Experimental group showed greater improvements in FMA and smoothness metrics compared to control group  Combinatory training allowed reducing conventional therapy time while achieving comparable outcomes |
| Challenges | Patients with very severe deficits (FMA <20) were excluded  Follow-up limited to 6 months  Sample slightly younger than typical stroke population  Did not assess global measures like quality of life  Some patients unable to initiate robotic assessment tasks due to weakness |

**Table 93-** Characteristics of Study Number 9

| Study Title | The efficacy of interactive, motion capture-based rehabilitation on functional outcomes in an inpatient stroke population: a randomized controlled trial |
| --- | --- |
| Study ID | Cannell 2017 |
| VR type | Semi-immersive |
| Participants | The study recruited 79 patients less than 6 months after stroke with reduced mobility and clinician-determined capacity to improve from an inpatient rehabilitation hospital in Australia.  There were 41 male and 38 female participants.  The mean age was 73.8 years.  The mean time since stroke onset was 19-26 days. |
| Interventions | The experimental group (n=39) received individualized repetitive exercises using virtual reality games targeting balance, strength, and function via the Jintronix Rehabilitation System. Sessions were up to 1 hour per day on weekdays.  The control group (n=40) received individualized functional retraining, strength, balance and endurance exercises prescribed by a physical therapist. This was delivered in group classes or 1:1 with a rehabilitation assistant, also up to 1 hour per day on weekdays. |
| Comparison | The virtual reality intervention was compared to conventional physical therapy. |
| Outcomes | Outcomes were assessed at baseline and after the interventions.  Quantitative metrics included tests of standing balance, sitting balance, upper limb function, dynamic standing balance, gait velocity, and functional mobility |
| Efficacy | Both groups demonstrated improvements in balance, upper limb function, mobility and gait velocity from baseline to post-intervention. The virtual reality group improved slightly more in some metrics like comfortable gait velocity. However, there were no statistically significant differences between the groups. The variability in functional levels at baseline and natural recovery early after stroke likely contributed to the lack of significant between-group differences. |
| Challenges | The heterogeneity of functional levels at study entry made it difficult to detect statistical differences between groups.  Natural recovery early after stroke may obscure differences between interventions.  Short hospital stays and low number of therapy sessions were limitations.  Lack of long-term follow up data.  The sample size was inadequate to demonstrate non-inferiority between groups. |

**Table 10-** Characteristics of Study Number 10

| Study Title | The Effect of Virtual Reality on Motor Anticipation and Hand Function in Patients with Subacute Stroke: A Randomized Trial on Movement-Related Potential |
| --- | --- |
| Study ID | Chen 2022 |
| VR type | none-immersive |
| Participants | 36 patients with upper limb hemiparesis from first-time stroke within 1-3 months prior  Inclusion criteria: minimum 20° wrist and 10° finger flexion/extension in paretic limb  Age range: 40-80 years old  20 males, 16 females  Mean age: 57.8 years in VR group, 58.4 years in control group |
| Interventions | VR group (n=18): Non-immersive VR system with passive arm exoskeleton, reaching/grasping games, 60 min sessions 5x/week for 2 weeks  Control group (n=18): Conventional occupational therapy focused on grip strength and ADLs, matched duration |
| Comparison | Non-immersive VR training versus conventional occupational therapy |
| Outcomes | Reaction time, movement-related cortical potentials  Clinical scales: UL-FMA, ARAT, NIHSS  Assessed at baseline and after 2 weeks training |
| Efficacy | The VR group demonstrated significantly greater reductions in EMG reaction time and movement-related cortical potential latency and amplitude compared to controls.  The VR group also showed significantly greater gains in UL-FMA and ARAT scores, indicative of improved upper limb motor function.  These results suggest VR training may improve motor planning and reduce interhemispheric imbalance in subacute stroke patients. |
| Challenges | The narrow inclusion criteria limits generalization of results to only subacute stroke patients.  The wide participant age range also reduces generalizability of the findings.  Lack of follow-up assessments prevents determining any longer-term benefits of VR training. |

**Table 11-** Characteristics of Study Number 11

| Study Title | Effectiveness of upper-limb robotic-assisted therapy in the early rehabilitation phase after stroke: A single-blind, randomised, controlled trial |
| --- | --- |
| Study ID | Dehem 2019 |
| VR type | none-immersive |
| Participants | Recruited from 3 rehabilitation centers in Belgium  45 patients with acute stroke and upper limb motor impairment (Fugl Meyer Assessment Upper Extremity score <80%)  22 in control group, 23 in intervention group  Mean age: 67.9 years  22 males, 23 females  1 month post stroke onset |
| Interventions | Experimental group: Robotic-assisted therapy using REAplan robot, 4 sessions per week, 45 mins per session, over 9 weeks + conventional therapy  Control group: Conventional therapy focused on motor rehabilitation matched to needs over 9 weeks |
| Comparison | Robotic-assisted therapy vs conventional therapy |
| Outcomes | Assessed at baseline, 3 months, 6 months using:  Fugl Meyer Assessment Upper Extremity  Box and Block Test  Wolf Motor Function Test  Abilhand  Activlim  Stroke Impact Scale  Outcomes were upper limb motor control, manual dexterity, functional ability, ADLs, and participation |
| Efficacy | The robotic-assisted therapy group showed significantly greater improvements in gross manual dexterity, upper limb function, and social participation compared to conventional therapy. |
| Challenges | One patient in the robotic therapy group reported shoulder pain as an adverse event. |

**Table 12-** Characteristics of Study Number 12

| Study Title | Bimanual motor skill learning and robotic assistance for chronic hemiparetic stroke: a randomized controlled trial |
| --- | --- |
| Study ID | Doost 2020 |
| VR type | none-immersive |
| Participants | The study included 23 chronic stroke patients (at least 6 months post-stroke) with motor deficits in the upper limb. There were 12 males and 11 females.  The mean age of the stroke patients was 63.9 years.  20 patients had ischemic strokes and 3 had hemorrhagic strokes. They had varying levels of impairment based on NIHSS, mRS, and Abilhand scores. |
| Interventions | The stroke patients received training on a robotic rehabilitation device called REAplan. They performed a bimanual cooperative motor task called "Lift the Tray".  Patients were randomized to receive training in either active-assisted mode or active mode.  Each day they did 20 minutes of training broken into 15 blocks of 1 minute with breaks. |
| Comparison | The comparator group was 26 young, healthy controls with a mean age of 27.4 years and no neurological conditions.  The controls received the identical training protocol as the stroke group on the REAplan device. |
| Outcomes | The primary outcome was speed-accuracy tradeoff (SAT) before and after training.  Secondary outcomes included the Box and Blocks test for dexterity. |
| Efficacy | The stroke patients demonstrated learning of the bimanual skill, evidenced by improved SAT from baseline to day 2 in both training subgroups.  However, no generalization was seen to unimanual dexterity on the Box and Blocks test.  The active-assisted mode did not confer an advantage over the active mode for skill acquisition.  Some generalization of the bimanual skill to faster speeds was observed in the active subgroup. |
| Challenges | The sample size was small and the training duration was short, limiting functional gains.  Long-term retention and real-world outcomes were not examined.  The groups were not matched for age or impairment levels.  Active participation by patients is important for motor learning, but the active-assisted mode may reduce effort.  The robotic assistance was not customized and fixed protocols may not optimize learning in heterogeneous strokes. |

**Table 13-** Characteristics of Study Number 13

| Study Title | Combining Proprioceptive Neuromuscular Facilitation and Virtual Reality for Improving Sensorimotor Function in Stroke Survivors: A Randomized Clinical Trial |
| --- | --- |
| Study ID | Dos Santos 2019 |
| VR type | Semi-immersive |
| Participants | 40 participants with chronic stroke and hemiparesis  Mean age 55.6 years  23 men, 17 women |
| Interventions | PNF group (n=15): Proprioceptive neuromuscular facilitation therapy  VR group (n=11): Nintendo Wii games (semi-immersive VR)  PNF/VR group (n=14): Combination of PNF and VR |
| Comparison | No control group |
| Outcomes | Fugl-Meyer Assessment scale  Passive range of motion and pain  Upper limb motor function  Lower limb motor function  Balance  Sensorimotor function |
| Efficacy | All groups showed improvements in Fugl-Meyer and sensorimotor function  PNF and PNF/VR improved passive range of motion, reduced pain, and balance  VR improved lower limb motor function  No differences between groups |
| Challenges | Small sample size  No control group for comparison  Specific protocols may limit generalizability  Combining PNF and VR showed no added benefit over each alone |

**Table 14-** Characteristics of Study Number 14

| Study Title | The Effect of Virtual Reality-Based Therapy on Improving Upper Limb Functions in Individuals With Stroke: A Randomized Control Trial |
| --- | --- |
| Study ID | El-Kafy 2021 |
| VR type | Semi-immersive |
| Participants | 40 participants with chronic stroke (onset >6 months) and upper limb hemiparesis.  31 males, 9 females.  Mean age: 53.32 years in the experimental group, 54.46 years in the control group.  Inclusion criteria: aged 50-60 years, ischemic or hemorrhagic stroke >6 months prior, degree of spasticity in affected upper limb 1-2 on Modified Ashworth Scale.  Exclusion criteria: not reported.  Recruited from university physiotherapy department in Saudi Arabia. |
| Interventions | Experimental group: Conventional therapy (stretching, strengthening, proprioceptive exercises) plus virtual reality-based training using Armeo Spring robotic arm support with games.  Control group: Conventional therapy only.  Both groups received 45 minute sessions for 6 weeks. |
| Comparison | Virtual reality-based training plus conventional therapy compared to conventional therapy alone. |
| Outcomes | Assessed at baseline and after 6 weeks of intervention  Action Research Arm Test  Wolf Motor Function Test  Hand Grip Strength |
| Efficacy | The virtual reality-based training plus conventional therapy group showed significantly greater improvements in upper limb function on all outcome measures compared to conventional therapy alone. The addition of virtual reality-based training enhanced the benefits of conventional therapy. |
| Challenges | Small sample size from a single center limits generalizability.  Did not examine impact on functional activities like walking.  Effects were only measured short-term; longer follow up needed.  Recruitment challenges due to COVID-19 pandemic.  Did not fully implement ICF framework. |

**Table 15-** Characteristics of Study Number 15

| Study Title | Combined cognitive-motor rehabilitation in virtual reality improves motor outcomes in chronic stroke—A pilot study |
| --- | --- |
| Study ID | Faria 2018 |
| VR type | none-immersive |
| Participants | 24 chronic stroke patients (>6 months post-stroke) with cognitive and upper extremity motor deficits were recruited from rehabilitation units in Portugal.  15 males, 9 females  Mean age: 63 years  Experimental group: 57.1 years  Control group: 68.9 years  Inclusion criteria: ischemic or hemorrhagic stroke >6 months ago, arm weakness from stroke, able to manipulate VR controls  Exclusion criteria: severe comorbidities, shoulder subluxation, pacemaker |
| Interventions | Experimental group (n=12):  45 min sessions of VR-based cognitive-motor training (Reh@Task) plus conventional occupational therapy, 3 days/week for 4 weeks  Control group (n=12):  45 min sessions of conventional occupational therapy activities plus conventional occupational therapy, matched in time to experimental group |
| Comparison | VR-based cognitive-motor training plus conventional occupational therapy compared to conventional occupational therapy alone |
| Outcomes | Assessed at baseline, after 4 weeks intervention, and at 3 month follow-up  Cognitive function: Montreal Cognitive Assessment (MoCA)  Motor function: Fugl-Meyer Assessment (FM), Chedoke Arm and Hand Activity Inventory (CAHAI), Motricity Index (MI)  Spasticity: Modified Ashworth Scale (MAS)  Functional ability: Barthel Index (BI) |
| Efficacy | The VR-based training resulted in significantly greater improvements in arm motor function compared to conventional therapy alone as measured by the FM-Arm subscore (mean increase of 3.7 vs 0 points).  The VR group also showed clinically meaningful improvements in overall arm motor function, wrist/hand motor function, arm motor power, and cognitive function. |
| Challenges | Small sample size  Between-group differences at baseline  Lack of assessor blinding  Control group did not train identical competencies  Screening cognitive tests may lack sensitivity to detect changes |

**Table 16-** Characteristics of Study Number 16

| Study Title | Comparing integrated training of the hand and arm with isolated training of the same effectors in persons with stroke using haptically rendered virtual environments, a randomized clinical trial |
| --- | --- |
| Study ID | Fluet 2014 |
| VR type | Semi-immersive |
| Participants | 40 patients with chronic stroke (>6 months) and residual hemiparesis of the arm/hand  Inclusion criteria: at least 20° wrist extension, 10° finger extension  29 males, 11 females  Mean age: 54.6 years  Median time since stroke: 41.5-48.5 months  Moderate to mild stroke severity |
| Interventions | EG (n=20): Integrated training of hand and arm using virtual reality simulations  CG (n=20): Separate training of hand and arm using virtual reality simulations  15 hours over 2 weeks |
| Comparison | Integrated vs isolated training of hand and arm in virtual environments |
| Outcomes | Wolf Motor Function Test  Jebsen Test of Hand Function  Kinematic measures during virtual tasks |
| Efficacy | Both groups improved functionally based on WMFT and JTHF scores. The integrated training group demonstrated greater retention of improvements at 3 months follow-up. Both approaches were efficacious for improving coordination, speed, smoothness, and accuracy of movements. |
| Challenges | The study failed to detect significant between-group differences in activity level improvements. More research is needed to determine if an integrated approach provides additional benefits over isolated training of hand and arm. Generalizability may be limited due to small sample size and lack of statistical superiority between groups. |

**Table 17-** Characteristics of Study Number 17

| Study Title | A randomized clinical control study on the efficacy of three-dimensional upper limb robotic exoskeleton training in chronic stroke |
| --- | --- |
| Study ID | Frisoli 2022 |
| VR type | Semi-immersive |
| Participants | 22 chronic stroke patients with unilateral hemiparesis  11 in experimental group (EG), 11 in comparator group (CG)  Mean age: EG 62 years, CG 70 years  15 males, 7 females  30-37 months post-stroke onset |
| Interventions | EG: Robotic therapy with L-EXOS exoskeleton, reaching/manipulation exercises in VR, 45 min sessions, 3x/week for 6 weeks |
| Comparison | CG: Conventional manual therapy focused on reaching/grasping/manipulation tasks, 45 min sessions, 3x/week for 6 weeks |
| Outcomes | Fugl-Meyer Assessment (FMA), Modified Ashworth Scale (MAS), Bimanual Activity Test (BAT)  Assessed at baseline and after 6 weeks of intervention |
| Efficacy | EG had significantly greater improvements in FMA motor score (11.1 points) and proximal FMA (6.9 points) compared to CG (8.9 and 2.6 points, respectively)  EG had greater reduction in BAT time (4.8 sec) than CG (2.2 sec)  Results suggest robotic exoskeleton training more effective than conventional manual therapy for improving arm motor function in chronic stroke |
| Challenges | Small sample size limits generalizability  No follow-up assessment to determine long-term effects  Did not assess effects on activities of daily living and quality of life  Does not provide details on adverse events or safety |

**Table 18-** Characteristics of Study Number 18

| Study Title | Impact of a virtual reality program on post-stroke upper limb function: a randomized controlled trial |
| --- | --- |
| Study ID | Hegazy 2022 |
| VR type | Full-immersive |
| Participants | 10 patients who had a stroke within the past 6 months and mild upper limb spasticity, no cognitive impairments  Mean age: 55.3 years |
| Interventions | Experimental group (n=10): 15 minutes of VR training using "Super Punch" game with VR goggles and controllers, plus 1 hour of task-oriented upper limb training, 3 times per week for 6 weeks  Control group (n=10): 1 hour of task-oriented upper limb training, 3 times per week for 6 weeks |
| Comparison | VR training plus conventional therapy vs conventional therapy alone |
| Outcomes | Upper Extremity Functional Index (UEFI)  Grip strength  Assessed at baseline and after 6 weeks of intervention |
| Efficacy | The VR training group showed greater improvements in upper extremity function and grip strength compared to conventional therapy alone. UEFI scores increased by 12.6 points in the VR group versus 9.3 points in the control group. Grip strength increased by 11.1 kg in the VR group versus 3 kg in the control group. The results suggest adjunctive VR training enhances upper limb motor recovery after stroke. |
| Challenges | The VR game focused only on gross motor skills, not fine motor skills.  Potential issues with patient adaptation to VR and dropout.  Small sample size limits generalizability. |

**Table 19-** Characteristics of Study Number 19

| Study Title | Virtual Reality–Based Rehabilitation as a Feasible and Engaging Tool for the Management of Chronic Poststroke Upper-Extremity Function Recovery: Randomized Controlled Trial |
| --- | --- |
| Study ID | Hernandez 2022 |
| VR type | Semi-immersive |
| Participants | 51 participants with chronic stroke and residual mild to moderate upper extremity impairment (Fugl-Meyer Assessment scores 2-6)  37 males, 14 females  Median time since stroke: 5.3 years in intervention group, 4.4 years in control group  Mean age: 59.8 years in intervention group, 56.7 years in control group |
| Interventions | Experimental group (n=26): 4-week home-based upper extremity exercise program using Jintronix VR video game system with remote monitoring and adjustments by a therapist. Performed while standing or seated.  Control group (n=25): 4-week home exercise program using the Graded Repetitive Arm Supplementary Program (GRASP) manual without therapist supervision. |
| Comparison | VR-based intervention versus standard home exercise program without VR |
| Outcomes | Primary outcome: Fugl-Meyer Assessment for Upper Extremity (FMA-UE)  Secondary outcomes: Stroke Impact Scale (SIS), abridged Motor Activity Log (MAL)  Assessed at baseline and after 4-week intervention |
| Efficacy | The VR-based intervention group showed improvements in arm motor impairment (FMA-UE median increase from 30 to 36) and self-reported quality of life and arm use (SIS and MAL) from pre to post-intervention.  The control group also showed improvements, but to a lesser degree than the VR group.  The VR system provides an engaging way to perform repetitive upper extremity exercises and allows independent exercise with remote monitoring. |
| Challenges | Study limitations include small sample size and outcome measures that may lack sensitivity to change.  Long-term retention of upper limb gains was not maintained at follow-up.  Control group exercise time was not tracked.  No adverse effects reported. |

**Table 20-** Characteristics of Study Number 20

| Study Title | Comparison of Kinect2Scratch game-based training and therapist-based training for the improvement of upper extremity functions of patients with chronic stroke: a randomized controlled single-blinded trial |
| --- | --- |
| Study ID | Hung 2019 |
| VR type | Semi-immersive |
| Participants | 33 patients with chronic hemiplegic stroke  24 males, 9 females  Mean age 59 years  29-37.5 months post stroke onset  Inclusion criteria: unilateral lesions, active movement of proximal upper extremity, MMSE >20, age >18 years |
| Interventions | EG (n=17): 30 min Kinect2Scratch game training, 24 sessions over 12 weeks  CG (n=16): 30 min therapist-led training, 24 sessions over 12 weeks |
| Comparison | Kinect2Scratch game training vs. therapist-led training |
| Outcomes | Fugl-Meyer Assessment of Upper Extremity  Wolf Motor Function Test  Motor Activity Log  Pittsburgh Participation Scale  Accelerometer activity counts |
| Efficacy | The Kinect2Scratch game training showed similar improvements as therapist-led training in upper extremity function on the Fugl-Meyer and Wolf Motor Function Test. It also improved amount and quality of affected arm use on the Motor Activity Log compared to therapist-led training. Patients were more motivated and participated at higher rates during the Kinect2Scratch training. |
| Challenges | The total training dose may have been inadequate. No significant between-group differences were found. Findings cannot be generalized to patients without active proximal upper extremity movement. The study used a first generation Kinect sensor and 2D Scratch platform which is not fully immersive. Some patients experienced soreness after training. |

**Table 21-** Characteristics of Study Number 21

| Study Title | Wearable myoelectric interface enables high-dose, home-based training in severely impaired chronic stroke survivors |
| --- | --- |
| Study ID | Hung 2021 |
| VR type | Semi-immersive |
| Participants | 23 participants with moderate to severe upper limb impairment after chronic stroke (>6 months)  Mean age 58 years  10 men, 13 women |
| Interventions | Experimental group: Trained controlling cursor movements in a computer game using the MINT wearable device to measure arm muscle EMG signals. Did 90 min sessions, 5-6 days per week for 6 weeks.  Control group: Trained controlling cursor with single muscle activation at a time without reducing co-activation between muscles. Same schedule as experimental group. |
| Comparison | MINT training to reduce abnormal muscle co-activation vs training without reducing co-activation |
| Outcomes | Adherence, motivation, training performance, muscle co-activation  Assessed with training time, surveys, game metrics, EMG analysis |
| Efficacy | High adherence to home training (91%).  Reduced abnormal muscle co-activation by 54%.  Improved motivation and performance over time. |
| Challenges | Technical problems with software/hardware.  Fatigue with high training intensity.  Difficulty relaxing muscles or obtaining maximal contractions.  Skin irritation under electrodes.  High dropout rate for unrelated reasons.  Limitations in participation due to computer literacy or motivation. |

**Table 22-** Characteristics of Study Number 22

| Study Title | Virtual Reality Reflection Therapy Improves Motor Recovery and Motor Function in the Upper Extremities of People with Chronic Strok |
| --- | --- |
| Study ID | In Sung 2012 |
| VR type | none-immersive |
| Participants | 19 patients with chronic stroke (at least 6 months post-stroke) with upper extremity motor impairments  Recruited from not mentioned  11 in the experimental group, 8 in the control group  Mean age: 63.45 years in experimental group, 64.5 years in control group  11 males, 8 females |
| Interventions | Experimental group: Virtual Reality Reflection Therapy program for 30 minutes per day, 5 days per week for 4 weeks, plus conventional therapy. Exercises progressed in difficulty each week.  Control group: Sham program plus conventional therapy |
| Comparison | Virtual Reality Reflection Therapy vs sham program |
| Outcomes | Assessed before and after 4 weeks of intervention  Fugl-Meyer Assessment, Modified Ashworth Scale, Box and Block Test, Jebsen-Taylor Hand Function Test, Manual Function Test  Measured upper extremity motor recovery and motor function |
| Efficacy | The experimental group showed significant improvements in upper extremity motor recovery and motor function based on the Fugl-Meyer Assessment, Box and Block Test, Jebsen Hand Function Test, and Manual Function Test compared to the control group. This indicates that Virtual Reality Reflection Therapy was effective in improving outcomes for patients with chronic stroke. |
| Challenges | The study had a small sample size of only 19 patients.  Effects in patients with hemineglect need further study.  No side effects or disadvantages were reported. |

**Table 23-** Characteristics of Study Number 23

| Study Title | The Effects of Game-Based Breathing Exercise on Pulmonary Function in Stroke Patients: A Preliminary Study |
| --- | --- |
| Study ID | Joo 2015 |
| VR type | none-immersive |
| Participants | 19 stroke patients (12 men, 7 women) with reduced respiratory function due to hemiparesis  Mean age 55.05 ± 10.87 years  Recruited from a hospital in South Korea |
| Interventions | Game-based breathing exercise program using Breathing+ system  25 minutes, 3 days per week for 5 weeks  Performed seated in armchairs |
| Comparison | 19 stroke patients (10 men, 9 women) with reduced respiratory function  Mean age 56.73 ± 9.59 years  Received only conventional stroke rehabilitation exercises |
| Outcomes | Spirometry measures of FVC, FEV1, FEV1/FVC ratio, and MVV  Assessed respiratory muscle strength and lung volume |
| Efficacy | The game-based breathing exercise program was effective at improving measures of respiratory muscle strength and lung volume in stroke patients with impaired pulmonary function. The experimental group had significant improvements in FVC, FEV1, and MVV compared to the control group. |
| Challenges | Small sample size of only 19 patients per group limits generalizability  Lack of long-term follow-up, so unclear if benefits are sustained  No assessment of impact on quality of life or functional outcomes  Patients not blinded to treatment group |

**Table 24-** Characteristics of Study Number 24

| Study Title | Rehabilitation of the upper arm early after stroke: Video games versus conventional rehabilitation. A randomized controlled trial |
| --- | --- |
| Study ID | Laffont 2020 |
| VR type | none-immersive |
| Participants | 235 participants recruited from 10 stroke centers in the UK  Inclusion criteria: ischemic or hemorrhagic stroke within past 6 months, arm weakness from stroke (MRC scale power <5 in any joint plane), able to manipulate Wii remote control  Exclusion criteria: severe comorbidity impairing participation, symptomatic shoulder subluxation, pacemaker  117 participants in intervention group, 118 in control group  Mean age: intervention 66.8 years, control 68.0 years  56% men  89% ischemic stroke  Mean time post-stroke: intervention 57.3 days, control 56.3 days |
| Interventions | VR intervention: Therapists installed Wii and taught participants its use. Participants chose Wii sports games. Performed seated.  Control intervention: Participant-tailored arm exercises based on GRASP program, seated.  Both groups: 15 min warm-up then intervention for up to 45 min/day, 6 weeks |
| Comparison | VR full immersive intervention compared to conventional therapy |
| Outcomes | Assessed at baseline, 6 weeks, 6 months  Action Research Arm Test  Canadian Occupational Performance Measure  Stroke Impact Scale  Modified Rankin Scale  EQ5D Motor Activity Log Arm Function Test (6 months) |
| Efficacy | The VR immersive intervention showed greater improvements at 6 weeks in distal arm function per Fugl-Meyer Score (10.7 point increase vs 6.2 point increase with conventional therapy). The VR group also showed larger gains in grasping function per Box and Block Test (15.7 block increase vs 7.4 block increase). This suggests enhanced efficacy of VR for improving distal arm motor impairment and grasping after stroke. |
| Challenges | Small sample size may have limited ability to detect between-group differences. High Fugl-Meyer Score variability may have obscured potential VR benefits. Study did not assess patient/therapist opinions of VR games. VR system was non-immersive without advanced feedback features, which may have reduced potential benefits. Larger trials of more immersive/adaptable VR systems are needed. |

**Table 25-** Characteristics of Study Number 25

| Study Title | Bilateral movement-based computer games improve sensorimotor functions in subacute stroke survivors: a randomized controlled trial |
| --- | --- |
| Study ID | LAM 2022 |
| VR type | none-immersive |
| Participants | 93 subacute stroke survivors 1 week to 6 months post-stroke referred to a geriatric day hospital in Hong Kong within 1 week of hospital discharge  Mean age: 65.6 years  55 males, 38 females  Inclusion criteria: upper limb motor impairment after stroke  Exclusion criteria: not reported |
| Interventions | Experimental group (n=47): 30 minutes of bilateral movement-based computer games using a custom controller with handlebars, plus 3 hours conventional rehabilitation, 2 times per week for 8 weeks  Control group (n=46): 30 minutes of video-directed upper limb exercises, plus 3 hours conventional rehabilitation, 2 times per week for 8 weeks |
| Comparison | Bilateral movement-based computer games vs. video-directed exercises |
| Outcomes | Fugl-Meyer Assessment  Action Research Arm Test  Hand grip strength  SF-36 health survey  Assessed at baseline and 1 month follow-up |
| Efficacy | The experimental group showed significantly greater improvements in upper limb motor impairment and function compared to the control group, based on the Fugl-Meyer Assessment and Action Research Arm Test.  The experimental group also had earlier and greater gains in grip strength of the paretic hand.  The bilateral movement-based computer games appeared more beneficial for improving sensorimotor function when combined with conventional rehabilitation |
| Challenges | No major challenges or disadvantages were reported. The computer games were well-tolerated.  Longer follow-up is needed to determine if gains are sustained over time after the intervention ends. |

**Table 26-** Characteristics of Study Number 26

| Study Title | Effect of Robot-Assisted Game Training on Upper Extremity Function in Stroke Patients |
| --- | --- |
| Study ID | Lee 2017 |
| VR type | none-immersive |
| Participants | 50 total participants recruited from acute and subacute stroke patients at a hospital in South Korea  Inclusion criteria: hemiparesis after stroke affecting upper extremity function, at least 2/5 strength in shoulder and elbow  25 participants randomized to experimental group  25 participants randomized to control group  Mean days from stroke onset: EG 15.4 days, CG 14.4 days  Mean age: total 56.8 years, EG 55.8 years, CG 57.9 years  26 males total, EG 14, CG 12; 24 females total, EG 11, CG 13 |
| Interventions | EG: 30 minutes of robot-assisted upper extremity training using Neuro-X rehabilitation robot plus 30 minutes conventional therapy, 5 days/week for 2 weeks  CG: 60 minutes of conventional upper extremity therapy, 5 days/week for 2 weeks |
| Comparison | Robot-assisted upper extremity training vs conventional upper extremity therapy |
| Outcomes | Muscle strength (Manual Muscle Testing)  Upper extremity function (Manual Function Test)  Activities of daily living (Korean Modified Barthel Index)  Satisfaction questionnaire |
| Efficacy | Both groups demonstrated improvements in muscle strength, upper extremity function, and activities of daily living. The addition of robot-assisted training provided no significant differences or added benefit compared to conventional therapy alone in this small 2-week trial. |
| Challenges | Small sample size and short duration limit generalizability. No assessment of long-term retention of improvements. Advantages of robot-assisted training such as accuracy, interest, and motor learning potential require further study. |

**Table 27-** Characteristics of Study Number 27

| Study Title | Game-Based Virtual Reality Canoe Paddling Training to Improve Postural Balance and Upper Extremity Function: A Preliminary Randomized Controlled Study of 30 Patients with Subacute Stroke |
| --- | --- |
| Study ID | Lee 2018 |
| VR type | Semi-immersive |
| Participants | 30 patients with subacute stroke within the prior 6 months recruited from a hospital in South Korea  18 men, 12 women  Mean age: 61.8 years in experimental group, 61.33 years in control group |
| Interventions | Experimental group: VR training program using a Nintendo Wii Sports Resort game and canoe-like apparatus while seated. Participants performed paddling movements with both hands grasping a motion controller inserted into a canoe paddle accessory. Received conventional physical and occupational therapy in addition to VR training.  Control group: Conventional physical and occupational therapy only. |
| Comparison | VR training program plus conventional therapy versus conventional therapy alone. |
| Outcomes | Manual function test (MFT) to assess upper extremity motor function  Experimental group had greater improvement in MFT scores (2.47 point increase) compared to control group (1.47 point increase) |
| Efficacy | The VR training program resulted in greater improvement in upper extremity function compared to conventional therapy alone in patients with subacute stroke. The paddling movements and rewarding/entertaining experience of the VR program enhanced outcomes. |
| Challenges | Intensity of conventional therapy was not controlled between groups. Control group did not receive placebo VR therapy. Long-term effects were not evaluated. Assessor was likely not blinded to group allocation. Some patients experienced shoulder pain. |

**Table 28-** Characteristics of Study Number 28

| Study Title | Effect of Virtual Reality-based Bilateral Upper Extremity Training on Upper Extremity Function after Stroke: A Randomized Controlled Clinical Trial |
| --- | --- |
| Study ID | Lee 2016 |
| VR type | semi-immersive |
| Participants | Recruited from 10 stroke centers in the UK  235 participants total: 117 intervention, 118 control  Inclusion criteria: ischemic or hemorrhagic stroke within past 6 months, arm weakness from stroke (MRC Scale power <5 in any joint plane), able to manipulate Wii remote control  Exclusion criteria: severe comorbidity impairing participation, symptomatic shoulder subluxation, pacemaker  Mean age: intervention 66.8 years, control 68.0 years  56% male  89% ischemic stroke  Mean time post-stroke: intervention 57.3 days, control 56.3 days |
| Interventions | VR intervention: Wii sports games, seated play, participants' choice of games, home installation and training  Control: participant-tailored arm exercises seated per GRASP program  Both groups: 15 min warm up then 45 min intervention per day, 6 weeks |
| Comparison | VR full immersion intervention compared to conventional therapy |
| Outcomes | Assessed at baseline, 6 weeks, and 6 months  Action Research Arm Test  Canadian Occupational Performance Measure  Stroke Impact Scale  Modified Rankin Scale  EQ5D  Motor Activity Log Arm Function Test (6 months) |
| Efficacy | The VR immersive intervention demonstrated significant improvements in upper extremity function, dexterity, strength, and control compared to conventional therapy, with engagement and motivation provided by the VR system. The customized bilateral training allowed for progressive rehabilitation. |
| Challenges | Challenges Limitations include a small sample size lacking a control group receiving conventional therapy. The equipment was restricted to two adjustable angles (0 and 45 degrees), limiting motion. Further studies with larger sample sizes, proper control groups, and more adjustable equipment may provide additional insights. |

**Table 29-** Characteristics of Study Number 29

| Study Title | The Impact of Cognitive Function on Virtual Reality Intervention for Upper Extremity Rehabilitation of Patients With Subacute Stroke: Prospective Randomized Controlled Trial With 6-Month Follow-up |
| --- | --- |
| Study ID | Lo 2022 |
| VR type | none-immersive |
| Participants | 70 patients with first ever unilateral cerebral infarction confirmed by imaging, stroke onset <6 months prior  Inclusion criteria: residual upper extremity motor impairment  Exclusion criteria: not specified  31 in experimental group (VR + conventional therapy), 26 in control group (conventional therapy only)  25 M and 6 F in experimental group, 18 M and 8 F in control group  Mean age 59.2 years in both groups |
| Interventions | Experimental group: 30 min conventional therapy followed by 30 min VR training using Kinect games, 5 days/week for 3 weeks  Control group: 60 min conventional therapy, 5 days/week for 3 weeks |
| Comparison | VR training plus conventional therapy versus conventional therapy alone |
| Outcomes | Assessed at baseline, 3 weeks, 3 months and 6 months  Fugl-Meyer Assessment for Upper Extremity (FMA-UE)  Barthel Index (BI)  Instrumental Activities of Daily Living (IADL) scale |
| Efficacy | Both groups showed significant improvements in FMA-UE, BI, and IADL scores from baseline to 6 months  Experimental group showed faster improvements in IADL scores compared to control group, suggesting VR may provide added benefit for functional activities in those with cognitive impairment |
| Challenges | Kinect games not designed specifically for stroke rehabilitation, may underestimate potential benefits  Short 3 week intervention duration during hospitalization limits treatment intensity  Small sample size increases risk of type II error  2 patients reported fatigue, 1 reported shoulder pain with VR training |

**Table 30-** Characteristics of Study Number **30**

| Study Title | Virtual Reality Versus Conventional Treatment of Reaching Ability in Chronic Stroke: Clinical Feasibility Study |
| --- | --- |
| Study ID | Levin 2012 |
| VR type | semi-immersive |
| Participants | 6 patients with chronic hemiparesis after stroke in the VR intervention group  6 patients with chronic hemiparesis after stroke in the conventional therapy control group  Mean time since stroke onset was 2.6 years in VR group, 3.8 years in control group  Mean age was 58.1 years in VR group, 59.8 years in control group  50% male, 50% female in each group |
| Interventions | VR group: 45 min sessions, 3x/week for 3 weeks using virtual games and a virtual supermarket focused on reaching movements  Control group: 45 min sessions, 3x/week for 3 weeks of standard occupational therapy exercises for reaching movements |
| Comparison | VR intervention compared to conventional occupational therapy |
| Outcomes | Motor impairment (Fugl-Meyer Assessment, Composite Spasticity Index, Reaching Performance Scale)  Motor function (Box and Blocks Test, Wolf Motor Function Test)  Arm use in daily activities (Motor Activity Log)  Assessed at baseline, post-intervention, and 3-month follow-up |
| Efficacy | The VR intervention showed greater improvements in arm motor function and activity level compared to conventional therapy, based on the Fugl-Meyer Assessment and Wolf Motor Function Test results. The VR group also had earlier onset of improvements. However, no significant between-group differences were found for arm use in daily activities. |
| Challenges | No major challenges or disadvantages were reported for the VR intervention compared to conventional therapy in this small feasibility study. Larger trials are needed to further evaluate efficacy and identify any potential barriers to adoption. |

**Table 31-** Characteristics of Study Number 31

| Study Title | ystem on Patients with Stroke: Randomized Controlled Trial |
| --- | --- |
| Study ID | Li 2021 |
| VR type | none-immersive |
| Participants | Recruited from 10 stroke centers in China  30 participants total: 15 intervention, 15 control  Inclusion criteria: 20-70 years old, ischemic or hemorrhagic stroke within past 6 months, hemiparesis of upper limb, Brunnstrom stage ≥3, MMSE ≥20  Mean age: 62 years  19 males, 11 females |
| Interventions | Experimental group: 30 minutes smartphone-based VR training (3 games) plus 30 minutes conventional occupational therapy per day  Control group: 60 minutes conventional occupational therapy per day  5 days per week for 2 weeks |
| Comparison | Smartphone-based VR training compared to conventional occupational therapy alone |
| Outcomes | Assessed at baseline and after 2 weeks  Upper limb motor function, muscle strength, ADLs, cognitive function |
| Efficacy | The smartphone-based VR training was more effective than conventional therapy alone at improving upper limb motor function and cognitive function in patients with subacute stroke, based on significantly greater improvements in Fugl-Meyer, Action Research Arm Test, Brunnstrom stage, and cognitive game scores. The low-cost, engaging intervention allows independent practice. |
| Challenges | Limitations include small sample size, lack of blinding, limited glove sizes, and no patient experience with VR. Compensatory movements were not controlled. Further studies are needed to confirm results. |

**Table 32-** Characteristics of Study Number 32

| Study Title | Sensorimotor Rhythm-Brain Computer Interface With Audio-Cue, Motor Observation and Multisensory Feedback for Upper-Limb Stroke Rehabilitation: A Controlled Study |
| --- | --- |
| Study ID | Li 2022 |
| VR type | none-immersive |
| Participants | 24 patients with moderate to severe upper limb motor deficits after stroke (Brunnstrom hand score < IV)  6-24 weeks post-stroke  Mean age: 49.5 years  100% male |
| Interventions | Experimental group (n=12): 1 hour BCI training with motor imagery, audio/video cueing, robotic feedback + 2 hours conventional therapy for 10 sessions over 2 weeks  Control group (n=12): 3 hours conventional therapy only for 10 sessions over 2 weeks |
| Comparison | BCI-enhanced therapy vs. conventional therapy alone |
| Outcomes | Fugl-Meyer Assessment-Upper Extremity (FMA-UE)  Wolf Motor Function Test (WMFT)  Modified Barthel Index (MBI)  Assessed at baseline, 2 weeks, and 4 weeks |
| Efficacy | The BCI group showed significantly greater improvements in FMA-UE and WMFT at 4 weeks compared to controls.  100% of BCI group achieved clinically meaningful improvements on FMA-UE and WMFT vs. 58% and 67% of controls.  Suggests additional benefits of BCI training on upper limb function. |
| Challenges | Small sample size  Lack of EEG data from control group and at follow-up  Differences in age/stroke type between groups potentially impacting results |

**Table 33-** Characteristics of Study Number 33

| Study Title | A Comparative Efficacy Study of Robotic Priming of Bilateral Approach in Stroke Rehabilitation |
| --- | --- |
| Study ID | Li 2021 |
| VR type | none-immersive |
| Participants | 31 chronic stroke patients (≥3 months post-stroke)  Inclusion criteria: hemiparesis, impaired motor function and activity limitations in upper extremity, baseline Fugl-Meyer Assessment 16-53  19 men, 12 women  Mean age: 55.53 years  Health problem: hemiparesis after stroke |
| Interventions | Experimental group (n=15): 40-45 min bilateral robotic priming with Bi-Manu-Track robot + 40-45 min mirror therapy with functional tasks, 3x/week for 6 weeks  Comparator group (n=16): 40-45 min bilateral robotic priming with Bi-Manu-Track robot + 40-45 min bilateral upper limb training with functional tasks, 3x/week for 6 weeks |
| Comparison | Experimental group received mirror therapy  Comparator group received bilateral upper limb training |
| Outcomes | Motor impairment: Fugl-Meyer Assessment  Sensory impairment: Revised Nottingham Sensory Assessment  Bilateral arm function: Chedoke Arm and Hand Activity Inventory  Arm use: accelerometers  Assessed at baseline and after 6 weeks intervention |
| Efficacy | Experimental group showed significantly greater improvement in distal and total Fugl-Meyer Assessment scores and real-world arm use compared to comparator group.  Both groups demonstrated significant within-group improvements on most outcomes. |
| Challenges | Small sample size  Lack of follow-up evaluation  Did not consider effect of lesion location or cognitive impairments |

**Table 34-** Characteristics of Study Number 34

| Study Title | Novel Upper-Limb Rehabilitation System Based on Attention Technology for Post-Stroke Patients: A Preliminary Study |
| --- | --- |
| Study ID | Lin 2017 |
| VR type | none-immersive |
| Participants | 15 stroke patients with upper limb motor impairment affecting forearm, wrist, and hand movements  Matched between groups based on age, gender, time since stroke, affected side, and Fugl-Meyer score  No further details provided on demographics |
| Interventions | Experimental group (n=5): VR shooting game using motion tracking device and EEG headset, 3x/week for 4 weeks  Control group (n=10): VR shooting game using only motion tracking device, same schedule |
| Comparison | VR with EEG headset versus VR without EEG headset |
| Outcomes | Fugl-Meyer Assessment of Upper Extremity  Hit ratio and theta/beta ratio from EEG during VR game |
| Efficacy | The experimental group showed significantly improved Fugl-Meyer scores from pre- to post-training compared to controls.  Game performance also improved in the experimental group based on hit ratio and attention correlated with theta/beta ratio. |
| Challenges | Very small sample size limits generalizability  No long-term follow-up beyond 4 weeks  Lacked details on patient demographics and stroke characteristics |

**Table 35-** Characteristics of Study Number 35

| Study Title | The Frequency Effect of the Motor Imagery Brain Computer Interface Training on Cortical Response in Healthy Subjects: A Randomized Clinical Trial of Functional Near-Infrared Spectroscopy Study |
| --- | --- |
| Study ID | Lin 2022 |
| VR type | none-immersive |
| Participants | 16 healthy young subjects with no history of neurological disease, brain/upper limb trauma, or cognitive impairment.  Mean age: 22.94 years  11 females, 5 males |
| Interventions | Experimental group (n=8): Motor imagery based BCI training on non-dominant hand, 30 mins per day, 10 sessions total.  Control group (n=8): Same BCI training but only once every other day, 30 mins per session, 10 sessions total. |
| Comparison | Frequency of BCI training: Daily vs. every other day |
| Outcomes | Wolf Motor Function Test  BCI performance scores  fNIRS beta values in sensory motor cortex |
| Efficacy | The daily BCI training group showed increased cortical activation and improved BCI performance compared to every other day group.  Suggests higher frequency BCI training can maximize rehabilitation potential and improve outcomes in stroke patients. |
| Challenges | Only included healthy young subjects, future studies should examine effects in stroke patients.  Used only one type of BCI system, future studies should explore different systems. |

**Table 36-** Characteristics of Study Number 36

| Study Title | BCI-Based Rehabilitation on the Stroke in Sequela Stage |
| --- | --- |
| Study ID | Miao 2020 |
| VR type | semi-immersive |
| Participants | 16 patients with chronic stroke and upper limb motor impairment affecting the wrist/hand were recruited from a hospital in China.  12 were male, 4 were female.  Mean age was 46.6 years.  Time since stroke onset ranged from 6-38 months.  Inclusion criteria: ischemic, hemorrhagic stroke or trauma at least 6 months prior, motor impairment of wrist/hand.  Exclusion criteria: not specified. |
| Interventions | Experimental group (n=8): BCI-FES training plus routine rehabilitation. BCI system detected motor imagery through EEG and triggered FES to stimulate movement. Training was 12 sessions over 4 weeks.  Control group (n=8): Routine rehabilitation only including limb exercises 3 times per week for 4 weeks. No BCI-FES. |
| Comparison | BCI-FES augmented rehabilitation versus conventional rehabilitation alone. |
| Outcomes | Fugl-Meyer Assessment (FMA) scale quantified upper limb motor impairment before and after interventions. |
| Efficacy | The BCI-FES group showed a larger improvement in FMA score (3.5 point increase) compared to the control group (0.9 point increase), demonstrating the efficacy of BCI-FES training in improving motor function in chronic stroke affecting the hand/wrist.  The closed-loop feedback provided through the BCI-FES system appeared to enhance outcomes even years after stroke onset. |
| Challenges | Only half of the BCI-FES group showed FMA improvement, reasons for non-response require further study.  Some patients had poor BCI classification accuracy, which could limit efficacy.  The short 4-week training duration may restrict potential functional gains.  Maintaining patient motivation and EEG signal quality during repeat BCI sessions can be difficult.  Limited clinical accessibility of BCI systems remains a barrier to widespread adoption currently. |

**Table 37-** Characteristics of Study Number 37

| Study Title | Feasibility, Safety and Efficacy of a Virtual Reality Exergame System to Supplement Upper Extremity Rehabilitation Post-Stroke: A Pilot Randomized Clinical Trial and Proof of Principle |
| --- | --- |
| Study ID | Gheidar 2020 |
| VR type | Semi-immersive |
| Participants | 18 stroke survivors with mild to moderate upper extremity impairment in subacute or chronic stage, recruited from outpatient rehabilitation centers in Canada  Mean age: 49.9 years  10 males, 8 females |
| Interventions | Experimental group (n=9): Usual outpatient rehabilitation plus additional upper extremity training using Jintronix VR exergaming system, 30 mins 2-3 times per week for 4 weeks |
| Comparison | Control group (n=9): Usual outpatient rehabilitation only |
| Outcomes | Upper extremity motor function (Fugl-Meyer Assessment)  Manual dexterity (Box and Block Test)  Self-reported arm use (Motor Activity Log)  Health status/quality of life (Stroke Impact Scale) |
| Efficacy | The VR intervention led to significant improvements in self-reported quality of arm movement and mobility and physical domains of health status compared to control. However, it did not lead to significant differences in upper extremity motor function or manual dexterity. |
| Challenges | Small sample size limits generalizability  Participants could not be blinded  Excluded those with visual, cognitive or balance issues, limiting generalizability  Required therapist supervision |

**Table 38-** Characteristics of Study Number 38

| Study Title | Effect of Leap Motion-based 3D Immersive Virtual Reality Usage on Upper Extremity Function in Ischemic Stroke Patients |
| --- | --- |
| Study ID | ÖGÜN 2019 |
| VR type | full-immersive |
| Participants | 33 patients with ischemic stroke 6-24 months prior (VR group)  32 patients with ischemic stroke 6-24 months prior (control group)  Inclusion criteria: Brunnstrom stage ≥4 for upper extremity/hand  Mean age: 61.5 years (VR), 59.8 years (control)  51 male, 14 female |
| Interventions | VR group: 60 min immersive VR rehab using Leap Motion, 3x/week for 6 weeks  Control group: 45 min conventional exercises + 15 min sham VR, 3x/week for 6 weeks |
| Comparison | VR immersive intervention vs. conventional upper extremity exercises |
| Outcomes | Upper extremity function: Fugl-Meyer, Action Research Arm Test  Activities of daily living: Functional Independence Measure, Performance Assessment of Self-Care Skills |
| Efficacy | The VR group showed significantly greater improvement in upper extremity function (Fugl-Meyer, Action Research Arm Test) and self-care skills (Performance Assessment of Self-Care Skills) compared to the control group after the 6-week intervention. |
| Challenges | High dropout rate due to lack of motivation or difficulty using VR system  Time needed to adapt to VR system before starting rehabilitation  Possible shoulder fatigue with prolonged VR arm motions |

**Table 39-** Characteristics of Study Number 39

| Study Title | A comparison of the effects and usability of two exoskeletal robots with and without robotic actuation for upper extremity rehabilitation among patients with stroke: a single-blinded randomised controlled pilot study |
| --- | --- |
| Study ID | Park 2020 |
| VR type | none-immersive |
| Participants | 19 patients with upper extremity dysfunction due to ischemic or hemorrhagic stroke more than 3 months prior  Inclusion criteria: MRC scale scores of 3-4 for shoulder and elbow, Fugl-Meyer scores of 21-50  Mean age: 54.4 years  16 males, 3 females |
| Interventions | Experimental group (n=10): 30 min active-assistive robotic intervention with Armeo Power exoskeleton plus 30 min conventional therapy, 5 days/week for 4 weeks  Comparator group (n=9): 30 min passive robotic intervention with Armeo Spring exoskeleton plus 30 min conventional therapy, 5 days/week for 4 weeks |
| Comparison | Active-assistive robotic intervention vs passive robotic intervention |
| Outcomes | Wolf Motor Function Test (WMFT)  Fugl-Meyer Assessment (FMA)  Stroke Impact Scale (SIS)  Kinematic outcomes like spectral arc length and mean speed |
| Efficacy | The active-assistive robotic intervention showed improvements in upper limb function, motor impairment, strength, physical ability, and smoothness and speed of movements. The effects were greater than the passive robotic intervention. |
| Challenges | Conflict between robot assistance and voluntary movement/spasticity  Robot inertia hampered movement  May reduce active participation  Small sample size limits generalizability  Short intervention duration may not lead to lasting effects |

**Table 40-** Characteristics of Study Number 40

| Study Title | Effects of a Rehabilitation Program Using a Wearable Device on the Upper Limb Function, Performance of Activities of Daily Living, and Rehabilitation Participation in Patients with Acute Stroke |
| --- | --- |
| Study ID | Park 2021 |
| VR type | Semi-immersive |
| Participants | Recruited from 10 stroke centres in the UK,  235 participants: 117 intervention, 118 control,  Inclusion criteria: ischaemic or haemorrhagic stroke within the last 6 months, arm weakness owing to stroke, defined as MRC Scale power < 5 in any joint plane and able to manipulate the WiiTM remote control,  Exclusion criteria: severe comorbidity that could impair participation, symptomatic shoulder subluxation, or a pacemaker,  Mean (SD) age: intervention group 66.8 (14.6) years, control group 68.0 (11.9) years  56% men,  Stroke details: 89% ischaemic,  Timing post stroke: intervention group mean (SD) 57.3 (48.3) d, control group mean 56.3 (50.1) |
| Interventions | VR intervention: therapists visited the participants home and installed the Wii and taught participant show to use it. Participants were given the choice of any of the Wii sports games. Performed in a seated position  Control intervention: participant-tailored arm exercises (based on the GRASP program) in a seated position  Sessions: participants in both groups were instructed to warm up for 15 min and then perform the intervention for up to 45 min/d for 6 weeks |
| Comparison | VR full immersive intervention compared to conventional therapy |
| Outcomes | Outcomes were assessed at baseline, 6 weeks, and 6 months  Action Research Arm Test  Canadian Occupational Performance Measure  Stroke Impact Scale  Modified Rankin Scale  EQ5D  Motor Activity Log Arm Function Test (6 months) |
| Efficacy | The VR intervention group showed significantly greater improvements in hand strength, dexterity, and independence in daily activities compared to the control group. Both groups exhibited improved upper limb function, performance of activities of daily living, and rehabilitation participation. The results demonstrate good feasibility and applicability of the VR program using the wearable device. |
| Challenges | The study did not report any specific challenges or disadvantages of the VR intervention. Potential limitations could be cost and accessibility of the VR system, patient compliance with the home program, and technical difficulties operating the system independently. More research is needed on disadvantages and barriers to implementation. |

**Table 41-** Characteristics of Study Number 41

| Study Title | Exercises for paretic upper limb after stroke: a combined virtual-reality and telemedicine approach |
| --- | --- |
| Study ID | Piron 2009 |
| VR type | none-immersive |
| Participants | 36 patients with mild to moderate arm impairment after ischemic stroke  7-32 months post-stroke  Mean age 65.2 years  21 men, 15 women  Split into experimental (VR) and control (conventional therapy) groups with 18 patients each |
| Interventions | Experimental: 1 hour per day, 5 days per week for 4 weeks of VR-based telerehabilitation for the arm using motion tracking and virtual tasks  Control: 1 hour per day, 5 days per week for 4 weeks of traditional physical therapy exercises for the arm |
| Comparison | VR-based telerehabilitation versus conventional face-to-face physical therapy |
| Outcomes | Upper limb motor performance (Fugl-Meyer)  Spasticity (Ashworth scale)  Perceived manual ability (ABILHAND)  Assessed at baseline and after 4 weeks of intervention |
| Efficacy | The VR group showed significantly greater improvements in upper limb motor performance based on the Fugl-Meyer scale compared to the conventional therapy group.  Both groups showed reduced spasticity based on the Ashworth scale, with the VR group showing slightly greater reductions.  The VR approach was feasible and provided benefits remotely via telerehabilitation. |
| Challenges | The study did not mention any disadvantages or challenges of the VR intervention. Further research may be needed to identify potential limitations.  No adverse events or side effects were reported. VR appears to be safe based on this study. |

**Table 42-** Characteristics of Study Number 42

| Study Title | Comparison of 3D, Assist-as-Needed Robotic Arm/Hand Movement Training Provided with Pneu-WREX to Conventional Table Top Therapy Following Chronic Stroke |
| --- | --- |
| Study ID | Reinkensmeyer 2012 |
| VR type | none-immersive |
| Participants | 26 patients with chronic stroke and moderate to severe upper extremity weakness (Fugl-Meyer scores 10-35).  17 males, 9 females  Mean age 60 years  At least 3 months post-stroke |
| Interventions | Experimental group (n=13): 24 sessions of robot-assisted training with Pneu-WREX over 8-9 weeks. The robot provided assistance as needed during 3D functional tasks on screen.  Control group (n=13): 24 sessions of conventional tabletop training over 8-9 weeks supervised by a therapist, including stretching, strength training, ADL practice. |
| Comparison | Robot-assisted training vs conventional tabletop therapy |
| Outcomes | Arm motor impairment (Fugl-Meyer)  Functional ability (Rancho)  Self-reported arm use (Motor Activity Log)  Grip strength  Sensory function (Nottingham Sensory Assessment)  Manual dexterity (Box and Blocks) |
| Efficacy | The robot-assisted training group had greater improvements in arm motor impairment, grip strength, dexterity and sensory function compared to the conventional therapy group. However, the differences were small and may not be clinically meaningful. |
| Challenges | Small sample size, lack of blinding, multiple differences between the treatment arms. |

**Table 43-** Characteristics of Study Number 43

| Study Title | Proprioceptive Gaming: Making Finger Sensation Training Intense and Engaging with the P-Pong Game and PINKIE Robot |
| --- | --- |
| Study ID | Reinsdorf 2021 |
| VR type | Semi-immersive |
| Participants | 15 healthy participants with no known conditions (sample size not specified by gender)  Recruited at a university research laboratory in the United States |
| Interventions | Experimental group (n=8): 15 minutes of proprioceptive finger training using P-Pong game with PINKIE robot  Control group (n=7): 15 minutes of visual Pong game training without proprioceptive input |
| Comparison | Proprioceptive finger training vs visual training without proprioception |
| Outcomes | Finger proprioception accuracy assessed by Crisscross test  User engagement and motivation assessed by surveys |
| Efficacy | Proprioceptive training improved finger proprioception accuracy compared to visual training alone. Errors in the Crisscross test reduced by 2.2 mm after proprioceptive training.  Proprioceptive training led to higher user engagement and motivation based on survey results. |
| Challenges | Small sample size of 15 participants  Short duration of training and assessment  Conducted in healthy people so effects in stroke population need further evaluation |

**Table 44-** Characteristics of Study Number 44

| Study Title | Elements virtual rehabilitation improves motor, cognitive, and functional outcomes in adult stroke: evidence from a randomized controlled pilot study |
| --- | --- |
| Study ID | Rogers 2019 |
| VR type | Semi-immersive |
| Participants | 21 patients with upper extremity motor dysfunction resulting from recent stroke (8-62 days post-stroke)  9 males, 12 females  Mean age 64.5 years  Recruited from a hospital  Inclusion criteria: subacute stroke in past 4 months resulting in arm weakness  Exclusion criteria: not specified |
| Interventions | Experimental group (n=10): VR-based rehabilitation using a tabletop display and tangible interfaces (Elements system) along with usual therapy. 30-40 min sessions, 12 sessions over 4 weeks.  Control group (n=11): Usual occupational and physiotherapy focused on upper extremity, 3 hours daily |
| Comparison | VR-based rehabilitation vs conventional upper extremity rehabilitation |
| Outcomes | Assessed at baseline and post-intervention  Motor function: Box and Blocks Test  Cognition: Montreal Cognitive Assessment (MoCA), CogState computerized tests  Function: Neurobehavioral Functioning Inventory |
| Efficacy | The VR group showed significantly greater improvements in upper extremity motor function, overall cognitive status, executive function, and self-reported function compared to the control group.  Improvements were maintained at 1-month follow-up. |
| Challenges | Small sample size  Lack of blinding of outcome assessors  No long-term follow-up  High resource requirements for VR system may limit adoption |

**Table 45-** Characteristics of Study Number 45

| Study Title | Mirror Visual Feedback Prior to Robot-Assisted Training Facilitates Rehabilitation After Stroke: A Randomized Controlled Study |
| --- | --- |
| Study ID | Rong 2021 |
| VR type | none-immersive |
| Participants | 20 patients in the experimental group, 20 patients in the control group  All patients had subacute stroke (1-6 months post-stroke) with upper limb motor impairment  No details provided on sex, age, or specific health conditions |
| Interventions | Experimental group: 30 minutes of mirror visual feedback training followed by 60 minutes of robot-assisted upper limb training using the Armeo Power system, 1.5 hours per day, 5 days per week for 4 weeks  Control group: 30 minutes of sham mirror visual feedback followed by 60 minutes of robot-assisted upper limb training using the Armeo Power system on the same schedule |
| Comparison | Mirror visual feedback + robot assisted training vs. sham feedback + robot assisted training |
| Outcomes | Fugl-Meyer Assessment Upper Limb subscale (FMA-UL)  Functional Independence Measure (FIM)  Modified Barthel Index (MBI)  Grip strength |
| Efficacy | The addition of mirror visual feedback to robot-assisted upper limb training resulted in greater improvements in upper limb motor impairment (FMA-UL scores increased 15.6 points vs. 9.5 points), activities of daily living (FIM scores increased 15.39 points vs 12.6 points; MBI scores increased 13.25 points vs 10.9 points), and grip strength (increased 0.8 kg vs 0.51 kg) compared to robot training alone. |
| Challenges | No disadvantages, challenges, side effects, or safety concerns were reported in this study. Details were lacking on patient demographics and health conditions. The small sample size of 20 patients per group may limit generalizability of the results. |

**Table 46-** Characteristics of Study Number 46

| Study Title | Effects of upper limb robot-assisted therapy on motor recovery in subacute stroke patients |
| --- | --- |
| Study ID | Sale 2014 |
| VR type | none-immersive |
| Participants | 53 subacute stroke patients with unilateral upper limb motor impairment  30 ± 7 days post stroke onset  First-ever ischemic stroke  Able to follow instructions and sit upright  26 in experimental group, 27 in control group  Mean age 67.7 years in both groups  31 males, 22 females total |
| Interventions | Experimental group: 30 sessions of MIT-MANUS/InMotion2 robotic therapy for shoulder and elbow, 45 mins per session  Control group: 30 sessions of conventional physical therapy matched in duration, including stretching, exercises, functional tasks |
| Comparison | Robot-assisted therapy compared to conventional physical therapy |
| Outcomes | Assessed at baseline and after treatment using Fugl-Meyer, Modified Ashworth Scale, passive ROM, Motricity Index  Measured upper limb motor impairment, spasticity, range of motion, motor function |
| Efficacy | The robot-assisted therapy group showed significantly greater improvements in upper limb motor impairment, range of motion, and motor function compared to conventional therapy. Robot-assisted therapy also decreased shoulder and elbow spasticity, while conventional therapy did not. |
| Challenges | No specific challenges, limitations, side effects, or disadvantages of the robotic intervention were reported in the study. As it was conducted in a controlled rehabilitation setting, challenges with at-home use or accessibility may exist. The study sample was also relatively small. Further research on challenges and accessibility would be beneficial. |

**Table 47-** Characteristics of Study Number 47

| Study Title | Combining levodopa and virtual reality-based therapy for rehabilitation of the upper limb after acute stroke: pilot study Part II |
| --- | --- |
| Study ID | Samuel 2017 |
| VR type | Semi-immersive |
| Participants | Recruited from stroke centers in Singapore  8 participants total: 4 VR intervention, 4 control  Inclusion: ischemic stroke within past 21 days, upper limb hemiparesis  Exclusion: not stated  6 males, 2 females  Mean age: VR group 67.5 years, control group 52 years  Time from stroke onset: mean 8.7 days |
| Interventions | VR group: 30 min VR gaming targeting elbow flexion/extension + standard care  Control group: 30 min additional conventional occupational therapy + standard care  8-10 sessions over 2 weeks |
| Comparison | VR gaming vs additional conventional occupational therapy |
| Outcomes | Assessed at baseline and 2 weeks  Fugl-Meyer Upper Extremity scale  Action Research Arm Test  Kinematic measures |
| Efficacy | VR group had clinically significant improvements in FM-UE (16.5 points) and ARAT (15.3 points)  VR group showed improved quality of movement and range of motion  Patients enjoyed and engaged with VR intervention |
| Challenges | Small sample size  Control group had high function initially  Unable to control conventional therapy in control group  Short 2 week follow up  Sensor drift during measurements |

**Table 48-** Characteristics of Study Number 48

| Study Title | Arm Motor Recovery Using a Virtual Reality Intervention in Chronic Stroke: Randomized Control Trial |
| --- | --- |
| Study ID | Subramanian 2013 |
| VR type | Semi-immersive |
| Participants | 32 chronic stroke patients with mild to moderate upper limb impairment were recruited from rehabilitation centers.  Inclusion criteria: ischemic or hemorrhagic stroke at least 6 months prior, arm weakness (MRC <5), able to manipulate Wii remote.  Exclusion criteria: severe comorbidities, shoulder subluxation, pacemaker.  23 males, 9 females.  Mean age: 61 years.  Experimental group: 16 patients, mean age 62 years.  Control group: 16 patients, mean age 60 years. |
| Interventions | Experimental: reaching training in virtual supermarket on screen using own arm movements, 72 trials per session, 3 sessions per week for 4 weeks.  Control: reaching training using real targets on a board, matched sessions and trials to experimental group. |
| Comparison | Virtual reality training versus conventional physical training. |
| Outcomes | Kinematic analysis: endpoint velocity, joint ranges of motion, trunk displacement.  Clinical scales: Fugl-Meyer, Reaching Performance Scale, Wolf Motor Function Test, Motor Activity Log.  Assessed at baseline, post-test, and retention test. |
| Efficacy | The virtual reality training group showed increased endpoint velocity, shoulder joint ranges of motion, and real-world arm use after training and at retention. The physical training group also showed some improvements, but had increased trunk displacement compensation. The virtual reality training enabled improved upper limb movements without increasing compensatory trunk movements. |
| Challenges | No major challenges or disadvantages were reported for the virtual reality intervention. The virtual reality training was reported to be less stressful for patients during training compared to conventional methods. |

**Table 49-** Characteristics of Study Number 49

| Study Title | Robot-Assisted Training as Self-Training for Upper-Limb Hemiplegia in Chronic Stroke: A Randomized Controlled Trial |
| --- | --- |
| Study ID | Takebayashi 2022 |
| VR type | none-immersive |
| Participants | Patients with mild-to-moderate upper limb hemiparesis due to clinically first ever supratentorial stroke at least 6 months prior  Recruited from multiple sites in Japan  139 participants total: 44 in robotic training (RT) group, 43 in movement therapy (MT) group, 42 in control group  Mean age around 59-60 years in all groups  94 males, 35 females |
| Interventions | RT group: 40 min robotic training with ReoGo-J device + 20 min occupational therapy, 3x/week for 10 weeks  MT group: 40 min robotic training + 20 min constraint-induced movement therapy, 3x/week for 10 weeks  Control group: 40 min conventional exercises + 20 min occupational therapy, 3x/week for 10 weeks |
| Comparison | Robotic training vs movement therapy vs conventional rehabilitation |
| Outcomes | Primary outcome: Fugl-Meyer Assessment for upper extremity (FMA-UE)  Secondary outcomes: FMA subscores, Motor Activity Log-14, Action Research Arm Test, Motricity Index, Modified Ashworth Scale, range of motion, Stroke Impact Scale |
| Efficacy | The robotic training and movement therapy groups showed slightly greater improvements in FMA-UE and FMA subscores compared to control. All groups improved over time. |
| Challenges | Unable to blind patients to treatment allocation  More male participants may limit generalizability  Did not include all potential patients at sites  No measurement of repetition counts during therapies |

**Table 50-** Characteristics of Study Number 50

| Study Title | Impact of the robotic-assistance level on upper extremity function in stroke patients receiving adjunct robotic rehabilitation: sub-analysis of a randomized clinical trial |
| --- | --- |
| Study ID | Takebayashi 2022 |
| VR type | none-immersive |
| Participants | 30 patients with upper extremity hemiplegia due to stroke  Brunnstrom stage III or IV  4-8 weeks post-stroke  Mean age: 67.7 years  21 males, 9 females |
| Interventions | 40 minutes per day of robotic therapy using the ReoGo system, providing adjustable assistance to the shoulder, elbow, forearm and hand  Provided in addition to standard rehabilitation  6 weeks duration |
| Comparison | No comparator group |
| Outcomes | Fugl-Meyer Assessment (FMA)  Wolf Motor Function Test (WMFT), including performance time and functional ability  Measured upper extremity motor function, proximal function, performance time, and functional ability |
| Efficacy | This study found improved outcomes in upper extremity motor function, proximal function, performance time, and functional ability after 6 weeks of adjunct robotic therapy using the ReoGo system. The adjustable assistance provided by ReoGo appeared to facilitate repetitive practice and motor learning. However, the lack of a comparator group limits the ability to determine the efficacy of this intervention compared to standard care alone. |
| Challenges | The lack of a control group makes it difficult to determine the degree of added benefit from the robotic therapy. The small sample size may limit generalizability of the results. As participants received varying amounts of assistance from the robotic device, it is unclear the optimal assistance level for maximal functional gains. More research is needed on the dose-response relationship. |

**Table 51-** Characteristics of Study Number 51

| Study Title | Improving upper-limb and trunk kinematics by interactive gaming in individuals with chronic stroke: A single-blinded RCT |
| --- | --- |
| Study ID | Teremetz 2022 |
| VR type | none-immersive |
| Participants | 40 individuals with chronic stroke-related hemiparesis recruited from a university hospital in France.  Inclusion criteria: ischemic or hemorrhagic stroke onset >6 months prior; arm weakness from stroke defined as MRC Scale power <5 in any joint plane; able to manipulate Wii remote.  Exclusion criteria: severe comorbidities impairing participation; symptomatic shoulder subluxation; pacemaker.  24 males, 16 females; mean age 56 years; mean time since stroke 66.4 months. |
| Interventions | Experimental group (n=19): 12 sessions of Wii gaming (tennis, golf, boxing; 15 min each) over 4 weeks. Performed seated.  Comparator group (n=21): 12 sessions of conventional upper limb exercises over 4 weeks. Exercises were passive/active movements and task-oriented reaching/grasping. |
| Comparison | Non-immersive VR Wii intervention versus conventional upper limb exercises. |
| Outcomes | Primary outcomes: Change in elbow extension and forward trunk motion during reaching.  Secondary outcomes: Clinical measures (Fugl-Meyer, ARAT, Box and Block, MAL, SIS) and kinematic measures.  Assessed at baseline and post-intervention. |
| Efficacy | Both groups showed small improvements in elbow extension (EG: +4.5°, CG: +6.4°) and decreased forward trunk motion (EG: -3.3 cm, CG: -4.1 cm). No significant between-group differences. Minimal changes in clinical outcomes. Dose may have been too low for significant improvement. |
| Challenges | No long-term follow-up. Patients long time post-stroke. Same therapist performed both interventions. Lower satisfaction with Wii. |

**Table 52-** Characteristics of Study Number 52

| Study Title | ArmAssist Robotic System versus Matched Conventional Therapy for Poststroke Upper Limb Rehabilitation: A Randomized Clinical Trial |
| --- | --- |
| Study ID | Tomic 2017 |
| VR type | none-immersive |
| Participants | 26 subacute stroke patients with moderate to severe upper limb motor impairment (Fugl-Meyer Assessment upper extremity score ≤50)  21 males, 5 females  Mean age 57.4 ± 6.5 years  Hemiparesis from recent ischemic or hemorrhagic stroke  <3 months since stroke onset |
| Interventions | Experimental group (n=13): Conventional rehabilitation plus 30 minutes of ArmAssist robotic training, 5 days per week for 3 weeks (total 15 sessions). The ArmAssist facilitated planar shoulder and elbow movements to interact with computer games.  Control group (n=13): Conventional rehabilitation plus 30 minutes of additional conventional arm training matched in structure and amount to the ArmAssist training (reaching movements in the same plane). Also delivered for 5 days per week for 3 weeks (total 15 sessions). |
| Comparison | ArmAssist robotic training vs matched conventional upper limb training |
| Outcomes | Upper limb motor impairment (Fugl-Meyer Assessment Upper Extremity)  Upper limb function/activity (Wolf Motor Function Test - Functional Ability Scale)  Activities of daily living (Barthel Index) |
| Efficacy | The ArmAssist robotic training group had significantly greater improvements in upper limb motor impairment and function compared to the matched conventional training group. |
| Challenges | No challenges, disadvantages or side effects were reported in the study. |

**Table 53-** Characteristics of Study Number 53

| Study Title | Augmented reality for stroke rehabilitation during COVID-19 |
| --- | --- |
| Study ID | Yang 2022 |
| VR type | Semi-immersive |
| Participants | Recruited from stroke rehabilitation centres in Hong Kong  39 participants total: 16 in experimental group, 23 in comparator group  Inclusion criteria: Chronic stroke >3 years ago with motor impairments in upper limb, lower limb, and/or balance  Mean age: 64.61 years in experimental group, 62.79 years in comparator group  21 males, 18 females |
| Interventions | Experimental group: Augmented reality (AR) rehabilitation at home (75% of 20 sessions) and in-clinic (25% of sessions) using AR system with virtual trainer and specialized software  Comparator group: AR rehabilitation in-clinic (75% of 20 sessions) and at home (25% of sessions) with same system |
| Comparison | AR rehabilitation with mostly at-home sessions versus mostly in-clinic sessions |
| Outcomes | Motor function: Fugl-Meyer Assessment (FMA) for upper and lower extremities  Balance: Berg Balance Scale (BBS)  Gait: Functional Ambulation Category (FAC)  Activities of daily living: Barthel Index (BI)  Quality of life: 12-Item Short Form Health Survey (SF-12v2) |
| Efficacy | The experimental group receiving mostly at-home AR training showed significant improvements in upper extremity motor function, lower extremity motor function, balance, activities of daily living, and physical quality of life. The comparator group receiving mostly in-clinic training also showed improvements in most outcomes, though slightly less than the experimental group. This suggests AR rehabilitation delivered primarily in the home can be effective for chronic stroke patients. |
| Challenges | High dropout rate potentially related to COVID-19 issues  Limited ability to train mobility and balance safely at home  Need for trainer assistance early in at-home sessions  Privacy concerns with home camera monitoring  Potentially reduced social interaction and mental health quality when training alone at home |

**Table 54-** Characteristics of Study Number 54

| Study Title | Effects of transcranial direct current stimulation with virtual reality on upper limb function in patients with ischemic stroke: a randomized controlled trial |
| --- | --- |
| Study ID | Yao 2020 |
| VR type | Semi-immersive |
| Participants | 40 patients with subacute ischemic stroke from China.  31 men, 9 women.  Mean age: 64.6 years.  2 weeks to 12 months post-stroke.  Upper limb motor impairment. |
| Interventions | Experimental group (n=20): c-tDCS (2mA, 20 min) plus VR training (20 min) for 10 sessions over 2 weeks.  Control group (n=20): Sham t-DCS plus VR training (20 min) for 10 sessions over 2 weeks.  VR system: semi-immersive with mechanical ontology, feedback sensing manipulator, and large screen.  VR games for upper limb training. |
| Comparison | c-tDCS plus VR training vs sham t-DCS plus VR training |
| Outcomes | FM-UE, ARAT, BI scales  Motor impairment, motor function, ADLs |
| Efficacy | The experimental group showed significantly greater improvements in FM-UE (10.1 points), ARAT (7 points), and BI (12.8 points) compared to the control group. This suggests c-tDCS combined with VR training is more effective for improving upper limb function than VR training alone in patients with subacute ischemic stroke. |
| Challenges | This study is limited by a small sample size, single blinding, and lack of quantification of additional rehabilitation therapies. More robust studies are needed to confirm the additive benefits of c-tDCS and VR for post-stroke upper limb rehabilitation. |

**Table 55-** Characteristics of Study Number 55

| Study Title | A randomized controlled trial on the effects of occupational therapy interventions using patient-centered robot-assisted rehabilitation for functional improvement in subacute stroke patients |
| --- | --- |
| Study ID | Yoon 2021 |
| VR type | none-immersive |
| Participants | 31 patients with subacute stroke (2 weeks to 6 months post-stroke)  25 men, 6 women  Mean age: 66 years  Inclusion criteria: arm weakness from stroke, able to manipulate Wii remote  Exclusion criteria: severe comorbidities, shoulder subluxation, pacemaker |
| Interventions | Experimental group (n=10): Patient-centered robot therapy using PABLO device + conventional therapy  Control group 1 (n=11): Robot-centered robot therapy using PABLO device + conventional therapy  Control group 2 (n=10): Conventional therapy only |
| Comparison | Patient-centered robot therapy compared to robot-centered and conventional therapy |
| Outcomes | Joint range of motion, grip strength, upper limb function, ADL performance  Assessed before and after 3 weeks of intervention |
| Efficacy | The patient-centered robot therapy group showed significant improvements in shoulder, elbow, wrist, and finger range of motion, grip strength, upper limb function per Fugl-Meyer Assessment, and ADL performance per Korean Modified Barthel Index compared to the other groups. The patient-centered approach improved ADL performance by 10.8 points more than the robot-centered approach. |
| Challenges | Small sample size limits statistical power. Short 3-week intervention duration may limit treatment effect. Inability to isolate effects of robot vs. conventional therapy. Limited to subacute stroke so results may not generalize. No long-term follow-up. Limited outcome measures. Single center with few therapists reduces generalizability. Did not compare robot therapy doses or parameters. |
