# Supplementary material for: The efficacy of virtual reality for upper limb rehabilitation in stroke patients: a systematic review and meta-analysis
Source: BMC Med Inform Decis Mak. 2024 May 24;24:135. doi: 10.1186/s12911-024-02534-y (PMC11127427; doi:10.1186/s12911-024-02534-y)
Supplement: Supplementary file 4 — Supplementary Material 4 [file 12911_2024_2534_MOESM4_ESM.docx]

**Title: Funnel Plots of Outcome Measures in Virtual Reality Subgroups**

This additional file contains a series of funnel plots depicting the results of various outcome measures across full-immersive, semi-immersive, and non-immersive virtual reality subgroups. Each figure corresponds to a specific outcome measure. For each outcome measure, funnel plots are presented under both random effect and fixed effect models, providing insights into potential publication bias across different VR intervention subgroups.


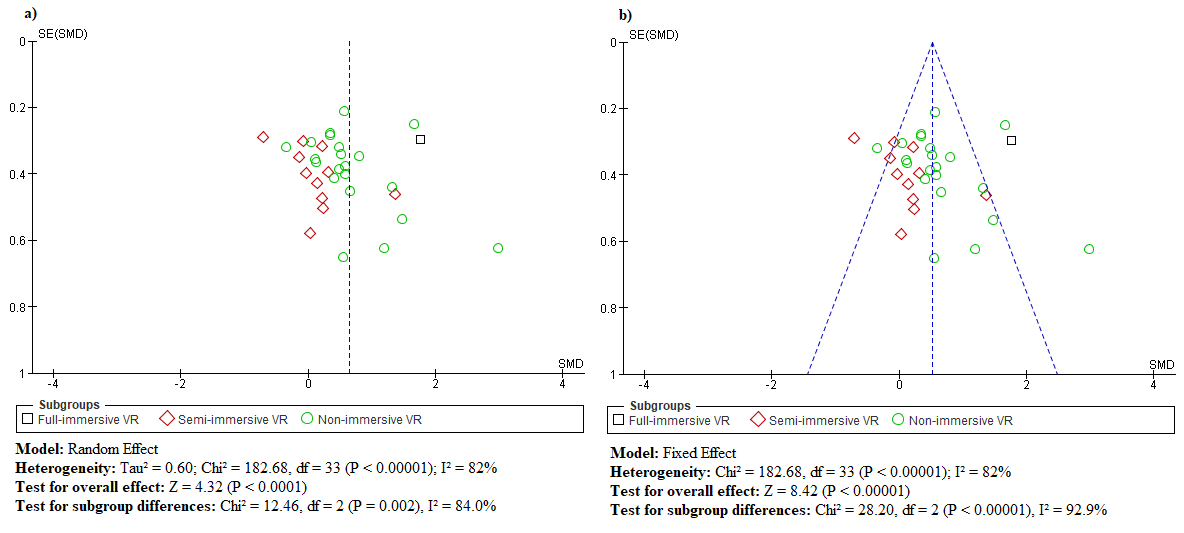


**Fig 1:** Funnel plot of Fugle-Meyer Assessment (FMA) across VR subgroups: full-immersive, semi-immersive, and non-immersive. a) Funnel plot in random effect model, b) Funnel plot in fixed effect model.


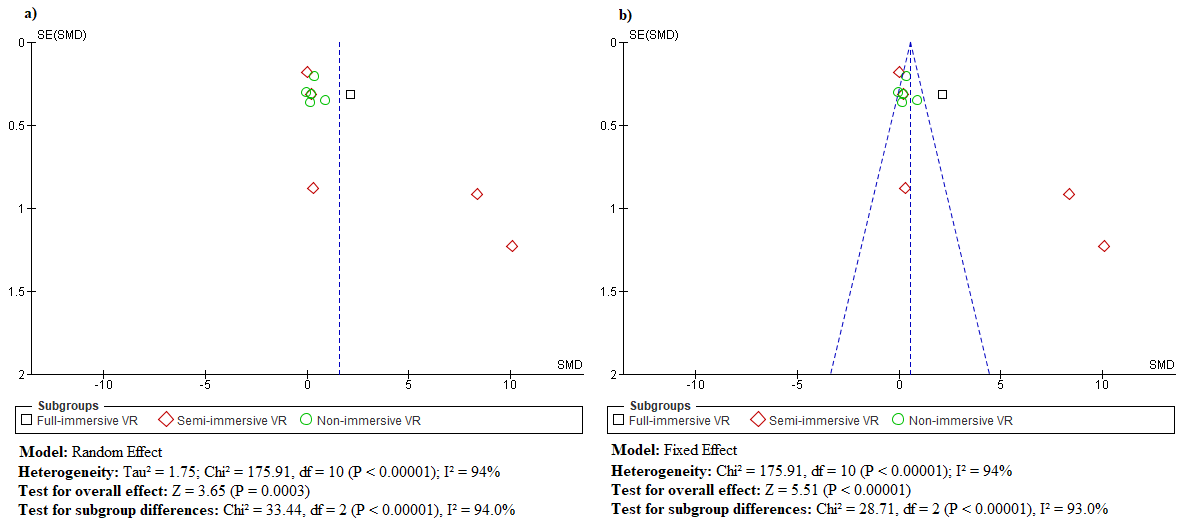


**Fig 2:** Funnel plot of Action Research Arm Test (ARAT) across full-immersive, semi-immersive, and non-immersive virtual reality subgroups.


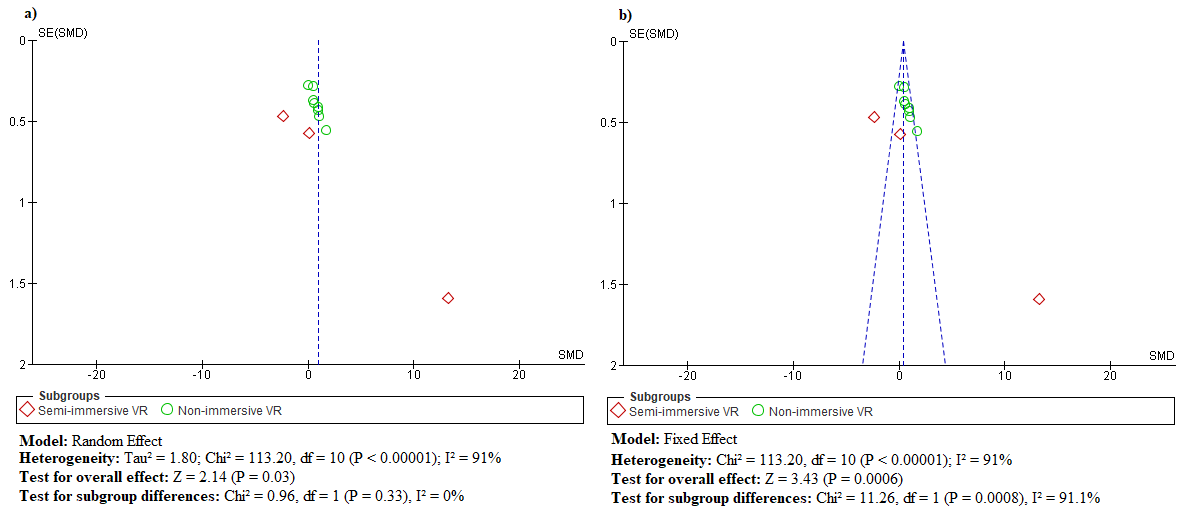


**Fig 3:** Funnel plot of Wolf Motor Function Test (WMFT) across VR subgroups: full-immersive, semi-immersive, and non-immersive. a) Funnel plot in random effect model, b) Funnel plot in fixed effect model.


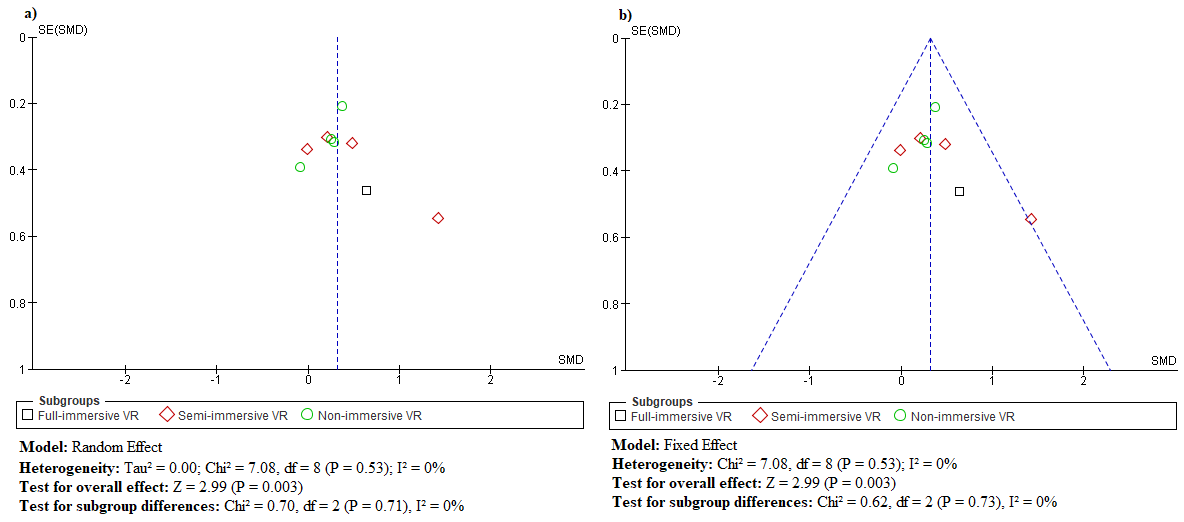


**Fig 4:** Funnel plot of Grip Strength across VR subgroups: full-immersive, semi-immersive, and non-immersive. a) Funnel plot in random effect model, b) Funnel plot in fixed effect model.


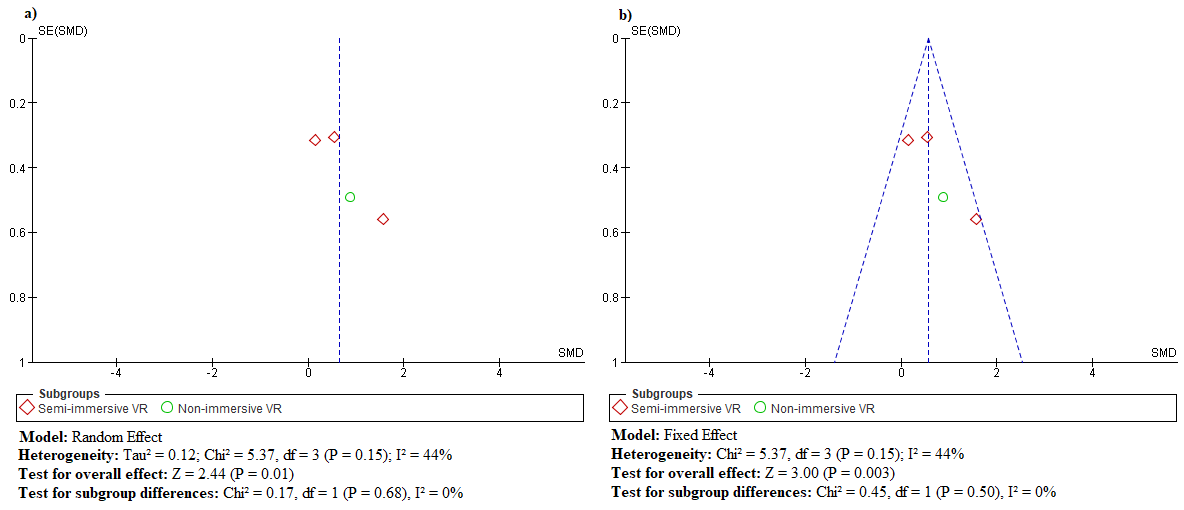


**Fig 5:** Funnel plot of Jebsen Test of Hand Function (JTHF) across VR subgroups: full-immersive, semi-immersive, and non-immersive. a) Funnel plot in random effect model, b) Funnel plot in fixed effect model.


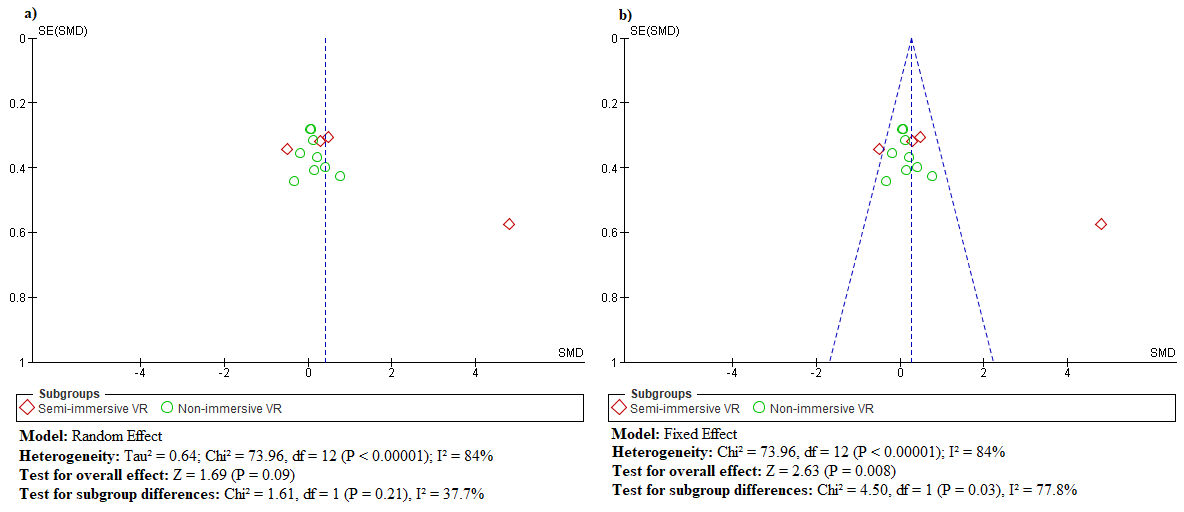


**Fig 6:** Funnel plot of Barthal Index (BI) across VR subgroups: full-immersive, semi-immersive, and non-immersive. a) Funnel plot in random effect model, b) Funnel plot in fixed effect model.


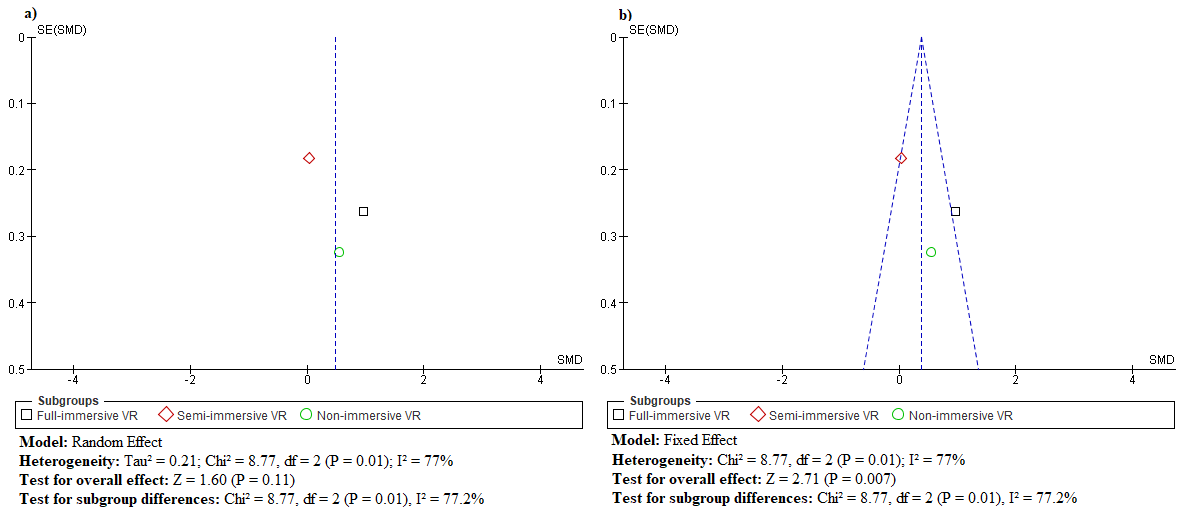


**Fig 7:** Funnel plot of Functional Independence Measure (FIM) across VR subgroups: full-immersive, semi-immersive, and non-immersive. a) Funnel plot in random effect model, b) Funnel plot in fixed effect model.


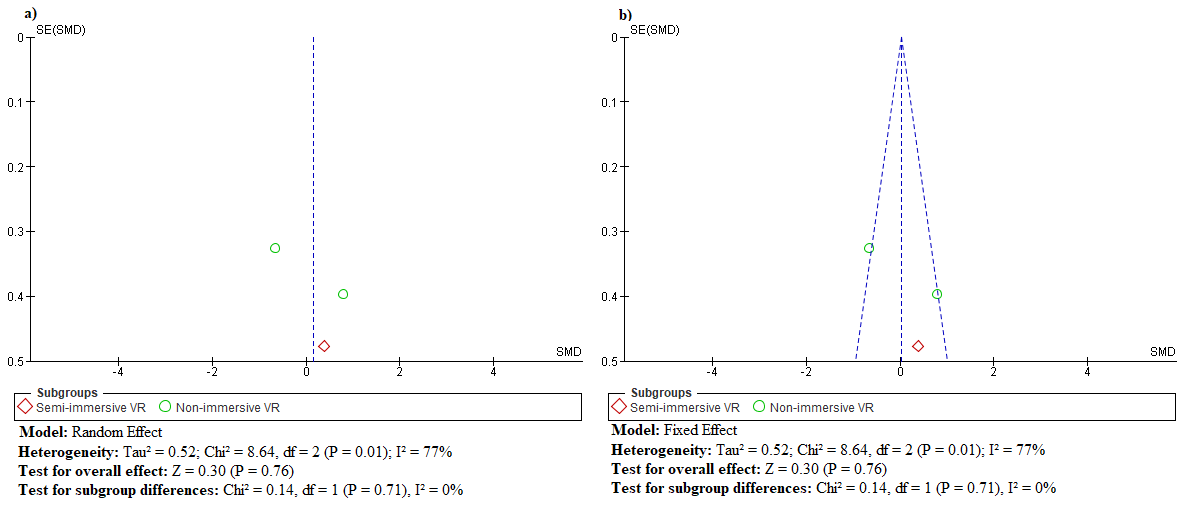


**Fig 8:** Funnel plot of Stroke Impact Scale (SIS) across VR subgroups: full-immersive, semi-immersive, and non-immersive. a) Funnel plot in random effect model, b) Funnel plot in fixed effect model.


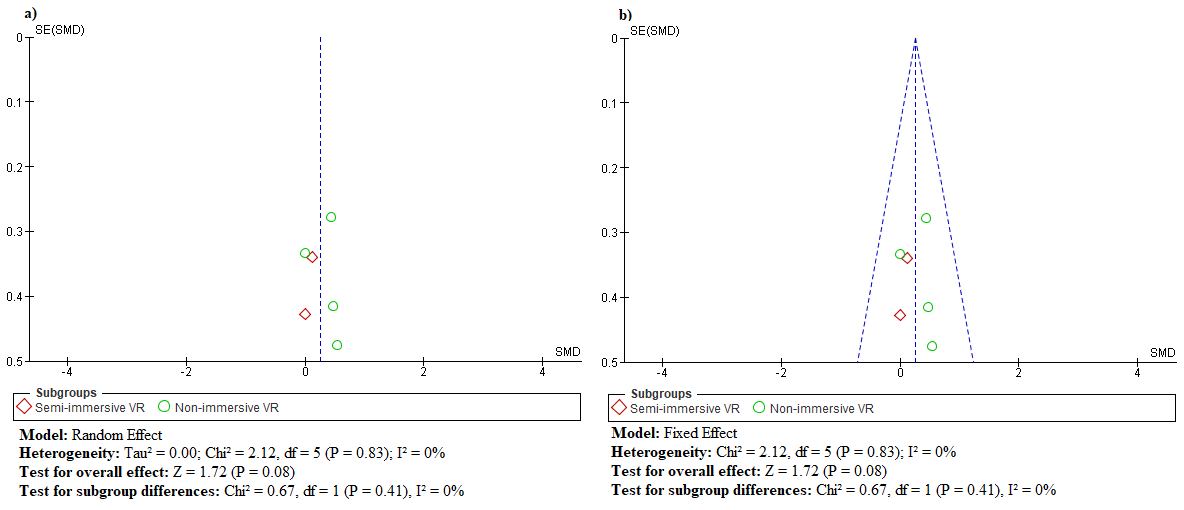


**Fig 9:** Funnel plot of Modified Ashworth Scale (MAS) across VR subgroups: full-immersive, semi-immersive, and non-immersive. a) Funnel plot in random effect model, b) Funnel plot in fixed effect model.


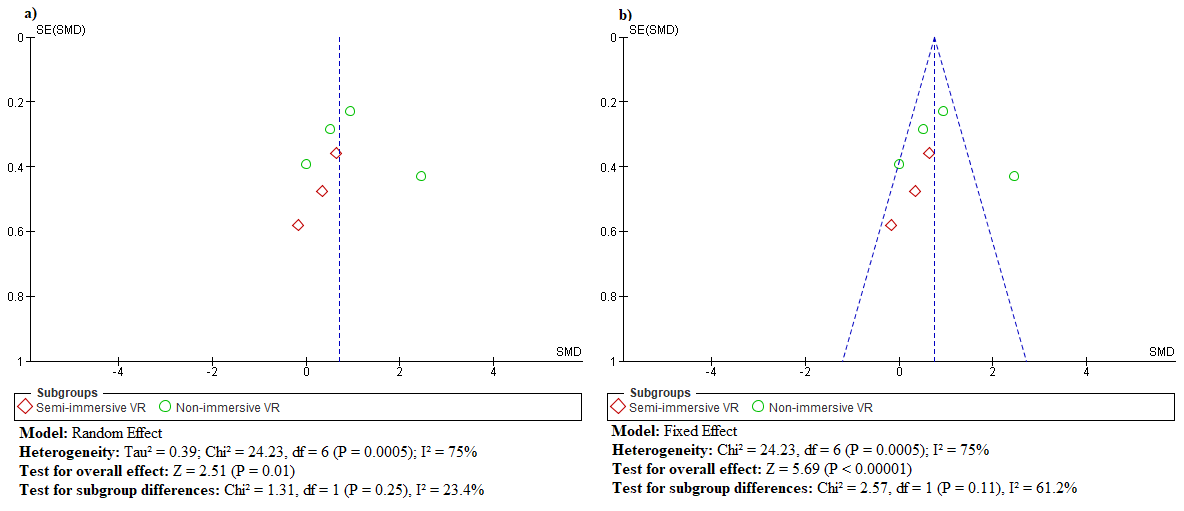


**Fig 10:** Funnel plot of Motor Activity Log – Amount of Use Scale (MAL-AUS) across VR subgroups: full-immersive, semi-immersive, and non-immersive. a) Funnel plot in random effect model, b) Funnel plot in fixed effect model.


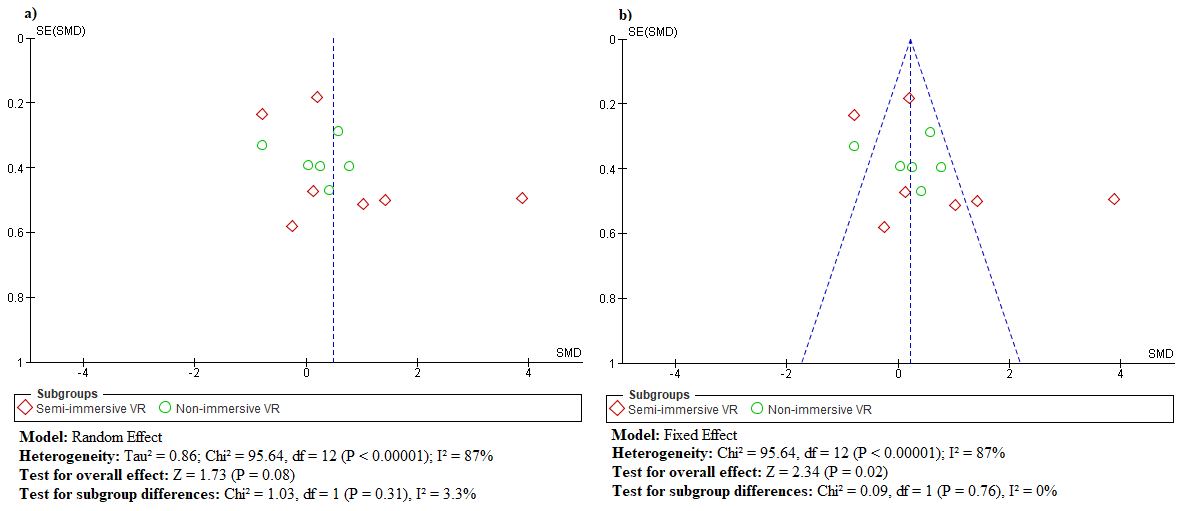


**Fig 11:** Funnel plot of Box and Block Test (BBT) across VR subgroups: full-immersive, semi-immersive, and non-immersive. a) Funnel plot in random effect model, b) Funnel plot in fixed effect model.
